# Replacement of microglia in the aged brain reverses cognitive, synaptic, and neuronal deficits in mice

**DOI:** 10.1111/acel.12832

**Published:** 2018-10-02

**Authors:** Monica R. P. Elmore, Lindsay A. Hohsfield, Enikö A. Kramár, Lilach Soreq, Rafael J. Lee, Stephanie T. Pham, Allison R. Najafi, Elizabeth E. Spangenberg, Marcelo A. Wood, Brian L. West, Kim N. Green

**Affiliations:** ^1^ Department of Neurobiology and Behavior University of California Irvine California; ^2^ Institute for Memory Impairments and Neurological Disorders (UCI MIND) Irvine California; ^3^ University College London London UK; ^4^ The Francis Crick Institute London UK; ^5^ Plexxikon Inc Berkeley California

**Keywords:** aging, colony‐stimulating factor 1 receptor, long‐term potentiation, microglia, plx5622, repopulation

## Abstract

Microglia, the resident immune cell of the brain, can be eliminated via pharmacological inhibition of the colony‐stimulating factor 1 receptor (CSF1R). Withdrawal of CSF1R inhibition then stimulates microglial repopulation, effectively replacing the microglial compartment. In the aged brain, microglia take on a “primed” phenotype and studies indicate that this coincides with age‐related cognitive decline. Here, we investigated the effects of replacing the aged microglial compartment with new microglia using CSF1R inhibitor‐induced microglial repopulation. With 28 days of repopulation, replacement of resident microglia in aged mice (24 months) improved spatial memory and restored physical microglial tissue characteristics (cell densities and morphologies) to those found in young adult animals (4 months). However, inflammation‐related gene expression was not broadly altered with repopulation nor the response to immune challenges. Instead, microglial repopulation resulted in a reversal of age‐related changes in neuronal gene expression, including expression of genes associated with actin cytoskeleton remodeling and synaptogenesis. Age‐related changes in hippocampal neuronal complexity were reversed with both microglial elimination and repopulation, while microglial elimination increased both neurogenesis and dendritic spine densities. These changes were accompanied by a full rescue of age‐induced deficits in long‐term potentiation with microglial repopulation. Thus, several key aspects of the aged brain can be reversed by acute noninvasive replacement of microglia.

## 1 INTRODUCTION

Microglia are the primary immune cells of the central nervous system (CNS), where they act as responders in the event of infection or injury. Microglia “at rest” are highly dynamic cells, constantly extending and retracting their processes to sample the local environment via a variety of receptors, including pattern recognition, purinergic, and scavenger receptors (Hanisch & Kettenmann, 2007; Nimmerjahn, Kirchhoff, & Helmchen, 2005). In addition to their immunocompetency, studies implicate microglia in maintaining tissue homeostasis and synaptic connectivity (Kettenmann, Kirchhoff, & Verkhratsky, 2013). In neurodegenerative disease or following traumatic brain injury, microglia can assume long‐lasting changes in morphology, densities, gene expression, and cytokine/chemokine production. Studies have indicated that these signals, when persistent in the brain, can lead to further harm (Dheen, Kaur, & Ling, 2007; Rice et al., 2015; Spangenberg et al., 2016).

Microglia are critically dependent upon signaling through the colony‐stimulating factor 1 receptor (CSF1R) for their survival (Elmore et al., 2014). We identified several orally bioavailable CSF1R inhibitors that noninvasively cross the blood‐brain barrier, leading to brain‐wide microglial elimination within days, which continues for as long as CSF1R inhibition is present (Dagher et al., 2015; Elmore et al., 2014). In particular, removal of CSF1R inhibition stimulates the rapid repopulation of the entire brain with new microglial cells (Elmore et al., 2014; Elmore, Lee, West, & Green, 2015; Rice et al., 2017), effectively replacing the entire microglial tissue. This process takes approximately 14–21 days to complete; thereafter, the new microglia are virtually indistinguishable (i.e., cell densities, morphologies, and gene expression profiles) from the resident microglial tissue.

Following brain injury, microglia mount a chronic inflammatory response, including robust changes in morphologies and production of neuroinflammatory species. We have shown that CSF1R inhibitor‐dependent microglial replacement essentially “resets” this “reactive” microglial population, whereby returning microglia resemble nonreactive homeostatic microglia and promote functional recovery (Rice et al., 2017). Thus, microglial elimination and repopulation may be beneficial in situations that implicate altered microglial phenotypes in impaired brain function. During aging, microglia undergo marked phenotypic and functional changes compared to the adult brain, including increased cell numbers, dystrophic morphology, impaired phagocytosis, reduced motility, exaggerated response to inflammatory stimuli [reviewed in Mosher and Wyss‐Coray (2014)], as well as altered gene expression (Galatro et al., 2017; Grabert et al., 2016; Soreq, Rose, Soreq, Hardy, & Ule, 2017). These microglia are often described as “primed” or “senescent,” and recent studies suggest that these cells may contribute to age‐related cognitive impairments and confer susceptibility to neurodegenerative disease (Blank & Prinz, 2013; Niraula, Sheridan, & Godbout, 2017; Norden, Muccigrosso, & Godbout, 2015). Here, we explore the effects of replacing the aged microglial tissue with new cells, via CSF1R inhibitor‐dependent microglial elimination and repopulation. This study demonstrates that broad reversals in many age‐related changes in the brain can be achieved utilizing microglial repopulation, including improvements in cognition, as well as neuronal/synaptic‐related gene expression, structure, and function.

## 2 RESULTS

### 2.1 CSF1R inhibitor‐dependent microglial elimination and repopulation

To illustrate the phenomenon of microglial elimination, and subsequent microglial replacement/repopulation, we treated adult (~2 months) C57BL/6 mice (*n* = 4 mice/group) with the orally bioavailable CSF1R inhibitor PLX5622 (formulated at 1,200 ppm in chow), for 7 days. PLX5622 is a more specific (i.e., the compound does not inhibit c‐Kit) sister molecule of the clinically utilized CSF1R inhibitor PLX3397 (Butowski et al., 2016; Tap et al., 2015). This treatment eliminated ~85% of microglia throughout the CNS (Supporting Information Figure S1a–c). Subsequent withdrawal of CSF1R inhibition stimulated rapid repopulation of the brain with new microglia (Supporting Information Figure S1a–c). Repopulating cells appear within 3 days of drug withdrawal. These cells are initially larger/more amoeboid (Supporting Information Figure S1b,d) and reactive for the lectin IB4 (Supporting Information Figure 1b,e), as well as other markers of myeloid cell activation (Elmore et al., 2015, 2014 ). This expression, however, dissipates over time. Repopulating cells proliferate rapidly, leading to an overshoot in microglial numbers by Day 7, which then normalizes by Day 21 (Supporting Information Figure S1a–e).

### 2.2 Microglial repopulation improves cognition in aged mice

**Figure 1:**
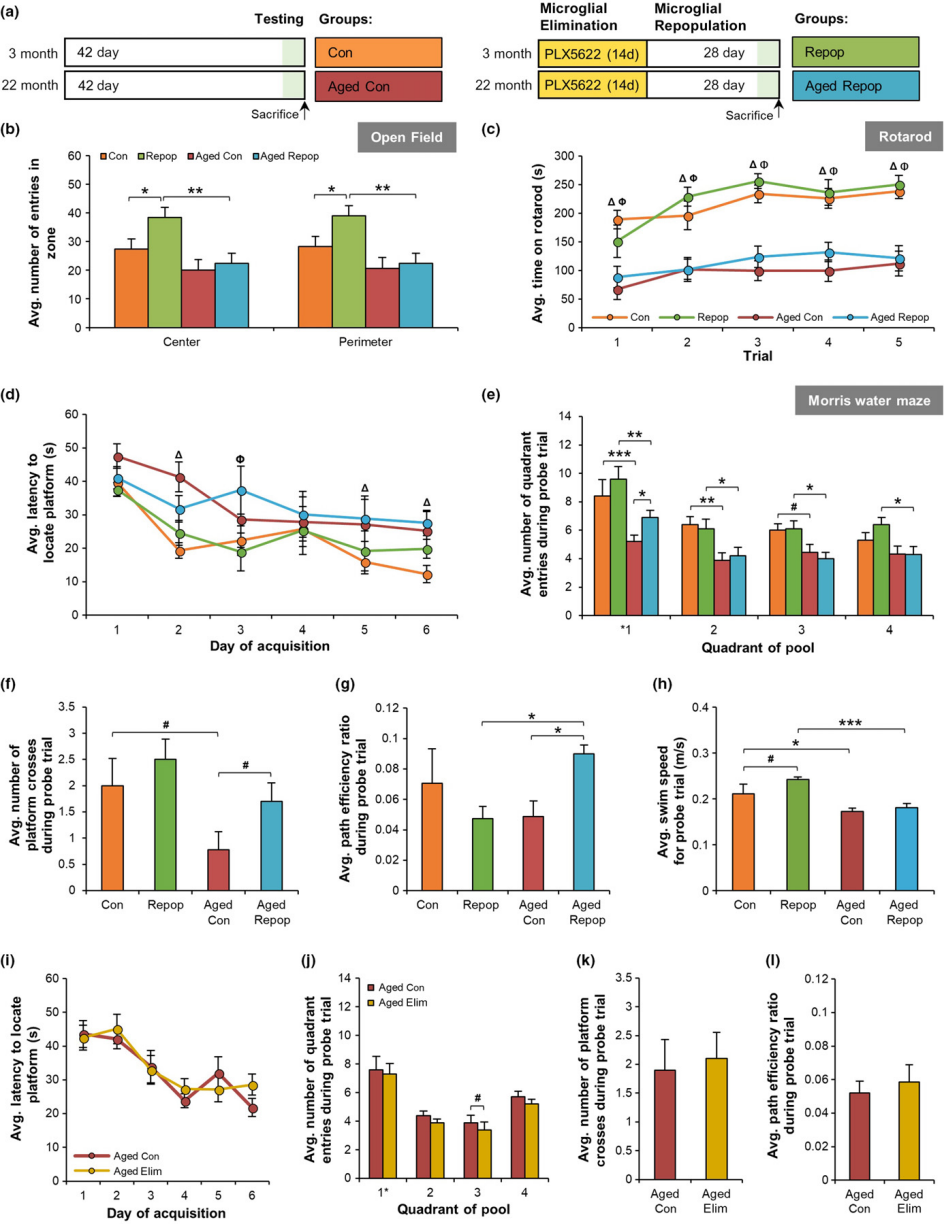
Microglial Replacement Improves Cognition in Aged Mice. (a) Experimental design for (a–h). Treatment groups are as follows (*n* = 8–10 mice/group): young control (Con), young microglia‐repopulated (Repop), aged control (Aged Con), and aged microglia‐repopulated (Aged Repop). (b) Open field analyses of number of line crosses between the center zone and the perimeter. (c) Rotarod performance. (d) Acquisition data from training days 1–6 of the Morris Water Maze. (e–h) Morris Water Maze probe trial, conducted 24 hr after the last training trial showing the number of correct quadrant entries (e), platform crosses (where * indicates the location of the platform during acquisition) (f), path efficiency (g), and swim speed (h). (i–l) Aged (~22 months) mice were provided control chow or PLX5622 (1,200 ppm in chow) for 14 days to eliminate microglia, the same timeline used in (a). The following treatment groups were: Aged Con and aged eliminated (Aged Elim) (*n* = 10/group). (i–l) Morris Water Maze performance for acquisition (i), latency to locate the platform in probe trial (i), the number of platform crosses during the probe trial (k), and the path efficiency during the probe trial (l). Data presented as means ± *SEM*. Statistical significance indicated as *p < 0.05, **p < 0.01, and ***p < 0.001 with statistical trends as ^#^ *p* < 0.10. Comparisons for (c) and (d): ^Ω^Con versus Repop; ^Δ^Con versus Aged Con; ^Φ^Repop versus Aged Repop; ^Ψ^Aged Con versus Aged Repop

Converging evidence indicates that the aged brain contains primed and/or senescent microglia (Dilger & Johnson, 2008; Harry, 2013; Mosher & Wyss‐Coray, 2014), which may contribute to age‐related cognitive deficits and altered brain function (Norden et al., 2015). Therefore, we sought to explore the effects of replacing these resident “aged" microglia with new cells, via administration and withdrawal of CSF1R inhibitors. Young (~3 months) or aged (~22 months) mice were treated for 14 days with PLX5622 to eliminate resident microglia and then removed from inhibitor diet to stimulate repopulation. Behavioral and cognitive testing [on measures of anxiety (open field; OF), motor function (rotarod), and spatial learning and memory (Morris water maze, MWM)] was conducted 28 d following the start of microglial repopulation (Figure 1a). In OF, no differences in aged control compared to aged‐repopulated mice were observed, but there were increases in exploratory behavior in young repopulated mice (*p* = 0.030; Figure 1b). A main effect of age was observed in measurements of motor function via rotarod performance and MWM acquisition, in that aged mice exhibited motor impairments relative to young mice as seen by increased time spent on the rotarod apparatus (*p* < 0.0001, Figure 1c) and increased time to locate the MWM platform (*p* < 0.001, Figure 1d), respectively. In particular, microglial repopulation in aged mice improved spatial memory performance in the MWM probe trial as seen by an increased number of correct quadrant entries (*p* = 0.049, Figure 1e), a trend for increased number of platform crosses (*p* = 0.055, Figure 1f), and improved path efficiency (*p* = 0.019, Figure 1g), in comparison with aged control mice**. **No significant differences were found in swim speed between aged control and aged‐repopulated mice (Figure 1h), highlighting the beneficial effects of repopulation on memory, but not motor function, in aged mice. As microglial repopulation cannot occur without prior microglial elimination, in a separate experiment, we also assessed the behavior of aged mice depleted of microglia for 14 days (Figure 1i–l). Following CSF1R‐inhibitor treatment, we found no differences in behavioral or cognitive function in aged microglia‐eliminated mice compared with aged controls. Thus, it appears that the act of microglial elimination in combination with repopulation/replacement, rather than elimination alone, conveys cognitive benefits to aged mice.

### 2.3 Microglial repopulation in aged mice restores microglial cell densities and morphologies to young adult levels

**Figure 2:**
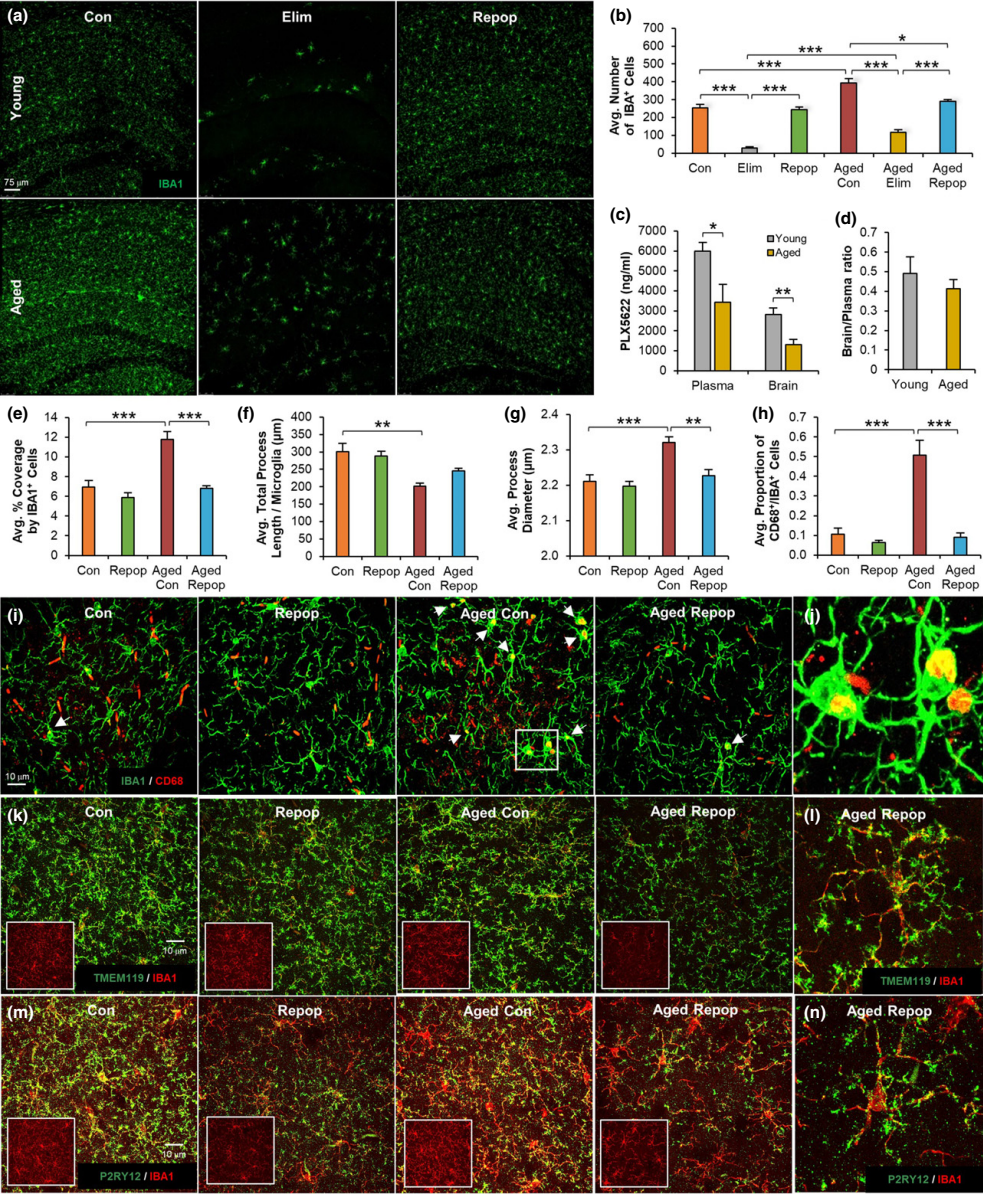
Microglial Replacement Restores Cell Numbers and Morphologies. (a) IBA1^+^ immunostaining in hippocampal sections from young control (Con), young microglia‐repopulated (Repop), aged control (Aged Con), and aged microglia‐repopulated (Aged Repop) groups. (b) Quantification of (a). (c) Pharmacokinetic (PK) analysis of PLX5622 concentrations in plasma and brain. (d) Brain/Plasma ratio of (c). (e) Percent coverage of hippocampal area by IBA1^+^ cells. (f) Morphological analyses of hippocampal microglia for total process length per microglia. (g) Average microglial process diameter. (h) Quantification of (i). (i) Immunostaining for IBA1^+^ (green)/CD68^+^ (red) colocalized cells in hippocampal sections. (j) Zoomed image of IBA1^+^/CD68^+^ cells in Aged Con group. (k) Immunostaining for IBA1 (red channel) and TMEM119 (green channel) staining shown as merge, with IBA1 staining alone shown in insert. (l) Magnified image of Aged Repop. (m) Immunostaining for IBA1 (red channel) and P2RY12 (green channel) staining shown as merge, with IBA1 staining alone shown in insert. (n) Magnified image of Aged Repop. Data presented as means ± *SEM*. Statistical significance indicated as *p < 0.05, **p < 0.01, and ***p < 0.001 with statistical trends as ^#^ *p* < 0.10

Immunohistochemical examination of hippocampal tissue for the myeloid cell marker IBA1 showed that 14 days treatment with PLX5622 eliminated ~90% and ~70% of microglia in young and aged mice, respectively (Figure 2a,b). Concomitant with reductions in microglial elimination efficiency, we observed decreased levels of PLX5622 in the plasma (*p* = 0.029) and brains (*p* = 0.009) of aged mice compared with young mice (Figure 2c), but similar brain/plasma ratios (Figure 2d). Aged mice maintained their initial body weights (Supporting Information Figure S2), while young treated mice showed an increase in body weight over the two‐week treatment period (*p* < 0.001). Aged control mice showed an increase in the density (or cell number) of IBA1^+^ cells in the hippocampus (~54% increase; *p* < 0.001, Figure 2a,b), higher microglial coverage (*p* < 0.001, Figure 2e), as well as a shortening (*p* = 0.002, Figure 2f) and thickening (*p* < 0.001, Figure 2g) of microglial processes compared with young controls. In particular, 28 days after inhibitor withdrawal, age‐induced changes in microglial cell density and morphology were normalized, in which aged‐repopulated mice exhibited similar phenotypes to young controls (i.e., reduced IBA1^+^ cell number [*p* = 0.02, Figure 2b], reduced IBA1 coverage [*p* < 0.001, Figure 2e], and reduced process diameter [*p* = 0.004, Figure 2g] compared with aged controls. In the aged control brain, we observed an increased proportion of CD68^+^ (a lysosomal marker indicative of microglial priming)/IBA1^+^ cells compared with young controls (*p* < 0.001, Figure 2h–j). However, following microglial replacement, this microglial‐associated CD68 expression was significantly reduced (*p* < 0.001, Figure 2h). As resident microglia share expression of IBA1 with peripherally‐derived macrophages, we also conducted additional immunohistochemical stains using the microglia‐specific markers TMEM119 and P2RY12, which distinguish microglia from peripheral myeloid cells (Bennett et al., 2016). Here, we show that repopulated microglia display high expression of both TMEM119 and P2RY12 (Figure 2k–n), demonstrating that repopulated microglia are indeed resident microglia and do not originate from peripherally‐derived myeloid cells. These findings are in line with previous studies from our laboratory (Elmore et al., 2014) and others (Cronk et al., 2018; Huang et al., 2018) showing that repopulating microglia are CNS‐derived and not from peripheral infiltrates. Thus, CSF1R inhibitor‐directed replacement of microglia in the aged brain restores age‐induced changes in microglial numbers, morphologies, and CD68 puncta, reverting the microglial phenotype to that observed in the young brain, at least out to 28 days.

### 2.4 Microglial repopulation does not alter response to neuroinflammatory stimuli

**Figure 3:**
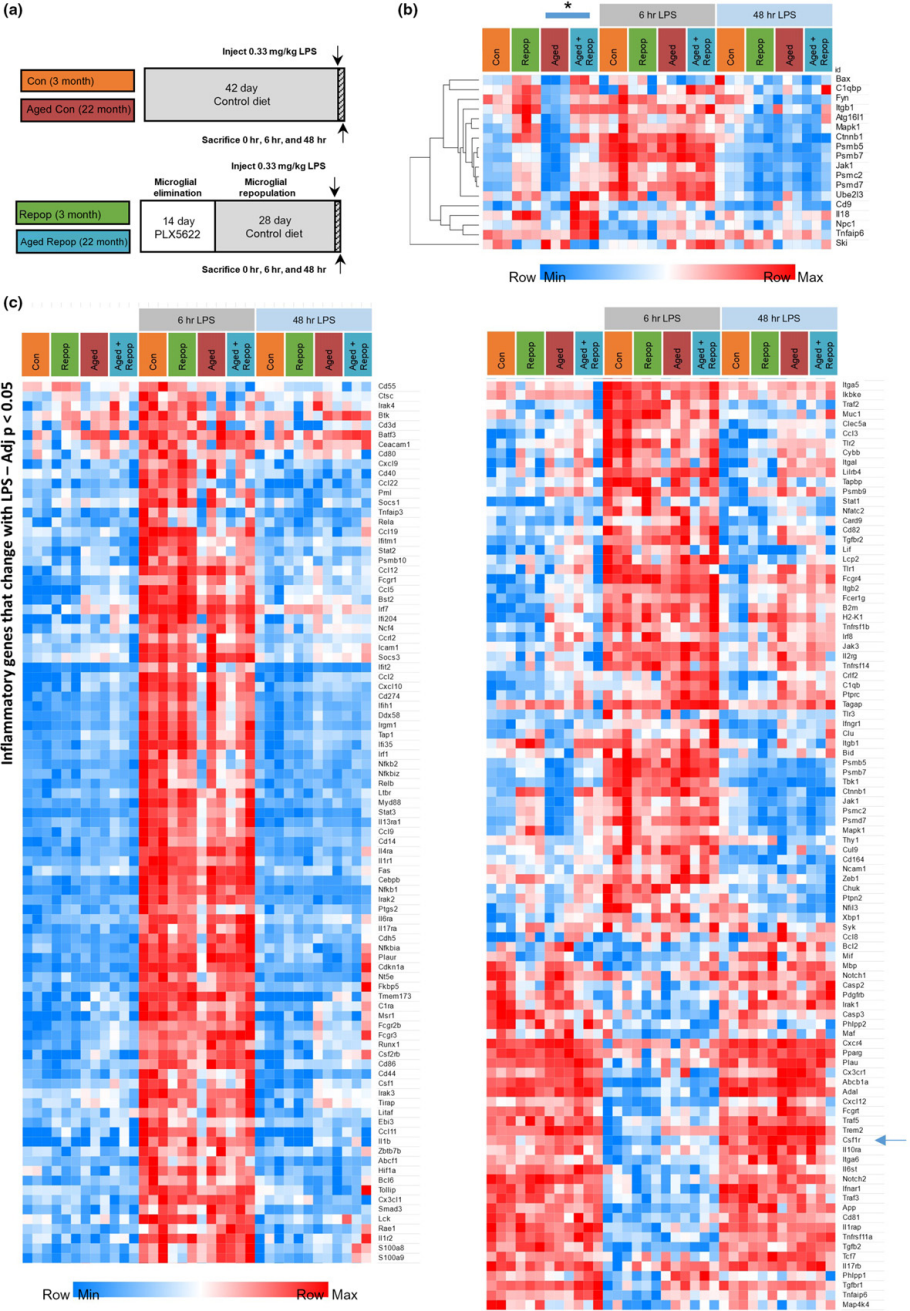
Microglial Replacement does not Alter Neuroinflammatory Gene Expression in Response to LPS. (a) Experimental design (*n* = 3 mice/group). (b, c) Gene expression for immune genes assessed via Nanostring Mouse Immune panel (550 genes). (b) Heatmap of differentially expressed genes between Aged Con and Aged Repop mice (adj. *p* < 0.05) at baseline (0 hr LPS) shown for all groups. (c) Heatmap of differentially expressed genes due to LPS at either 6 or 48 hr in Con or Aged Con mice (adj. *p* < 0.05) shown for all groups. Normalized raw expression values are depicted (light blue to red). All genes and values can be found in Table S1

Previous reports show that microglial priming or increased reactivity during aging does not reflect an enhanced basal level of inflammation, but rather an exaggerated inflammatory response to immune challenge (Niraula et al., 2017). To address and characterize the response to immune challenge in the aged brain following repopulation, we administered LPS to young (~4 months) and aged (~24 months) mice with and without 28 days of microglial repopulation (Figure 3a). Inflammatory responses were measured via gene expression using a Nanostring Immunology panel (550 genes). All gene expression counts are found in Supporting Information Table S1. Despite notable changes in microglia numbers and morphologies, only 18 genes were found to be differentially expressed between aged and aged‐repopulated mice (Figure 3b) at baseline (i.e., prior to LPS injection). Moreover, most of these genes were also changed in young mice with repopulation. Of these genes, *Fyn* was the only gene whose expression was found to be altered by aging and restored with repopulation. Next, we examined the effects of peripheral LPS administration in mice after both the acute (6 hr post‐LPS) and resolution (48 hr post‐LPS) stages of immune challenge. Here, we found that a robust inflammatory response was elicited in all groups at 6 hr, which resolved by 48 hr (Figure 3c; heatmap of significantly altered genes with LPS administration; adj. *p* < 0.05 for young or aged mice). In comparing aged to aged‐repopulated mice, only a single differentially expressed gene (*Cx3Cr1*) was detected, whereby expression was reduced at 6 hr post‐LPS in aged repopulation compared with aged controls (*p* = 0.023). Thus, acute microglial replacement does not alter the response to peripheral LPS challenge, suggesting that the aged environment (rather than microglia) extrinsically determines inflammation‐related gene expression both at baseline and in response to an immune challenge.

### 2.5 Microglia repopulation restores expression of genes associated with cytoskeletal remodeling and neural processes

**Figure 4:**
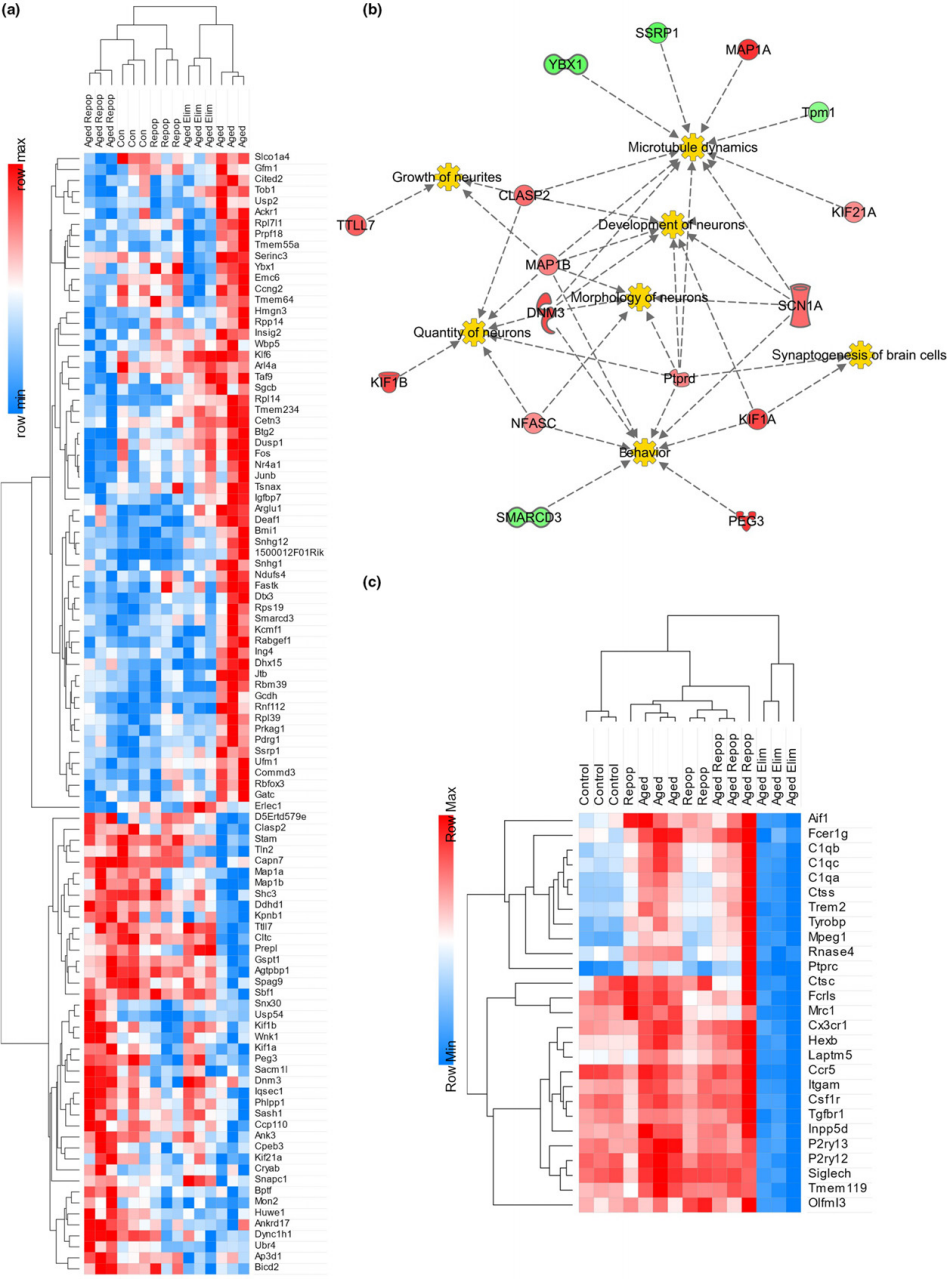
Microglial Replacement Alters Genes Associated with Actin Cytoskeleton Remodeling and Synaptic Function. RNA‐Seq analysis was performed (*n* = 3 mice/group) on brains from young control (Con), young microglia‐repopulated (Repop), aged control (Aged Con), aged microglia‐eliminated (Aged Elim), and aged microglia‐repopulated (Aged Repop) mice following behavioral testing. (a) Hierarchical clustering on gene level counts of differentially expressed genes (adj. *p* < 0.025 for Aged Con vs. Aged Repop) as a function of experimental treatment. Standardized count values are depicted by a heatmap (light blue to red). (b) Pathway analysis revealed that genes altered by repopulation in aged mice converged on areas of synaptogenesis and neurogenesis. (c) Selected microglial expressed genes shown as a heatmap

Previous studies have attributed inflammation to age‐related cognitive deficits. However, given the lack of changes in inflammatory‐related genes seen in this study, we were interested in utilizing nonbiased gene expression analysis, via RNA‐seq, to identify the underlying mechanism for memory improvements following repopulation in aged mice. To that end, tissue from control and microglia‐repopulated young (~4 months) and aged (~24 months) mice, as well as a cohort of aged microglia‐eliminated animals, was collected for RNA‐seq analysis. In total 689 differentially expressed genes were detected between all groups (adj. *p* < 0.05). The top differentially expressed genes between aged and aged‐repopulated brains (adj. *p* < 0.025) are displayed as a heatmap (Figure 4a). Hierarchical clustering demonstrated that the gene expression profile of aged‐repopulated brains resembled young control brains, rather than aged control brains, with aged microglia‐eliminated brains exhibiting an intermediate gene expression profile between aged brains and young controls for many genes. The majority of genes affected by microglial replacement in the aged brain are expressed predominantly by neurons, rather than glia. Most of the downregulated genes in aging that were subsequently restored back to young control levels with microglial replacement are associated with actin cytoskeleton remodeling (*Map1a*, *Map1b*, *Prepl*, *Ttll7*, *Clasp2*, and *Nfasc*) or processes involved in neuronal and synaptic function—including endocytosis (*Cltc)* and microtubule transport (*Kif1a*, *Kif1b*, *Kif21a, Dync1h1, and Dnm3)*—all important for synaptic vesicle release. Indeed, integrated pathway analysis identified alterations in pathways associated with *behavior*, *development of neurons*, *microtubule dynamics*, *synaptogenesis*, and others (Figure 4b). Selected microglia‐expressed genes are displayed as a heatmap (Figure 4c), showing broad reductions in their expression with elimination, and a lack of changes in myeloid cell gene expression between aged and aged‐repopulated brains. Thus, CSF1R inhibitor‐directed replacement of microglia in the aged brain has profound effects on gene expression related to neural processes, restoring many actin cytoskeleton and synaptic function‐related genes to young adult levels.

### 2.6 Microglia regulate neurogenesis

**Figure 5:**
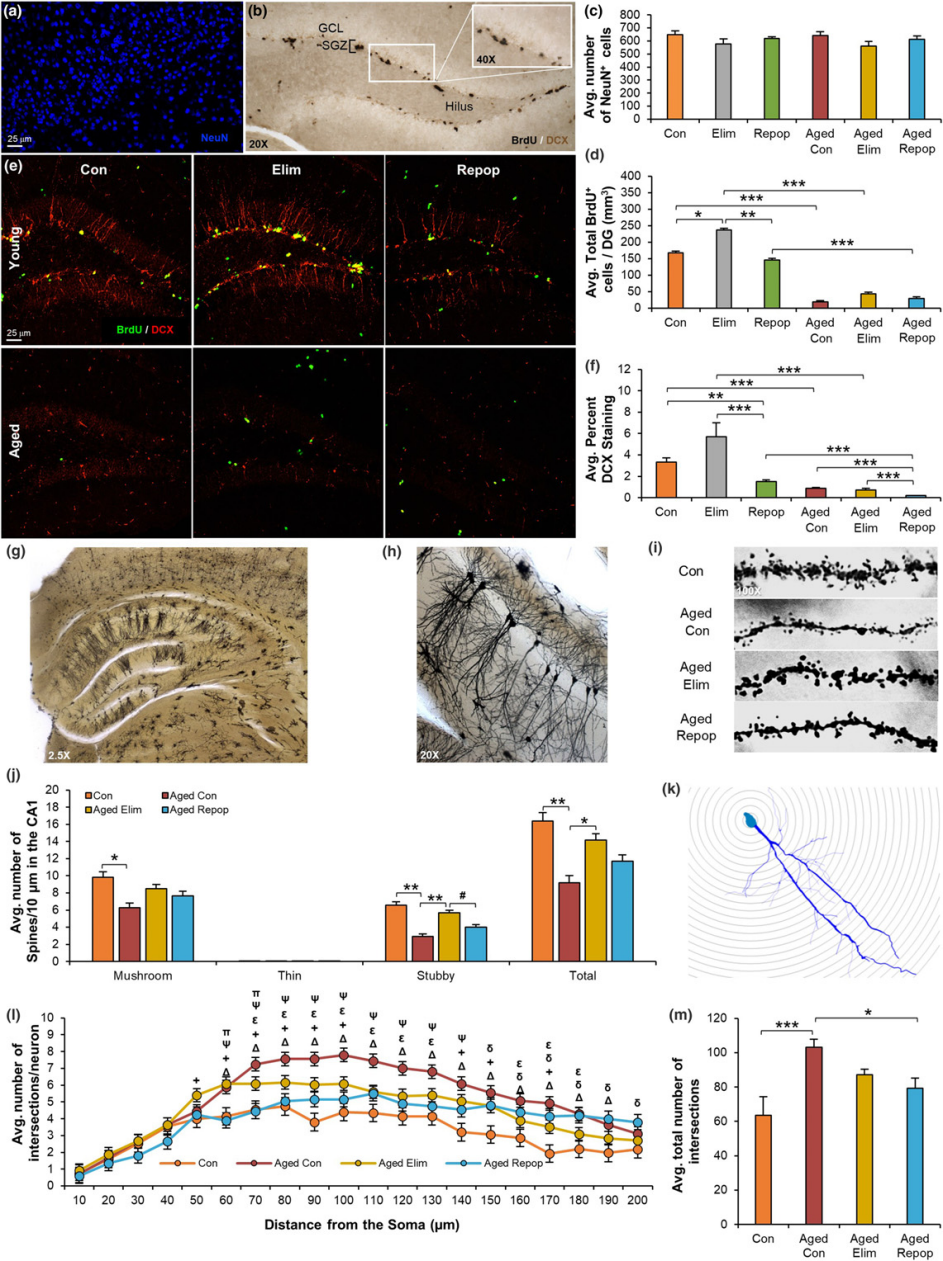
Microglial Elimination and Replacement Modifies Neurogenesis and Neuronal Morphologies. (a) Immunostaining for NeuN (neurons) in cortices from young control (Con), young eliminated (Elim), young microglia‐repopulated (Repop), aged control (Aged Con), aged eliminated (Aged Elim), and aged microglia‐repopulated (Aged Repop) mice. (c) Quantification of (a). (b) Representative image of 5’bromo‐2’‐deoxyuridine (BrdU) staining. (d) Quantification of BrdU^+^cells in the dentate gyrus (DG). (e) Immunostaining for BrdU (green) and doublecortin (DCX, red) in the DG. (f) Quantification of DCX from (e). (g–m) Golgi staining was performed (*n* = 3–5 mice/group) on Con, Aged Con, Aged Elim, and Aged Repop mice. Representative 2.5×, 20×, and 100× images of the hippocampus, demonstrating impregnation of neurons (g), dendritic branches (h), and CA1 pyramidal neurons (i) with Golgi stain. (j) Quantification of dendritic spine densities from CA1 neurons. (k) Representative image of a traced neuron and Sholl ring analysis. (l) Average number of dendritic intersections, plotted as distance from soma. (m) Treatment averages from (l). Data presented as means ± *SEM*. Statistical significance indicated as *p < 0.05, **p < 0.01, and ***p < 0.001 with statistical trends as ^#^ *p* < 0.10. Comparisons for (l): ^Δ^Con versus Aged Con; ^+^Con versus Aged Elim; ^δ^Con versus Aged Repop; ^ε^Aged Con versus Aged Elim; ^Ψ^Aged Con versus Aged Repop; ^π^Aged Elim versus Aged Repop

To expand on our pathway analysis findings, we next investigated the effects of microglial repopulation on neural processes. No differences in cortical neuronal numbers (NeuN^+^cells) were seen between any groups (Figure 5a,c). Neurogenesis in the dentate gyrus (DG) was assessed via administration of the thymidine analogue bromodeoxyuridine (BrdU), which is incorporated into the DNA of dividing cells, as well as staining for doublecortin (DCX), a marker of newly born neurons. A decline in neurogenesis is commonly observed in aging, and studies have indicated that this decline is in part due to chronically activated microglia (Barrientos, Kitt, Watkins, & Maier, 2015; Norden et al., 2015). As a result, aged mice exhibit markedly reduced neurogenesis, as seen by decreased levels of BrdU^+^ cells (*p* < 0.001, Figure 5b,d–e) and DCX staining (*p* < 0.001, Figure 5b,e–f) in the subgranular zone (SGZ) of the DG compared to young mice. Microglial elimination increased neurogenesis in young (BrdU: *p* = 0.041, tendency in DCX: *p* = 0.139) and aged mice (tendency in BrdU: *p* = 0.139, Figure 5b,d–f). However, these increases in neurogenesis with microglial elimination were restored to control levels following repopulation. Thus, these findings indicate that microglia can dynamically regulate the rate of neurogenesis.

### 2.7 Microglial elimination and repopulation in aged mice alter dendritic spine densities and neuronal complexities

Given the changes in the expression of genes associated with *growth of neurites*, *morphology of neurons,* and s*ynaptogenesis*, we performed Golgi stains (Figure 5g), allowing for quantification of neuronal complexities (Figure 5h) and dendritic spine densities in CA1 neurons (Figure 5i). Aging reduced total CA1 dendritic spine densities (*p* = 0.009, Figure 5j), including both mushroom (*p* = 0.041) and stubby (*p* = 0.002) spines, which are considered to be the more functional/mature connections between neurons, compared with thin or filopodia spines (Parnass, Tashiro, & Yuste, 2000). Elimination of microglia for 14 days was sufficient to increase total spine densities (*p* = 0.042), demonstrating that microglia play a significant role in regulating dendritic synaptic densities, even in the aged brain. Subsequent microglial replacement in aged mice normalized stubby spine numbers to that of aged controls (Figure 5j), indicating that the new microglia in the aged brain actively sculpt the synaptic landscape. To explore neuronal complexities, we utilized Sholl analysis, overlaying concentric circles on traced neurons (Figure 5k–m). Aging increased CA1 neuronal complexity, with neurons from aged control mice displaying significantly more intersections than those from young mice (*p* < 0.001, Figure 5l,m). Similar results have been reported in rat hippocampal neurons (Pyapali & Turner, 1996) and may be a compensatory mechanism to counteract reduced spine numbers during aging. In an important way, both microglial elimination (tendency: *p* = 0.105), and subsequent repopulation (*p* = 0.017), appear to normalize the number of neuronal dendrite intersections in aged mice to that of young mice (Figure 5l–m).

### 2.8 Microglial repopulation in aged mice rescues deficits in long‐term potentiation

**Figure 6:**
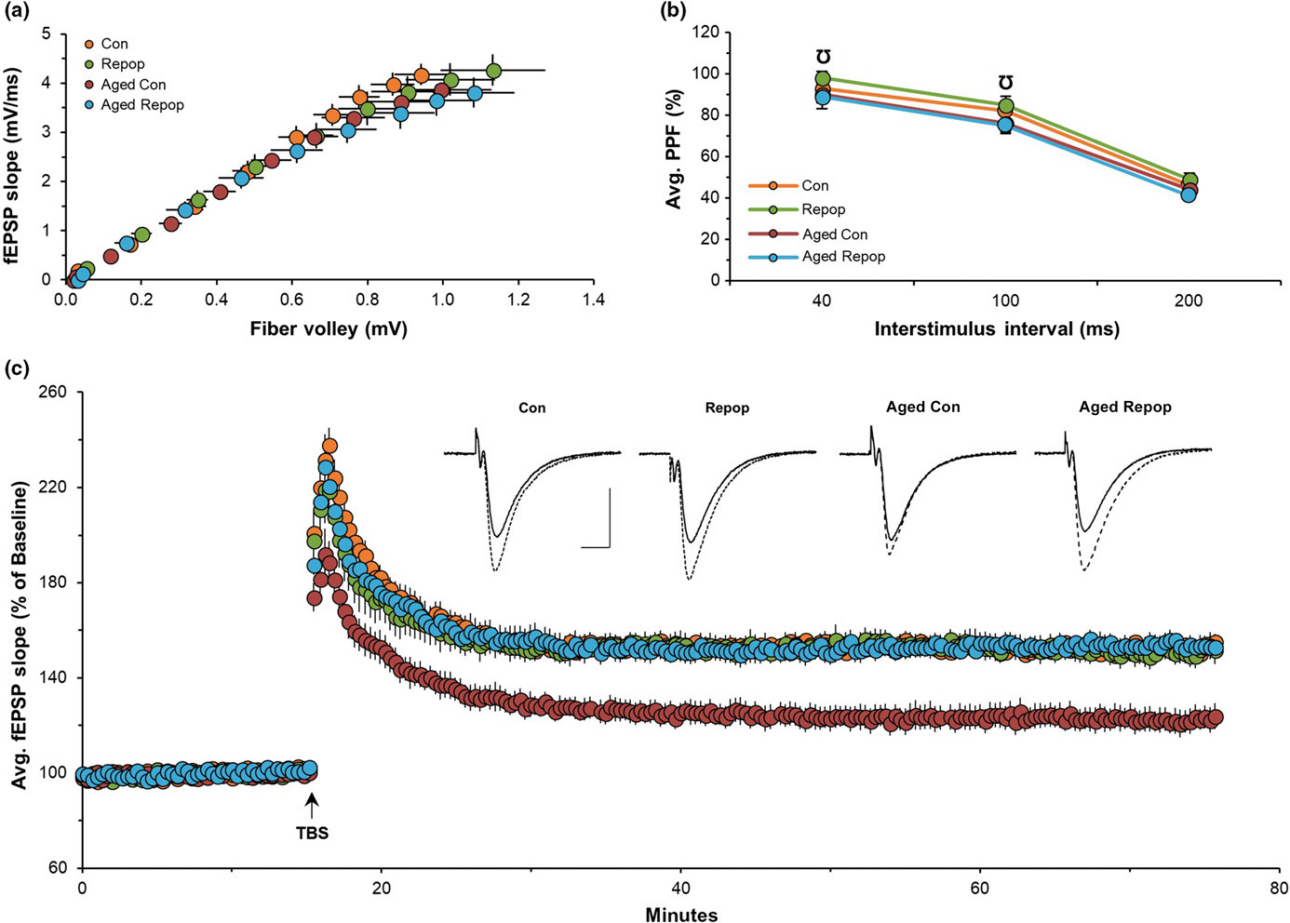
Microglial Replacement in Aged Mice Completely Reverses Impairments in Long‐Term Potentiation. (a–c) Field excitatory postsynaptic potential (fEPSPs) recordings in the CA1b stratum radiatum of hippocampal slices from young control (Con), young microglia‐repopulated (Repop), aged control (Aged Con), and aged microglia‐repopulated (Aged Repop) mice (*n* = 8–9 slices/group). (a) Input/output curves. (b) Paired‐pulse facilitation (PPF). (c) Long‐term potentiation (LTP) was induced using theta burst stimulation (TBS) with analysis of 70–80 min. Inset: Representative traces of field responses during baseline (solid line) and 1 hr after TBS (dotted line). Scale bar: 1 mV per, 5 ms. Data presented as means ± *SEM*. Comparisons for (b): ^Ʊ^Repop versus Aged Repop

Given that microglial repopulation reversed age‐induced changes in actin cytoskeleton gene expression and neuronal morphology, as well as expression of genes involved in neuronal/synaptic function and plasticity, we sought to explore the functional consequences of microglial repopulation on synaptic plasticity. We performed direct measurements of long‐term potentiation (LTP) using theta burst stimulation (TBS) in the CA1b stratum radiatum of hippocampal slices of control, repopulated, aged, and aged‐repopulated mice. First, the effect of treatment on axon excitability was tested by generating input/output curves that measure the amplitude of the presynaptic fiber volley compared to the slope of the field excitatory postsynaptic potential (fEPSP) responses across a range of stimulation currents. No treatment differences were observed (Figure 6a). Next, we evaluated transmitter release kinetics using paired‐pulse facilitation (PPF), and again, no measurable group differences were observed at any given stimulus interval (Figure 6b). These data indicate that age and microglial state (resident/control cells vs. new/repopulated cells) did not alter baseline measures of synaptic transmission. At last, LTP was examined following a single train of five theta bursts to Schaffer collateral–commissural projections, which is the threshold for inducing stable potentiation in mice (White et al., 2016). TBS produced a robust and immediate potentiation in control brain slices, which briefly decayed over a 10–15‐minute period, to stabilize at ~70% above the pre‐TBS baseline (Figure 6c). Slices from young repopulated mice exhibited normal short‐ and long‐term potentiation, with no differences compared to controls. Most importantly, hippocampal slices from aged control mice exhibited profound impairments in LTP, which were completely rescued to control levels in slices from aged mice with repopulated microglia (Figure 6c). These findings indicate that replacement of microglia in aged brains can restore the neuronal physiological processes underlying memory to that of young controls.

## 3 DISCUSSION

Microglial activation is a hallmark of aging and studies have shown that microglial phenotype and function are altered in the aged brain [reviewed in (Mosher & Wyss‐Coray, 2014)]. To explore how the replacement of aged microglia with new cells affects and shapes the resultant tissue, we employed a method of CSF1R inhibitor‐induced microglial elimination and repopulation. While we were able to efficiently eliminate the vast majority of microglia in both young and aged brains, we found an increased number of surviving microglia in the hippocampus of aged mice. Measurements of the chow‐administered PLX5622 in both plasma and brain tissue revealed a comparable reduction in inhibitor levels in aged mice, reflecting either reduced chow‐intake with aging, an increased metabolism and excretion of the compound, or an altered sensitivity of microglia with age. Recent data indicate that repopulating microglia with PLX5622 mainly derive from proliferation of surviving microglia (Huang et al., 2018), suggesting that regardless of the comparative extent of microglial depletion, the source of the resultant tissue is the same. Moreover, cell proliferation can reset (Bufalino & van der Kooy, 2014; Unruh, Slaughter, & Li, 2013) and rejuvenate (Chishti et al., 2013; Pattabiraman & Kaganovich, 2014) cellular phenotypes. Superficially the new microglial tissue mirrors that found in the young adult brain, as opposed to the aged tissue it replaces. Age‐related increases in microglial numbers, as well as shorter, thicker processes, were reversed with microglial replacement. Furthermore, the proportion of CD68^+^/IBA1^+^ microglia was dramatically reduced in aged mice with repopulated microglia, comparable to that of young controls. Therefore, it appears that following the act of microglia replacement, the newly repopulated microglia in the aged brain maintain the morphological properties of cells found in the young adult brain. The physical characteristics of the repopulated microglial tissue were explored 28 days following inhibitor withdrawal; as such, it is currently unknown if these phenotypes would persist or change with more time and require further investigation.

As microglia mediate neuroinflammatory responses through the production of a variety of signaling molecules, including cytokines and chemokines, which can themselves modulate cognition (Barrientos et al., 2015; Patterson, 2015), we sought to explore changes in inflammatory‐related genes with microglial repopulation in aging. We found that microglial replacement in the aged brain did not affect expression of immune signaling molecules, either at basal levels or in response to a peripheral LPS challenge. These data suggest that the repopulated microglial tissue relies on local cues for immune signaling and appears to inherit the inflammatory‐related expression properties of the cells they replace. Similar observations have been made in a study accessing the effects of microglial repopulation in the basal ganglia, where repopulated microglia exhibited similar cell densities and tissue coverage to those of their control counterparts, suggesting local extrinsic factors influence microglial anatomical and functional properties (De Biase et al., 2017).

While the current study revealed the ability to renew the entire microglial tissue in the aged brain, the effects of this process on neurons were among the most profound findings. Acute microglial replacement reversed age‐induced changes in many neuronal and synaptic‐related genes, in that gene expression patterns in 24‐month‐old mice resembled those observed in 4‐month‐old mice. In a specific way, genes associated with either actin cytoskeleton remodeling or synaptic vesicle release were downregulated with aging and subsequently restored to young adult levels with microglial replacement. Several of these genes are associated with microtubule (*Map1a*, *Map1b) *and cytoskeleton dynamics (*Prepl; *Morawski et al., 2013), and neurite (*Ttll7, Nfasc;* Ikegami et al., 2006; Wang & Bixby, 1999) as well as axon/dendrite outgrowth (*Clasp2*; Beffert et al., 2012). In addition, several genes play a role in endocytosis (*Cltc)* and microtubule transport (*Kif1a*, *Kif1b*, *Kif21a, Dync1h1, and Dnm3)*—all important in synaptic vesicle release. In accordance with the restoration of gene expression changes, microglial elimination and repopulation restored age‐related reductions in neuronal complexities to those found in the young brain, while also modulating dendritic spine densities. Crucially, actin cytoskeleton remodeling is highly associated with synaptic plasticity and memory (Lamprecht, 2014), as is synaptic vesicle release (Alabi & Tsien, 2012), and we found that microglial replacement fully restores LTP, the synaptic mechanism underlying memory formation, in 24‐month‐old mice to levels comparable to 4‐month‐old mice.

Intermediate effects on gene expression were observed in aged microglia‐depleted mice, which at the RNA level, exhibited expression profiles between those identified in aged and young mice. In addition, microglia‐eliminated mice displayed increases in both dendritic spine densities and neurogenesis in aged mice, suggesting that the elimination of microglia has profound effects on the aged brain, likely priming the brain for further beneficial effects of subsequent microglial repopulation. In support of this, our results share many commonalities with the appearance of microglia during brain development. Neuronal dendritic spine densities and connections are elevated during brain development. These synapses are subsequently pruned and the neuronal circuits sculpted/refined by the appearance and proliferation of microglia during key critical periods (Brown & Neher, 2014; Kettenmann et al., 2013; Paolicelli et al., 2011). Evidence for the importance of microglia‐mediated synaptic pruning and refining during development comes from observations that transgenic mice with perturbed microglial function have impaired cognitive function and synaptic plasticity (Rogers et al., 2011), decreased synaptic pruning, and display autistic‐like phenotypes (Zhan et al., 2014). Microglia numbers are elevated during the period of synaptic pruning in development, and following this, microglia numbers then fall to those found in the adult mouse brain (Nikodemova et al., 2015). Microglia during development also control neuronal survival and proliferation (Aarum, Sandberg, Haeberlein, & Persson, 2003; Ueno et al., 2013). Our results show that the elimination and reintroduction of microglia to the aged brain also have marked and parallel effects on neurogenesis, neuronal morphologies/circuitries, and synaptic and behavioral function. Whether these beneficial effects persist for longer periods than the 28 days investigated here is unknown and requires further investigation.

While the long‐term elimination of microglia in humans is not currently feasible (e.g., the implications of microglial absence in the face of CNS infection or injury remain unclear), data from a clinical trial indicate that microglia are effectively eliminated from humans with short‐term administration of a CSF1R inhibitor (Butowski et al., 2016), and moreover, CSF1R inhibitor‐dependent microglial elimination and repopulation are observed in nonhuman primates (Hillmer et al., 2017). Thus, short‐term CSF1R inhibitor administration, followed by drug withdrawal, appears feasible in humans using existing compounds, allowing the possibility of microglial replacement in humans. As such, microglial replacement may represent a novel approach in many conditions, notably in aging populations, conferring beneficial effects by broadly manipulating neuronal structure and function through microglia, as detailed in this study.

## 4 EXPERIMENTAL PROCEDURES

### 4.1 Experimental Design

All rodent experiments were performed in accordance with animal protocols approved by the Institutional Animal Care and Use Committee at the University of California, Irvine (UCI).

*Mice:* Male C57BL/6 young (~3 months) mice were obtained from The Jackson Laboratory (Jax). Aged (22 months) mice were obtained from the aged rodent colony of the National Institutes on Aging (originally taken from the C57BL/6 Jax colony).

*Treatments:* To eliminate microglia, PLX5622 was administered for 7 days (Supporting Information Figure S1) or 14 days (all other figures). To stimulate microglial repopulation, PLX5622 was withdrawn at the indicated time point(s) for each assay, resulting in microglial repopulation ranging from 3 to 28 days. For LTP analyses, recordings began after 28 days repopulation, thus mice were sacrificed 4–8 weeks after inhibitor removal. Experimental groups include young control (denoted as Con in figures), young microglial‐eliminated (Elim), young microglial‐repopulated (Repop), aged control (Aged Con), aged microglial‐eliminated (Aged Elim), and aged microglial‐repopulated (Aged Repop).

All detailed methods are available in the Supporting Information Appendix S1.
