# Supplementary figures and images for: Replacement of microglia in the aged brain reverses cognitive, synaptic, and neuronal deficits in mice
Source: Aging Cell. 2018 Oct 2;17(6):e12832. doi: 10.1111/acel.12832 (PMC6260908; doi:10.1111/acel.12832)

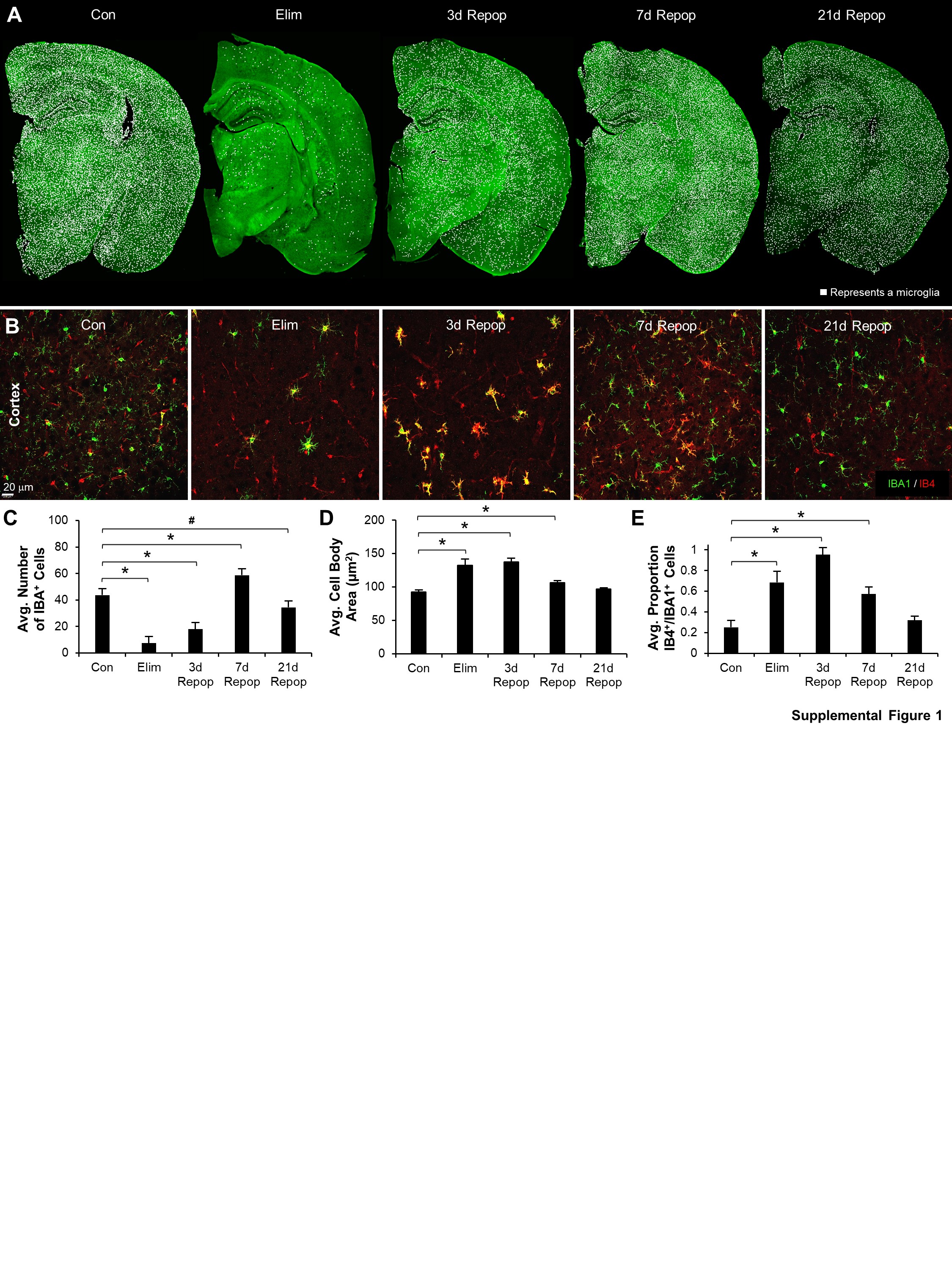

Supplement: Supplementary file 1 [file ACEL-17-e12832-s001.jpg]

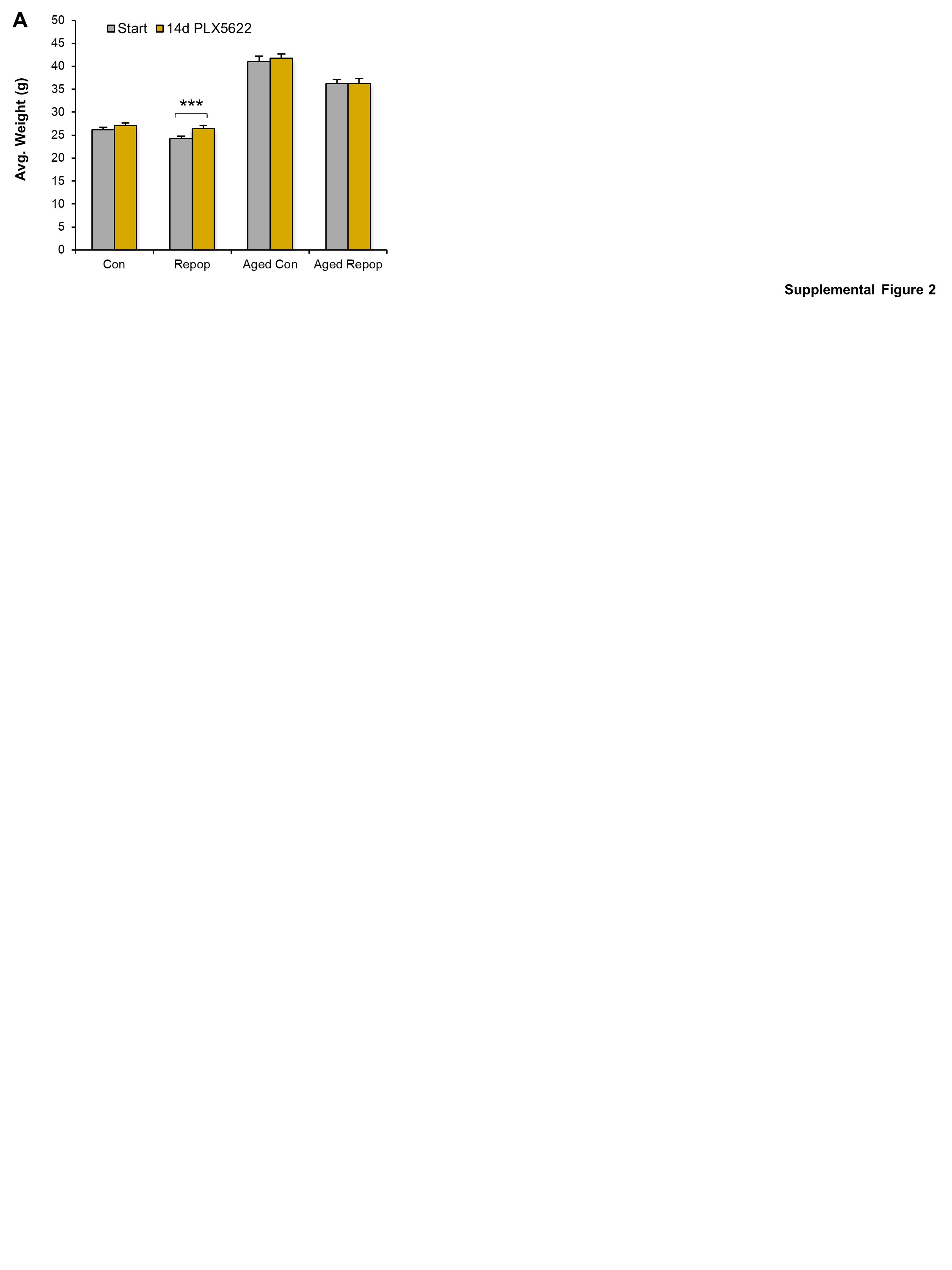

Supplement: Supplementary file 2 [file ACEL-17-e12832-s002.jpg]
