# Supplementary material for: Replacement of microglia in the aged brain reverses cognitive, synaptic, and neuronal deficits in mice
Source: Aging Cell. 2018 Oct 2;17(6):e12832. doi: 10.1111/acel.12832 (PMC6260908; doi:10.1111/acel.12832)
Supplement: Supplementary file 3 [file ACEL-17-e12832-s003.docx]

|  |  |  | 1Young Con 0h | 2Young Con 0h | 3Young Con 0h |
| --- | --- | --- | --- | --- | --- |
| Endogenous | Abcb10 | NM_019552.2 | 388.56 | 392.02 | 341.21 |
| Endogenous | Abcb1a | NM_011076.1 | 866.16 | 834.79 | 934.6 |
| Endogenous | Abcf1 | NM_013854.1 | 516.9 | 516.25 | 508.87 |
| Endogenous | Abl1 | NM_009594.3 | 170.07 | 184.17 | 180.44 |
| Endogenous | Adal | NM_029475.1 | 396.69 | 399.18 | 437.8 |
| Endogenous | Ahr | NM_013464.4 | 105.91 | 142.76 | 157.67 |
| Endogenous | Aicda | NM_009645.2 | 1 | 1 | 1 |
| Endogenous | Aire | NM_009646.1 | 1 | 1 | 1 |
| Endogenous | App | NM_007471.2 | 37920.23 | 36508.88 | 36581.45 |
| Endogenous | Arhgdib | NM_007486.4 | 303.28 | 303.62 | 268.76 |
| Endogenous | Atg16l1 | NM_029846.3 | 1437.97 | 1501.33 | 1460.37 |
| Endogenous | Atm | NM_007499.1 | 110.78 | 137.19 | 132.83 |
| Endogenous | B2m | NM_009735.3 | 4643.04 | 4449.42 | 4508.73 |
| Endogenous | Batf | NM_016767.2 | 1 | 1 | 1 |
| Endogenous | Batf3 | NM_030060.2 | 1.94 | 14.55 | 10.01 |
| Endogenous | Bax | NM_007527.3 | 404 | 398.39 | 397.78 |
| Endogenous | Bcap31 | NM_012060.4 | 1775.86 | 1900.3 | 1830.89 |
| Endogenous | Bcl2 | NM_009741.3 | 157.08 | 212.04 | 194.24 |
| Endogenous | Bcl3 | NM_033601.3 | 1 | 1 | 1 |
| Endogenous | Bcl6 | NM_009744.3 | 353.64 | 401.57 | 349.49 |
| Endogenous | Bid | NM_007544.3 | 205.81 | 209.65 | 228.74 |
| Endogenous | Blnk | NM_008528.4 | 83.16 | 109.31 | 101.78 |
| Endogenous | Bst1 | NM_009763.3 | 1 | 1 | 1 |
| Endogenous | Bst2 | NM_198095.2 | 60.42 | 68.7 | 64.52 |
| Endogenous | Btk | NM_013482.2 | 6 | 12.16 | 14.15 |
| Endogenous | Btla | NM_177584.3 | 23.06 | 18.53 | 14.84 |
| Endogenous | Btnl1 | NM_001111094.1 | 1 | 1 | 1 |
| Endogenous | Btnl2 | NM_079835.2 | 1 | 1 | 1 |
| Endogenous | C1qa | NM_007572.2 | 693.15 | 697.82 | 679.3 |
| Endogenous | C1qb | NM_009777.2 | 1107.39 | 1203.5 | 1186.44 |
| Endogenous | C1qbp | NM_007573.2 | 1818.91 | 1800.76 | 1869.53 |
| Endogenous | C1ra | NM_023143.3 | 23.06 | 34.46 | 19.67 |
| Endogenous | C1s | NM_144938.2 | 34.43 | 40.83 | 41.75 |
| Endogenous | C2 | NM_013484.2 | 53.92 | 49.59 | 28.64 |
| Endogenous | C3 | NM_009778.2 | 31.99 | 24.1 | 21.05 |
| Endogenous | C4a | NM_011413.2 | 332.52 | 309.2 | 308.78 |
| Endogenous | C4bp | NM_007576.3 | 1 | 1 | 1 |
| Endogenous | C6 | NM_016704.2 | 1 | 1 | 1 |
| Endogenous | C7 | XM_356827.6 | 2.75 | 1.01 | 1 |
| Endogenous | C8a | NM_146148.1 | 1 | 1 | 1 |
| Endogenous | C8b | NM_133882.2 | 4.38 | 1.81 | 1 |
| Endogenous | C8g | NM_027062.1 | 2.75 | 8.97 | 7.94 |
| Endogenous | C9 | NM_013485.1 | 1 | 1 | 1 |
| Endogenous | Camp | NM_009921.2 | 1 | 3.4 | 1 |
| Endogenous | Card9 | NM_001037747.1 | 40.93 | 27.29 | 34.85 |
| Endogenous | Casp1 | NM_009807.2 | 49.05 | 47.2 | 45.2 |
| Endogenous | Casp2 | NM_007610.1 | 205 | 259.03 | 212.87 |

| Endogenous | Casp3 | NM_009810.2 | 339.83 | 375.29 | 371.57 |
| --- | --- | --- | --- | --- | --- |
| Endogenous | Casp8 | NM_009812.2 | 58.8 | 57.55 | 50.03 |
| Endogenous | Ccbp2 | NM_021609.3 | 6 | 17.73 | 20.36 |
| Endogenous | Ccl11 | NM_011330.3 | 1 | 1.01 | 1 |
| Endogenous | Ccl12 | NM_011331.2 | 1 | 3.4 | 1 |
| Endogenous | Ccl19 | NM_011888.2 | 48.24 | 57.55 | 55.55 |
| Endogenous | Ccl2 | NM_011333.3 | 1 | 1.01 | 1 |
| Endogenous | Ccl20 | NM_016960.1 | 1 | 1 | 1 |
| Endogenous | Ccl22 | NM_009137.2 | 3.57 | 4.99 | 4.49 |
| Endogenous | Ccl24 | NM_019577.4 | 1 | 1 | 1 |
| Endogenous | Ccl25 | NM_009138.3 | 141.65 | 162.67 | 150.08 |
| Endogenous | Ccl26 | NM_001013412.2 | 1 | 1 | 1 |
| Endogenous | Ccl3 | NM_011337.1 | 1 | 1 | 1.73 |
| Endogenous | Ccl4 | NM_013652.1 | 1 | 1 | 1 |
| Endogenous | Ccl5 | NM_013653.1 | 1 | 1 | 1 |
| Endogenous | Ccl6 | NM_009139.2 | 1 | 11.36 | 6.56 |
| Endogenous | Ccl7 | NM_013654.2 | 192 | 180.19 | 187.34 |
| Endogenous | Ccl8 | NM_021443.2 | 1 | 1 | 1 |
| Endogenous | Ccl9 | NM_011338.2 | 38.49 | 35.25 | 40.37 |
| Endogenous | Ccr10 | NM_007721.4 | 1 | 1 | 1 |
| Endogenous | Ccr2 | NM_009915.2 | 1.94 | 1.81 | 1 |
| Endogenous | Ccr3 | NM_009914.4 | 1 | 1 | 1 |
| Endogenous | Ccr4 | NM_009916.2 | 1 | 1 | 1 |
| Endogenous | Ccr5 | NM_009917.5 | 28.75 | 30.47 | 44.51 |
| Endogenous | Ccr6 | NM_001190333.1 | 1 | 1 | 1 |
| Endogenous | Ccr7 | NM_007719.2 | 1 | 1 | 1 |
| Endogenous | Ccr8 | NM_007720.2 | 1 | 1 | 1 |
| Endogenous | Ccr9 | NM_009913.6 | 1.94 | 3.4 | 1.04 |
| Endogenous | Ccrl1 | NM_145700.2 | 1 | 7.38 | 1 |
| Endogenous | Ccrl2 | NM_017466.4 | 12.5 | 28.88 | 13.46 |
| Endogenous | Cd109 | NM_153098.3 | 44.99 | 36.05 | 31.4 |
| Endogenous | Cd14 | NM_009841.3 | 18.19 | 16.94 | 31.4 |
| Endogenous | Cd160 | NM_001163496.1 | 1 | 1 | 1 |
| Endogenous | Cd163 | NM_053094.2 | 29.56 | 23.31 | 24.5 |
| Endogenous | Cd164 | NM_016898.2 | 1921.25 | 1958.44 | 1852.97 |
| Endogenous | Cd19 | NM_009844.2 | 1 | 1 | 1 |
| Endogenous | Cd1d1 | NM_007639.3 | 50.68 | 60.74 | 59 |
| Endogenous | Cd2 | NM_013486.2 | 1 | 1 | 1 |
| Endogenous | Cd209g | NM_027343.3 | 1 | 1 | 1 |
| Endogenous | Cd22 | NM_001043317.2 | 1 | 1 | 1 |
| Endogenous | Cd226 | NM_001039149.1 | 1 | 1 | 1 |
| Endogenous | Cd244 | NM_018729.2 | 1 | 1 | 1 |
| Endogenous | Cd247 | NM_001113391.2 | 1 | 2.6 | 1 |
| Endogenous | Cd24a | NM_009846.2 | 260.23 | 258.23 | 228.74 |
| Endogenous | Cd27 | NM_001042564.1 | 1 | 1 | 1 |
| Endogenous | Cd274 | NM_021893.2 | 26.31 | 51.98 | 37.61 |
| Endogenous | Cd28 | NM_007642.4 | 1 | 1.01 | 1 |
| Endogenous | Cd34 | NM_001111059.1 | 1428.22 | 1444 | 1579.04 |

| Endogenous | Cd36 | NM_007643.3 | 16.56 | 7.38 | 20.36 |
| --- | --- | --- | --- | --- | --- |
| Endogenous | Cd3d | NM_013487.2 | 2.75 | 4.2 | 1 |
| Endogenous | Cd3e | NM_007648.4 | 1 | 1 | 1 |
| Endogenous | Cd3eap | NM_145822.2 | 100.22 | 106.92 | 119.03 |
| Endogenous | Cd4 | NM_013488.2 | 68.54 | 126.04 | 128.69 |
| Endogenous | Cd40 | NM_011611.2 | 10.88 | 14.55 | 7.25 |
| Endogenous | Cd40lg | NM_011616.2 | 1 | 1 | 1 |
| Endogenous | Cd44 | NM_009851.2 | 183.88 | 187.36 | 138.35 |
| Endogenous | Cd46 | NM_010778.3 | 1 | 1 | 1 |
| Endogenous | Cd48 | NM_007649.4 | 17.37 | 11.36 | 16.91 |
| Endogenous | Cd5 | NM_007650.3 | 1 | 1 | 2.42 |
| Endogenous | Cd53 | NM_007651.3 | 2.75 | 5.79 | 7.94 |
| Endogenous | Cd55 | NM_010016.2 | 117.28 | 118.07 | 105.23 |
| Endogenous | Cd59b | NM_181858.1 | 8.44 | 11.36 | 1.73 |
| Endogenous | Cd6 | NM_001037801.2 | 11.69 | 3.4 | 18.29 |
| Endogenous | Cd69 | NM_001033122.3 | 1 | 1 | 1 |
| Endogenous | Cd7 | NM_009854.1 | 1 | 4.99 | 13.46 |
| Endogenous | Cd74 | NM_001042605.1 | 332.52 | 282.12 | 288.08 |
| Endogenous | Cd79a | NM_007655.3 | 1 | 1 | 1 |
| Endogenous | Cd79b | NM_008339.2 | 4.38 | 1 | 1 |
| Endogenous | Cd80 | NM_009855.2 | 2.75 | 16.14 | 7.94 |
| Endogenous | Cd81 | NM_133655.2 | 15528.59 | 15107.77 | 14617.75 |
| Endogenous | Cd82 | NM_001271430.1 | 389.38 | 356.98 | 343.28 |
| Endogenous | Cd83 | NM_009856.2 | 952.25 | 784.62 | 804.19 |
| Endogenous | Cd86 | NM_019388.3 | 31.18 | 32.86 | 23.81 |
| Endogenous | Cd8a | NM_001081110.2 | 1 | 1 | 1 |
| Endogenous | Cd8b1 | NM_009858.2 | 1 | 1 | 1 |
| Endogenous | Cd9 | NM_007657.3 | 1480.21 | 1294.28 | 1374.12 |
| Endogenous | Cd96 | NM_032465.2 | 1 | 1 | 1 |
| Endogenous | Cd97 | NM_011925.1 | 168.45 | 153.91 | 135.59 |
| Endogenous | Cd99 | NM_025584.2 | 188.76 | 204.08 | 205.97 |
| Endogenous | Cdh5 | NM_009868.3 | 109.97 | 146.74 | 140.42 |
| Endogenous | Cdkn1a | NM_007669.4 | 269.98 | 350.61 | 358.46 |
| Endogenous | Ceacam1 | NM_001039185.1 | 4.38 | 12.16 | 5.87 |
| Endogenous | Cebpb | NM_009883.3 | 140.83 | 165.85 | 158.36 |
| Endogenous | Cfb | NM_008198.2 | 1 | 1 | 1 |
| Endogenous | Cfd | NM_013459.1 | 1 | 1 | 1 |
| Endogenous | Cfh | NM_009888.3 | 424.3 | 431.83 | 411.58 |
| Endogenous | Cfi | NM_007686.2 | 1 | 1 | 1 |
| Endogenous | Cfp | NM_008823.3 | 180.63 | 212.84 | 165.95 |
| Endogenous | Chuk | NM_001162410.1 | 858.04 | 973.35 | 886.3 |
| Endogenous | Ciita | NM_007575.2 | 1 | 1 | 1 |
| Endogenous | Cish | NM_009895.3 | 15.75 | 29.68 | 26.57 |
| Endogenous | Clec4a4 | NM_001005860.2 | 1 | 1 | 1 |
| Endogenous | Clec4e | NM_019948.2 | 1 | 1 | 1 |
| Endogenous | Clec5a | NM_001038604.1 | 35.24 | 24.1 | 25.88 |
| Endogenous | Clu | NM_013492.2 | 23232.62 | 22910.4 | 23272.27 |
| Endogenous | Cmklr1 | NM_008153.3 | 31.99 | 54.37 | 49.34 |

| Endogenous | Cr2 | NM_007758.2 | 1 | 1 | 1 |
| --- | --- | --- | --- | --- | --- |
| Endogenous | Cradd | NM_009950.2 | 176.57 | 192.13 | 185.96 |
| Endogenous | Crlf2 | NM_001164735.1 | 590.81 | 595.09 | 658.6 |
| Endogenous | Csf1 | NM_001113530.1 | 538.83 | 513.06 | 504.04 |
| Endogenous | Csf1r | NM_001037859.1 | 879.15 | 920.79 | 931.84 |
| Endogenous | Csf2 | NM_009969.4 | 1 | 1 | 1 |
| Endogenous | Csf2rb | NM_007780.4 | 1 | 4.2 | 1 |
| Endogenous | Csf3r | NM_001252651.1 | 16.56 | 25.7 | 36.23 |
| Endogenous | Ctla4 | NM_009843.3 | 1 | 1 | 1 |
| Endogenous | Ctnnb1 | NM_007614.2 | 7714.91 | 7314.69 | 7381.84 |
| Endogenous | Ctsc | NM_009982.2 | 173.32 | 161.87 | 150.08 |
| Endogenous | Ctsg | NM_007800.1 | 1 | 1 | 1 |
| Endogenous | Ctss | NM_021281.2 | 1641.84 | 1670.96 | 1783.97 |
| Endogenous | Cul9 | NM_001081335.2 | 406.43 | 405.56 | 436.42 |
| Endogenous | Cx3cl1 | NM_009142.3 | 2336.3 | 2579.59 | 2720.28 |
| Endogenous | Cx3cr1 | NM_009987.3 | 572.94 | 505.9 | 573.73 |
| Endogenous | Cxcl1 | NM_008176.1 | 1 | 3.4 | 15.53 |
| Endogenous | Cxcl10 | NM_021274.1 | 1 | 1 | 4.49 |
| Endogenous | Cxcl11 | NM_019494.1 | 1 | 1 | 1 |
| Endogenous | Cxcl12 | NM_021704.3 | 1400.61 | 1191.55 | 1216.11 |
| Endogenous | Cxcl13 | NM_018866.2 | 1 | 1 | 1 |
| Endogenous | Cxcl15 | NM_011339.2 | 1 | 1 | 1 |
| Endogenous | Cxcl3 | NM_203320.2 | 1 | 1 | 1 |
| Endogenous | Cxcl9 | NM_008599.2 | 1 | 1.81 | 9.32 |
| Endogenous | Cxcr1 | NM_178241.4 | 1 | 1 | 1 |
| Endogenous | Cxcr2 | NM_009909.3 | 1 | 1 | 1 |
| Endogenous | Cxcr3 | NM_009910.2 | 1 | 1 | 1 |
| Endogenous | Cxcr4 | NM_009911.3 | 60.42 | 48.79 | 39.68 |
| Endogenous | Cxcr5 | NM_007551.2 | 1 | 1 | 1 |
| Endogenous | Cxcr6 | NM_030712.4 | 1 | 1 | 1 |
| Endogenous | Cybb | NM_007807.2 | 21.43 | 25.7 | 12.77 |
| Endogenous | Ddx58 | NM_172689.3 | 144.89 | 141.96 | 143.18 |
| Endogenous | Defb1 | NM_007843.3 | 15.75 | 4.99 | 18.98 |
| Endogenous | Defb14 | NM_183026.2 | 1 | 1 | 1 |
| Endogenous | Dpp4 | NM_001159543.1 | 23.87 | 12.16 | 25.88 |
| Endogenous | Ebi3 | NM_015766.2 | 29.56 | 27.29 | 36.23 |
| Endogenous | Emr1 | NM_010130.1 | 133.52 | 119.67 | 137.66 |
| Endogenous | Entpd1 | NM_009848.3 | 154.64 | 186.56 | 174.23 |
| Endogenous | Eomes | NM_010136.2 | 54.74 | 93.39 | 54.17 |
| Endogenous | Ets1 | NM_001038642.1 | 339.83 | 353 | 359.15 |
| Endogenous | Fadd | NM_010175.5 | 49.05 | 29.68 | 34.16 |
| Endogenous | Fas | NM_007987.2 | 5.19 | 19.33 | 26.57 |
| Endogenous | Fasl | NM_010177.3 | 1 | 1 | 1 |
| Endogenous | Fcamr | NM_001170632.1 | 1 | 1 | 1 |
| Endogenous | Fcer1a | NM_010184.1 | 1 | 1 | 1 |
| Endogenous | Fcer1g | NM_010185.4 | 47.43 | 71.09 | 56.24 |
| Endogenous | Fcgr1 | NM_010186.5 | 54.74 | 47.99 | 56.93 |
| Endogenous | Fcgr2b | NM_001077189.1 | 157.08 | 132.41 | 140.42 |

| Endogenous | Fcgr3 | NM_010188.5 | 80.73 | 91 | 103.16 |
| --- | --- | --- | --- | --- | --- |
| Endogenous | Fcgr4 | NM_144559.1 | 1 | 1 | 1 |
| Endogenous | Fcgrt | NM_010189.3 | 325.21 | 332.29 | 324.65 |
| Endogenous | Fkbp5 | NM_010220.3 | 206.62 | 182.58 | 219.77 |
| Endogenous | Fn1 | NM_010233.1 | 664.72 | 609.42 | 655.15 |
| Endogenous | Folr4 | NM_022888.2 | 1 | 1 | 1 |
| Endogenous | Foxp3 | NM_054039.1 | 1 | 1 | 1 |
| Endogenous | Frmpd4 | NM_001033330.2 | 595.68 | 669.94 | 660.67 |
| Endogenous | Fyn | NM_008054.2 | 3860.05 | 3653.07 | 3553.79 |
| Endogenous | Gata3 | NM_008091.3 | 53.92 | 47.99 | 56.24 |
| Endogenous | Gfi1 | NM_010278.2 | 1 | 1 | 1 |
| Endogenous | Gm10499 | XM_003086920.1 | 8.44 | 12.95 | 18.98 |
| Endogenous | Gp1bb | NM_010327.2 | 84.79 | 83.03 | 101.09 |
| Endogenous | Gpi1 | NM_008155.3 | 192 | 188.15 | 203.21 |
| Endogenous | Gpr183 | NM_183031.2 | 2.75 | 9.77 | 1.04 |
| Endogenous | Gpr44 | NM_009962.2 | 1 | 1 | 1 |
| Endogenous | Gzma | NM_010370.2 | 1 | 1 | 1 |
| Endogenous | Gzmb | NM_013542.2 | 1 | 1 | 1 |
| Endogenous | H2-Aa | NM_010378.2 | 166.01 | 149.13 | 125.93 |
| Endogenous | H2-Ab1 | NM_207105.2 | 71.79 | 73.48 | 58.31 |
| Endogenous | H2-DMa | NM_010386.3 | 236.68 | 231.95 | 247.37 |
| Endogenous | H2-DMb2 | NM_010388.4 | 1 | 4.99 | 5.87 |
| Endogenous | H2-Ea-ps | NM_010381.2 | 1 | 1 | 1 |
| Endogenous | H2-Eb1 | NM_010382.2 | 49.05 | 61.53 | 41.75 |
| Endogenous | H2-K1 | NM_001001892.2 | 244.8 | 243.9 | 291.53 |
| Endogenous | H2-Ob | NM_010389.3 | 1 | 4.99 | 1 |
| Endogenous | H2-Q10 | NM_010391.4 | 1 | 1 | 1 |
| Endogenous | H60a | NM_010400.2 | 1 | 1 | 1 |
| Endogenous | Hamp | NM_032541.1 | 1 | 1 | 1 |
| Endogenous | Hc | NM_010406.1 | 1 | 1 | 1 |
| Endogenous | Hcst | NM_011827.3 | 1 | 1 | 1 |
| Endogenous | Hfe | NM_010424.4 | 235.86 | 206.47 | 192.86 |
| Endogenous | Hif1a | NM_010431.1 | 1252.78 | 1268 | 1049.13 |
| Endogenous | Hlx | NM_008250.2 | 4.38 | 12.95 | 1.04 |
| Endogenous | Icam1 | NM_010493.2 | 18.19 | 25.7 | 27.95 |
| Endogenous | Icam2 | NM_010494.1 | 96.97 | 117.28 | 104.54 |
| Endogenous | Icam4 | NM_023892.2 | 1 | 9.77 | 21.05 |
| Endogenous | Icam5 | NM_008319.2 | 616.8 | 611.81 | 651.01 |
| Endogenous | Icos | NM_017480.1 | 1 | 1 | 1 |
| Endogenous | Icosl | NM_015790.3 | 150.58 | 136.39 | 136.28 |
| Endogenous | Ifi204 | NM_008329.2 | 1 | 1 | 1 |
| Endogenous | Ifi35 | NM_027320.4 | 74.23 | 75.07 | 61.76 |
| Endogenous | Ifih1 | NM_027835.2 | 149.77 | 123.65 | 155.6 |
| Endogenous | Ifit2 | NM_008332.2 | 256.98 | 258.23 | 272.21 |
| Endogenous | Ifitm1 | NM_001112715.1 | 157.89 | 171.43 | 170.78 |
| Endogenous | Ifna1 | NM_010502.2 | 1 | 1 | 1 |
| Endogenous | Ifna2 | NM_010503.2 | 1 | 1 | 1 |
| Endogenous | Ifnar1 | NM_010508.1 | 1567.11 | 1580.17 | 1605.95 |

| Endogenous | Ifnar2 | NM_001110498.1 | 399.94 | 410.33 | 381.92 |
| --- | --- | --- | --- | --- | --- |
| Endogenous | Ifnb1 | NM_010510.1 | 1 | 1 | 1 |
| Endogenous | Ifng | NM_008337.1 | 1 | 1 | 1 |
| Endogenous | Ifngr1 | NM_010511.2 | 469.79 | 465.28 | 419.86 |
| Endogenous | Ifngr2 | NM_008338.3 | 918.14 | 877.79 | 870.43 |
| Endogenous | Igf2r | NM_010515.1 | 356.08 | 394.41 | 329.48 |
| Endogenous | Ikbkap | NM_026079.3 | 644.42 | 722.5 | 589.6 |
| Endogenous | Ikbkb | NM_010546.2 | 377.19 | 350.61 | 369.5 |
| Endogenous | Ikbke | NM_019777.3 | 25.5 | 37.64 | 21.05 |
| Endogenous | Ikbkg | NM_178590.2 | 348.76 | 344.24 | 343.28 |
| Endogenous | Ikzf1 | NM_001025597.1 | 5.19 | 3.4 | 19.67 |
| Endogenous | Ikzf2 | NM_011770.4 | 149.77 | 144.35 | 137.66 |
| Endogenous | Ikzf3 | NM_011771.1 | 1 | 1 | 1 |
| Endogenous | Ikzf4 | NM_011772.2 | 255.36 | 294.07 | 293.6 |
| Endogenous | Il10 | NM_010548.1 | 1 | 1 | 1 |
| Endogenous | Il10ra | NM_008348.2 | 85.6 | 87.02 | 98.33 |
| Endogenous | Il10rb | NM_008349.5 | 272.42 | 298.84 | 259.1 |
| Endogenous | Il11ra1 | NM_010549.3 | 13.31 | 26.49 | 16.91 |
| Endogenous | Il12a | NM_008351.1 | 16.56 | 21.71 | 30.71 |
| Endogenous | Il12b | NM_008352.1 | 1 | 1 | 1 |
| Endogenous | Il12rb1 | NM_008353.2 | 1 | 1 | 1 |
| Endogenous | Il12rb2 | NM_008354.3 | 1 | 1 | 1 |
| Endogenous | Il13 | NM_008355.2 | 1 | 1 | 1 |
| Endogenous | Il13ra1 | NM_133990.4 | 179.01 | 195.32 | 205.28 |
| Endogenous | Il15 | NM_008357.2 | 1 | 4.99 | 1 |
| Endogenous | Il15ra | NM_008358.2 | 38.49 | 20.92 | 29.33 |
| Endogenous | Il16 | NM_010551.3 | 312.21 | 360.96 | 271.52 |
| Endogenous | Il17a | NM_010552.3 | 1 | 1 | 1 |
| Endogenous | Il17b | NM_019508.1 | 1 | 1 | 1 |
| Endogenous | Il17f | NM_145856.2 | 1.94 | 1 | 1.04 |
| Endogenous | Il17ra | NM_008359.1 | 181.44 | 207.26 | 221.15 |
| Endogenous | Il17rb | NM_019583.3 | 40.93 | 29.68 | 32.09 |
| Endogenous | Il17re | NM_001034029.1 | 5.19 | 8.18 | 1 |
| Endogenous | Il18 | NM_008360.1 | 1140.69 | 1155.72 | 1088.46 |
| Endogenous | Il18r1 | NM_001161842.1 | 1 | 1 | 1 |
| Endogenous | Il18rap | NM_010553.2 | 1 | 1 | 1 |
| Endogenous | Il19 | NM_001009940.1 | 1 | 1 | 1 |
| Endogenous | Il1a | NM_010554.4 | 1 | 8.18 | 1.73 |
| Endogenous | Il1b | NM_008361.3 | 1 | 1 | 1 |
| Endogenous | Il1r1 | NM_001123382.1 | 133.52 | 118.07 | 119.72 |
| Endogenous | Il1r2 | NM_010555.4 | 3.57 | 8.97 | 9.32 |
| Endogenous | Il1rap | NM_134103.2 | 889.71 | 778.25 | 754.51 |
| Endogenous | Il1rl1 | NM_001025602.2 | 1 | 2.6 | 1 |
| Endogenous | Il1rl2 | NM_133193.3 | 1 | 1 | 1 |
| Endogenous | Il1rn | NM_031167.5 | 1 | 1 | 1 |
| Endogenous | Il2 | NM_008366.2 | 1 | 1 | 1 |
| Endogenous | Il20 | NM_021380.1 | 1 | 1 | 1 |
| Endogenous | Il21 | NM_021782.2 | 1.13 | 1 | 1.04 |

| Endogenous | Il21r | NM_021887.1 | 1 | 1.81 | 1.04 |
| --- | --- | --- | --- | --- | --- |
| Endogenous | Il22 | NM_016971.1 | 229.37 | 273.36 | 170.78 |
| Endogenous | Il22ra2 | NM_178258.5 | 1 | 1 | 1 |
| Endogenous | Il23a | NM_031252.1 | 1 | 1 | 1 |
| Endogenous | Il23r | NM_144548.1 | 1 | 1 | 1 |
| Endogenous | Il25 | NM_080729.2 | 1 | 1 | 1 |
| Endogenous | Il27 | NM_145636.1 | 1 | 1 | 1 |
| Endogenous | Il27ra | NM_016671.3 | 1 | 1 | 1 |
| Endogenous | Il28a | NM_001024673.2 | 1 | 1 | 1 |
| Endogenous | Il2ra | NM_008367.2 | 1 | 1.01 | 1 |
| Endogenous | Il2rb | NM_008368.3 | 1 | 3.4 | 3.8 |
| Endogenous | Il2rg | NM_013563.3 | 4.38 | 4.99 | 9.32 |
| Endogenous | Il3 | NM_010556.4 | 1 | 1 | 1 |
| Endogenous | Il33 | NM_133775.1 | 1408.73 | 1266.41 | 1197.48 |
| Endogenous | Il4 | NM_021283.1 | 1 | 1 | 1 |
| Endogenous | Il4ra | NM_001008700.3 | 141.65 | 142.76 | 119.03 |
| Endogenous | Il5 | NM_010558.1 | 1 | 1 | 1 |
| Endogenous | Il6 | NM_031168.1 | 1 | 1 | 1 |
| Endogenous | Il6ra | NM_010559.2 | 88.04 | 63.92 | 90.05 |
| Endogenous | Il6st | NM_010560.2 | 827.17 | 830.81 | 744.16 |
| Endogenous | Il7 | NM_008371.2 | 1.13 | 1 | 1 |
| Endogenous | Il7r | NM_008372.3 | 1 | 4.2 | 2.42 |
| Endogenous | Il9 | NM_008373.1 | 1 | 1 | 1 |
| Endogenous | Ilf3 | NM_010561.2 | 642.79 | 694.63 | 649.63 |
| Endogenous | Irak1 | NM_008363.2 | 633.86 | 673.93 | 648.94 |
| Endogenous | Irak2 | NM_001113553.1 | 487.66 | 484.39 | 477.13 |
| Endogenous | Irak3 | NM_028679.3 | 45.8 | 44.01 | 36.92 |
| Endogenous | Irak4 | NM_029926.5 | 58.8 | 73.48 | 84.53 |
| Endogenous | Irf1 | NM_008390.1 | 112.41 | 111.7 | 96.95 |
| Endogenous | Irf3 | NM_016849.3 | 11.69 | 15.34 | 7.94 |
| Endogenous | Irf4 | NM_013674.1 | 1 | 1 | 1 |
| Endogenous | Irf5 | NM_012057.3 | 42.55 | 32.86 | 51.41 |
| Endogenous | Irf7 | NM_016850.2 | 6 | 13.75 | 15.53 |
| Endogenous | Irf8 | NM_008320.3 | 33.62 | 38.44 | 34.16 |
| Endogenous | Irgm1 | NM_008326.1 | 235.05 | 271.77 | 268.76 |
| Endogenous | Itga2b | NM_010575.2 | 1 | 1 | 1 |
| Endogenous | Itga4 | NM_010576.3 | 180.63 | 196.91 | 203.9 |
| Endogenous | Itga5 | NM_010577.3 | 14.12 | 24.9 | 17.6 |
| Endogenous | Itga6 | NM_008397.3 | 649.29 | 623.76 | 635.14 |
| Endogenous | Itgal | NM_008400.2 | 1 | 1.81 | 1 |
| Endogenous | Itgam | NM_001082960.1 | 161.14 | 164.26 | 185.27 |
| Endogenous | Itgax | NM_021334.2 | 1.13 | 1.81 | 1 |
| Endogenous | Itgb1 | NM_010578.1 | 1515.13 | 1592.12 | 1585.94 |
| Endogenous | Itgb2 | NM_008404.4 | 19.81 | 8.97 | 15.53 |
| Endogenous | Itln1 | NM_010584.3 | 1 | 1 | 1 |
| Endogenous | Jak1 | NM_146145.2 | 3465.3 | 3551.93 | 3555.17 |
| Endogenous | Jak2 | NM_001048177.1 | 1061.91 | 1141.38 | 1063.62 |
| Endogenous | Jak3 | NM_010589.5 | 49.86 | 44.01 | 45.2 |

| Endogenous | Kir3dl1 | NM_177749.3 | 1 | 1 | 1 |
| --- | --- | --- | --- | --- | --- |
| Endogenous | Kir3dl2 | NM_177748.2 | 1 | 1 | 1 |
| Endogenous | Kit | NM_001122733.1 | 357.7 | 388.04 | 347.42 |
| Endogenous | Klra1 | NM_016659.3 | 1 | 1 | 1 |
| Endogenous | Klra21 | NM_053151.1 | 1 | 1 | 1 |
| Endogenous | Klra4 | NM_010649.3 | 1 | 1 | 1 |
| Endogenous | Klra5 | NM_008463.2 | 1 | 1 | 1 |
| Endogenous | Klra6 | NM_008464.2 | 1 | 1 | 1 |
| Endogenous | Klra7 | NM_001110323.1 | 1 | 1 | 1 |
| Endogenous | Klra8 | NM_010650.3 | 1 | 1 | 1 |
| Endogenous | Klrb1 | NM_001099918.1 | 1 | 1 | 1 |
| Endogenous | Klrc1 | NM_001136068.1 | 1 | 1 | 1 |
| Endogenous | Klrc2 | NM_001098669.1 | 1 | 1 | 1 |
| Endogenous | Klrc3 | NM_021378.1 | 1 | 1 | 1 |
| Endogenous | Klrd1 | NM_010654.2 | 1 | 1 | 1 |
| Endogenous | Klrk1 | NM_001083322.1 | 1 | 1 | 1 |
| Endogenous | Lair1 | NM_001113474.1 | 82.35 | 104.54 | 90.05 |
| Endogenous | Lck | NM_010693.2 | 21.43 | 18.53 | 21.05 |
| Endogenous | Lcp2 | NM_010696.3 | 31.99 | 24.1 | 29.33 |
| Endogenous | Lef1 | NM_010703.3 | 205.81 | 192.13 | 183.89 |
| Endogenous | Lif | NM_008501.2 | 1.13 | 1 | 3.8 |
| Endogenous | Lilra5 | NM_001081239.2 | 1 | 1 | 1 |
| Endogenous | Lilra6 | NM_011090.2 | 1 | 1 | 1 |
| Endogenous | Lilrb3 | NM_011095.2 | 1 | 1 | 1 |
| Endogenous | Lilrb4 | NM_013532.2 | 1 | 15.34 | 8.63 |
| Endogenous | Litaf | NM_019980.1 | 298.41 | 286.1 | 257.72 |
| Endogenous | Lta | NM_010735.1 | 1 | 1 | 1 |
| Endogenous | Ltb | NM_008518.2 | 1.94 | 12.16 | 10.01 |
| Endogenous | Ltb4r1 | NM_008519.2 | 1 | 1 | 1 |
| Endogenous | Ltb4r2 | NM_020490.2 | 1 | 1 | 1 |
| Endogenous | Ltbr | NM_010736.3 | 78.29 | 85.42 | 65.9 |
| Endogenous | Ltf | NM_008522.3 | 1 | 1 | 1 |
| Endogenous | Ly86 | NM_010745.2 | 312.21 | 321.14 | 328.1 |
| Endogenous | Ly96 | NM_016923.1 | 46.61 | 70.29 | 72.8 |
| Endogenous | Maf | NM_001025577.2 | 186.32 | 227.97 | 210.11 |
| Endogenous | Map4k1 | NM_008279.2 | 1.13 | 4.99 | 3.8 |
| Endogenous | Map4k2 | NM_009006.2 | 603.81 | 557.66 | 496.45 |
| Endogenous | Map4k4 | NM_008696.2 | 1227.6 | 1232.96 | 1087.77 |
| Endogenous | Mapk1 | NM_011949.3 | 7848.11 | 8018.66 | 8603.12 |
| Endogenous | Mapk11 | NM_011161.5 | 114.03 | 116.48 | 105.23 |
| Endogenous | Mapk14 | NM_011951.2 | 945.76 | 993.26 | 965.65 |
| Endogenous | Mapkapk2 | NM_008551.1 | 438.92 | 393.61 | 411.58 |
| Endogenous | Marco | NM_010766.2 | 1 | 1 | 1 |
| Endogenous | Masp1 | NM_008555.2 | 73.42 | 111.7 | 116.96 |
| Endogenous | Masp2 | NM_010767.3 | 1 | 1 | 1 |
| Endogenous | Mbl2 | NM_010776.1 | 1 | 1 | 1 |
| Endogenous | Mbp | NM_010777.3 | 10028.96 | 9442.53 | 8743.88 |
| Endogenous | Mif | NM_010798.2 | 5779.36 | 5841.44 | 6010.84 |

| Endogenous | Mme | NM_008604.3 | 208.25 | 252.66 | 270.83 |
| --- | --- | --- | --- | --- | --- |
| Endogenous | Mr1 | NM_008209.4 | 166.82 | 141.17 | 129.38 |
| Endogenous | Ms4a1 | NM_007641.5 | 1.13 | 1 | 1.04 |
| Endogenous | Msr1 | NM_001113326.1 | 1 | 1.01 | 1 |
| Endogenous | Muc1 | NM_013605.1 | 10.88 | 16.14 | 9.32 |
| Endogenous | Mx1 | NM_010846.1 | 265.1 | 282.92 | 237.02 |
| Endogenous | Myd88 | NM_010851.2 | 74.23 | 75.87 | 83.84 |
| Endogenous | Ncam1 | NM_001113204.1 | 7083.8 | 6923.68 | 6836.75 |
| Endogenous | Ncf4 | NM_008677.2 | 5.19 | 9.77 | 7.25 |
| Endogenous | Nfatc1 | NM_016791.4 | 26.31 | 24.9 | 35.54 |
| Endogenous | Nfatc2 | NM_001037177.1 | 265.1 | 251.86 | 237.02 |
| Endogenous | Nfatc3 | NM_010901.2 | 248.86 | 271.77 | 279.8 |
| Endogenous | Nfil3 | NM_017373.3 | 243.17 | 291.68 | 288.08 |
| Endogenous | Nfkb1 | NM_008689.2 | 126.21 | 141.17 | 140.42 |
| Endogenous | Nfkb2 | NM_019408.2 | 65.3 | 63.92 | 50.72 |
| Endogenous | Nfkbia | NM_010907.2 | 314.65 | 459.71 | 448.84 |
| Endogenous | Nfkbiz | NM_030612.1 | 140.83 | 148.33 | 148.01 |
| Endogenous | Nod2 | NM_145857.2 | 1 | 7.38 | 1 |
| Endogenous | Nos2 | NM_010927.3 | 1 | 3.4 | 1.73 |
| Endogenous | Notch1 | NM_008714.2 | 388.56 | 388.83 | 351.56 |
| Endogenous | Notch2 | NM_010928.1 | 267.54 | 251.06 | 216.32 |
| Endogenous | Nox1 | NM_172203.1 | 1 | 1 | 1 |
| Endogenous | Nox3 | NM_198958.2 | 1 | 1 | 1 |
| Endogenous | Nox4 | NM_015760.4 | 14.94 | 11.36 | 2.42 |
| Endogenous | Npc1 | NM_008720.2 | 1987.04 | 1922.6 | 1913 |
| Endogenous | Nt5e | NM_011851.3 | 103.47 | 141.17 | 138.35 |
| Endogenous | Pax5 | NM_008782.2 | 10.06 | 9.77 | 5.87 |
| Endogenous | Pdcd1 | NM_008798.1 | 1 | 1 | 1 |
| Endogenous | Pdcd1lg2 | NM_021396.2 | 1 | 1 | 1 |
| Endogenous | Pdcd2 | NM_008799.2 | 105.09 | 112.5 | 74.87 |
| Endogenous | Pdgfb | NM_011057.3 | 483.6 | 539.34 | 529.57 |
| Endogenous | Pdgfrb | NM_008809.1 | 207.44 | 210.45 | 190.79 |
| Endogenous | Pecam1 | NM_008816.2 | 485.22 | 551.29 | 517.84 |
| Endogenous | Phlpp1 | NM_133821.3 | 2657.94 | 2571.63 | 2431.18 |
| Endogenous | Phlpp2 | NM_001122594.2 | 1029.42 | 1054.58 | 1057.41 |
| Endogenous | Pigr | NM_011082.3 | 1 | 1 | 1 |
| Endogenous | Pla2g2a | NM_001082531.1 | 1 | 1 | 1 |
| Endogenous | Pla2g2e | NM_012044.2 | 1 | 1 | 1 |
| Endogenous | Plau | NM_008873.2 | 33.62 | 32.07 | 32.09 |
| Endogenous | Plaur | NM_011113.3 | 21.43 | 22.51 | 32.78 |
| Endogenous | Pml | NM_008884.2 | 284.6 | 308.4 | 283.94 |
| Endogenous | Pou2f2 | NM_001163554.1 | 148.14 | 181.78 | 191.48 |
| Endogenous | Pparg | NM_011146.1 | 33.62 | 55.96 | 61.76 |
| Endogenous | Ppbp | NM_023785.2 | 1 | 5.79 | 3.8 |
| Endogenous | Prdm1 | NM_007548.3 | 1 | 9.77 | 9.32 |
| Endogenous | Prf1 | NM_011073.2 | 1 | 1 | 1 |
| Endogenous | Prim1 | NM_008921.2 | 170.89 | 193.73 | 188.03 |
| Endogenous | Prkcd | NM_011103.2 | 1902.57 | 1150.14 | 1107.09 |

| Endogenous | Psmb10 | NM_013640.3 | 92.1 | 91 | 112.82 |
| --- | --- | --- | --- | --- | --- |
| Endogenous | Psmb11 | NM_175204.4 | 1 | 1 | 1 |
| Endogenous | Psmb5 | NM_011186.1 | 2528.8 | 2507.12 | 2614.03 |
| Endogenous | Psmb7 | NM_011187.1 | 2364.73 | 2305.65 | 2304.91 |
| Endogenous | Psmb9 | NM_013585.2 | 36.06 | 31.27 | 23.81 |
| Endogenous | Psmc2 | NM_011188.3 | 3480.74 | 3482.65 | 3492.38 |
| Endogenous | Psmd7 | NM_010817.2 | 3102.24 | 3126.68 | 3162.57 |
| Endogenous | Ptafr | NM_001081211.1 | 24.68 | 20.92 | 32.78 |
| Endogenous | Ptger4 | NM_008965.1 | 1 | 1 | 1 |
| Endogenous | Ptgs2 | NM_011198.3 | 158.7 | 163.47 | 210.11 |
| Endogenous | Ptk2 | NM_007982.2 | 1737.68 | 1783.24 | 1982.69 |
| Endogenous | Ptpn2 | NM_001127177.1 | 513.65 | 470.06 | 467.47 |
| Endogenous | Ptpn22 | NM_008979.1 | 178.2 | 247.08 | 141.11 |
| Endogenous | Ptpn6 | NM_013545.2 | 52.3 | 70.29 | 65.9 |
| Endogenous | Ptprc | NM_011210.3 | 25.5 | 47.2 | 36.92 |
| Endogenous | Rae1 | NM_175112.5 | 1122.01 | 1136.6 | 1160.91 |
| Endogenous | Rag1 | NM_009019.2 | 1 | 1 | 1 |
| Endogenous | Rag2 | NM_009020.3 | 1 | 1 | 1 |
| Endogenous | Rela | NM_009045.4 | 398.31 | 322.74 | 315.68 |
| Endogenous | Relb | NM_009046.2 | 119.72 | 117.28 | 92.12 |
| Endogenous | Rorc | NM_011281.2 | 135.15 | 153.91 | 123.17 |
| Endogenous | Runx1 | NM_001111021.1 | 27.12 | 29.68 | 38.3 |
| Endogenous | Runx3 | NM_019732.2 | 1 | 1 | 1 |
| Endogenous | S100a8 | NM_013650.2 | 149.77 | 259.82 | 145.25 |
| Endogenous | S100a9 | NM_009114.2 | 48.24 | 114.89 | 79.7 |
| Endogenous | Sele | NM_011345.2 | 1 | 1 | 1 |
| Endogenous | Sell | NM_001164059.1 | 1 | 1 | 1 |
| Endogenous | Selplg | NM_009151.3 | 1 | 1 | 1 |
| Endogenous | Serping1 | NM_009776.3 | 121.34 | 103.74 | 66.59 |
| Endogenous | Sh2d1a | NM_011364.3 | 1 | 1 | 1 |
| Endogenous | Sigirr | NM_023059.3 | 42.55 | 28.88 | 26.57 |
| Endogenous | Ski | NM_011385.2 | 1189.43 | 1311.01 | 1278.21 |
| Endogenous | Slamf1 | NM_013730.4 | 1 | 1 | 1 |
| Endogenous | Slamf7 | NM_144539.5 | 1 | 1 | 1 |
| Endogenous | Smad3 | NM_016769.3 | 1044.04 | 1136.6 | 1141.59 |
| Endogenous | Smad5 | NM_008541.2 | 1303.95 | 1298.26 | 1220.25 |
| Endogenous | Socs1 | NM_009896.2 | 31.18 | 25.7 | 18.29 |
| Endogenous | Socs3 | NM_007707.2 | 6 | 10.57 | 14.15 |
| Endogenous | Spn | NM_001037810.1 | 1 | 1 | 1 |
| Endogenous | Src | NM_001025395.2 | 1210.54 | 1182.79 | 1186.44 |
| Endogenous | Stat1 | NM_009283.3 | 390.19 | 422.28 | 424.69 |
| Endogenous | Stat2 | NM_019963.1 | 314.65 | 353.79 | 292.22 |
| Endogenous | Stat3 | NM_213659.2 | 1024.54 | 1090.42 | 1069.14 |
| Endogenous | Stat4 | NM_011487.4 | 1 | 1 | 1 |
| Endogenous | Stat5a | NM_011488.2 | 42.55 | 55.96 | 44.51 |
| Endogenous | Stat5b | NM_011489.3 | 772.75 | 846.73 | 750.37 |
| Endogenous | Stat6 | NM_009284.2 | 206.62 | 229.56 | 192.17 |
| Endogenous | Syk | NM_011518.2 | 27.93 | 40.03 | 27.26 |

| Endogenous | Tagap | NM_145968.2 | 3.57 | 12.95 | 16.91 |
| --- | --- | --- | --- | --- | --- |
| Endogenous | Tal1 | NM_011527.2 | 96.16 | 114.89 | 124.55 |
| Endogenous | Tap1 | NM_001161730.1 | 57.17 | 47.2 | 55.55 |
| Endogenous | Tapbp | NM_009318.2 | 198.5 | 215.23 | 185.96 |
| Endogenous | Tbk1 | NM_019786.4 | 550.2 | 548.1 | 532.33 |
| Endogenous | Tbx21 | NM_019507.1 | 1 | 1 | 1 |
| Endogenous | Tcf4 | NM_013685.1 | 5966.17 | 6191.04 | 6124 |
| Endogenous | Tcf7 | NM_009331.3 | 56.36 | 82.24 | 66.59 |
| Endogenous | Tfrc | NM_011638.3 | 3676.48 | 3589.36 | 3604.85 |
| Endogenous | Tgfb1 | NM_011577.1 | 157.08 | 150.72 | 170.09 |
| Endogenous | Tgfb2 | NM_009367.1 | 230.18 | 190.54 | 190.79 |
| Endogenous | Tgfb3 | NM_009368.2 | 300.84 | 309.2 | 323.27 |
| Endogenous | Tgfbi | NM_009369.4 | 125.4 | 114.89 | 100.4 |
| Endogenous | Tgfbr1 | NM_009370.2 | 559.95 | 599.07 | 613.75 |
| Endogenous | Tgfbr2 | NM_009371.2 | 348.76 | 333.09 | 352.25 |
| Endogenous | Thy1 | NM_009382.3 | 7377.83 | 7450.07 | 7775.14 |
| Endogenous | Tigit | NM_001146325.1 | 1 | 1 | 1 |
| Endogenous | Tirap | NM_001177847.1 | 31.99 | 28.09 | 39.68 |
| Endogenous | Tlr1 | NM_030682.1 | 3.57 | 4.2 | 5.87 |
| Endogenous | Tlr2 | NM_011905.2 | 1 | 8.97 | 2.42 |
| Endogenous | Tlr3 | NM_126166.2 | 148.96 | 143.56 | 136.97 |
| Endogenous | Tlr4 | NM_021297.2 | 52.3 | 47.2 | 32.78 |
| Endogenous | Tlr5 | NM_016928.2 | 4.38 | 1 | 1 |
| Endogenous | Tlr8 | NM_133212.2 | 4.38 | 8.97 | 1.73 |
| Endogenous | Tlr9 | NM_031178.2 | 14.12 | 5.79 | 11.39 |
| Endogenous | Tmem173 | NM_028261.1 | 1 | 2.6 | 1 |
| Endogenous | Tnf | NM_013693.1 | 1 | 1 | 1 |
| Endogenous | Tnfaip3 | NM_009397.2 | 57.17 | 83.83 | 61.07 |
| Endogenous | Tnfaip6 | NM_009398.2 | 949.82 | 877.79 | 782.8 |
| Endogenous | Tnfrsf11a | NM_009399.3 | 253.73 | 212.84 | 197.69 |
| Endogenous | Tnfrsf13b | NM_021349.1 | 1 | 2.6 | 7.94 |
| Endogenous | Tnfrsf13c | NM_028075.2 | 1 | 1 | 1 |
| Endogenous | Tnfrsf14 | NM_178931.2 | 14.94 | 13.75 | 10.7 |
| Endogenous | Tnfrsf17 | NM_011608.1 | 1 | 10.57 | 2.42 |
| Endogenous | Tnfrsf1b | NM_011610.3 | 13.31 | 24.1 | 18.98 |
| Endogenous | Tnfrsf4 | NM_011659.2 | 9.25 | 32.07 | 32.09 |
| Endogenous | Tnfrsf8 | NM_009401.2 | 3.57 | 14.55 | 21.05 |
| Endogenous | Tnfrsf9 | NM_001077508.1 | 1 | 1 | 1 |
| Endogenous | Tnfsf10 | NM_009425.2 | 54.74 | 57.55 | 54.86 |
| Endogenous | Tnfsf11 | NM_011613.3 | 1 | 1 | 1 |
| Endogenous | Tnfsf12 | NM_011614.3 | 179.82 | 175.41 | 184.58 |
| Endogenous | Tnfsf13b | NM_033622.1 | 3.57 | 9.77 | 6.56 |
| Endogenous | Tnfsf14 | NM_019418.2 | 1 | 1 | 1 |
| Endogenous | Tnfsf15 | NM_177371.3 | 1 | 1 | 1 |
| Endogenous | Tnfsf18 | NM_183391.3 | 1 | 1 | 1 |
| Endogenous | Tnfsf8 | NM_009403.2 | 1 | 1 | 1 |
| Endogenous | Tollip | NM_023764.3 | 3347.53 | 3390.27 | 3379.91 |
| Endogenous | Traf1 | NM_009421.3 | 9.25 | 9.77 | 7.94 |

| Endogenous | Traf2 | NM_009422.2 | 48.24 | 47.2 | 38.3 |
| --- | --- | --- | --- | --- | --- |
| Endogenous | Traf3 | NM_001048206.1 | 559.95 | 622.96 | 602.02 |
| Endogenous | Traf4 | NM_009423.4 | 40.93 | 67.9 | 51.41 |
| Endogenous | Traf5 | NM_011633.1 | 68.54 | 62.33 | 79.01 |
| Endogenous | Traf6 | NM_009424.2 | 434.05 | 459.71 | 410.2 |
| Endogenous | Trem1 | NM_021406.3 | 1 | 1 | 1 |
| Endogenous | Trem2 | NM_031254.2 | 150.58 | 132.41 | 134.21 |
| Endogenous | Trp53 | NM_011640.1 | 169.26 | 213.64 | 188.03 |
| Endogenous | Tslp | NM_021367.1 | 1 | 1.81 | 3.11 |
| Endogenous | Tyk2 | NM_018793.2 | 87.23 | 89.4 | 78.32 |
| Endogenous | Tyrobp | NM_011662.2 | 729.7 | 751.17 | 804.88 |
| Endogenous | Ube2l3 | NM_009456.2 | 2597.84 | 2468.1 | 2481.55 |
| Endogenous | Vcam1 | NM_011693.2 | 473.04 | 444.58 | 481.27 |
| Endogenous | Vtn | NM_011707.2 | 479.53 | 423.87 | 432.28 |
| Endogenous | Xbp1 | NM_013842.2 | 2072.32 | 2138.41 | 2211.07 |
| Endogenous | Xcl1 | NM_008510.1 | 1 | 1 | 1 |
| Endogenous | Xcr1 | NM_011798.4 | 1 | 1 | 1 |
| Endogenous | Zap70 | NM_009539.2 | 22.25 | 15.34 | 16.91 |
| Endogenous | Zbtb7b | NM_009565.4 | 144.08 | 153.11 | 154.22 |
| Endogenous | Zeb1 | NM_011546.2 | 1681.64 | 1749 | 1702.55 |
| Housekeeping | Alas1 | NM_020559.2 | 505.53 | 507.49 | 486.1 |
| Housekeeping | Eef1g | NM_026007.4 | 1991.91 | 1999.05 | 1995.11 |
| Housekeeping | G6pdx | NM_008062.2 | 1009.11 | 947.87 | 882.85 |
| Housekeeping | Gapdh | NM_001001303.1 | 85956.52 | 86502.12 | 90027.22 |
| Housekeeping | Gusb | NM_010368.1 | 161.95 | 165.06 | 168.71 |
| Housekeeping | Hprt | NM_013556.2 | 2005.72 | 1981.53 | 1980.62 |
| Housekeeping | Oaz1 | NM_008753.4 | 10652.76 | 10520 | 10811.08 |
| Housekeeping | Polr1b | NM_009086.2 | 144.89 | 143.56 | 158.36 |
| Housekeeping | Polr2a | NM_009089.2 | 1650.77 | 1715.55 | 1605.26 |
| Housekeeping | Ppia | NM_008907.1 | 29035.22 | 28655.27 | 29204.79 |
| Housekeeping | Rpl19 | NM_009078.2 | 11361.84 | 11207.25 | 10988.41 |
| Housekeeping | Sdha | NM_023281.1 | 4058.23 | 4413.58 | 4489.41 |
| Housekeeping | Tbp | NM_013684.3 | 529.89 | 563.23 | 506.8 |
| Housekeeping | Tubb5 | NM_011655.4 | 624.11 | 568.81 | 619.27 |

| 1Young Repop 0h | 2Young Repop 0h | 3Young Repop 0h | 1Aged Con 0h | 2Aged Con 0h | 3Aged Con 0h |
| --- | --- | --- | --- | --- | --- |
| 349.23 | 328.98 | 384.59 | 337.11 | 376.25 | 324.78 |
| 1070.62 | 976.72 | 897.65 | 916.57 | 889.38 | 877.87 |
| 472.87 | 485.43 | 489.93 | 505.39 | 479.12 | 493.48 |
| 137.27 | 140.63 | 191.17 | 172 | 156.42 | 157.76 |
| 429.18 | 428.63 | 381.87 | 368.07 | 438.09 | 401.99 |
| 161.44 | 170.53 | 151.22 | 118.82 | 129.48 | 136.78 |
| 1 | 1 | 1 | 1 | 1 | 1 |
| 1 | 1 | 1 | 1 | 1 | 1 |
| 33887.35 | 33699.81 | 34169.43 | 35832.59 | 36005.44 | 34267.01 |
| 342.72 | 418.66 | 379.14 | 317.27 | 309.5 | 337.37 |
| 1580.05 | 1759 | 1486.99 | 1294.41 | 1351.08 | 1390.67 |
| 158.66 | 163.55 | 139.41 | 131.52 | 113.56 | 136.78 |
| 5568.15 | 5046.55 | 4910.42 | 8324.13 | 6499.58 | 6570.71 |
| 1 | 1 | 1 | 1 | 1 | 1 |
| 2.48 | 8.09 | 10.47 | 20.39 | 22.32 | 21.8 |
| 452.42 | 448.56 | 411.83 | 352.99 | 353.59 | 378.49 |
| 1782.71 | 1645.39 | 1733.99 | 1800.84 | 1749.1 | 1712.11 |
| 165.16 | 151.59 | 168.47 | 211.69 | 229.9 | 249.24 |
| 1 | 1 | 1 | 1 | 1 | 1 |
| 314.83 | 345.92 | 355.53 | 441.09 | 408.7 | 443.12 |
| 206.07 | 218.36 | 229.31 | 190.26 | 203.57 | 146.01 |
| 127.98 | 99.77 | 148.49 | 130.73 | 144.17 | 155.24 |
| 1 | 1 | 1 | 1 | 1 | 1 |
| 117.75 | 65.89 | 93.1 | 280.75 | 160.1 | 167.83 |
| 10.84 | 20.05 | 20.45 | 12.45 | 17.42 | 15.92 |
| 23.86 | 27.03 | 13.19 | 17.22 | 19.26 | 10.89 |
| 1 | 1 | 1 | 1 | 1 | 1 |
| 1 | 1 | 1 | 1 | 1 | 1 |
| 854.95 | 896 | 854.07 | 1112.63 | 1177.18 | 1129.65 |
| 1464.78 | 1465.02 | 1465.2 | 1673.84 | 1834.83 | 1741.49 |
| 1936.1 | 2018.1 | 1973.72 | 1674.63 | 1692.15 | 1712.11 |
| 29.44 | 27.03 | 29.54 | 72.78 | 33.34 | 63.76 |
| 47.1 | 33.01 | 38.62 | 50.55 | 49.26 | 65.44 |
| 27.58 | 44.96 | 40.43 | 47.38 | 54.16 | 71.32 |
| 6.2 | 7.1 | 34.98 | 80.72 | 49.88 | 66.28 |
| 445.91 | 284.13 | 356.44 | 1389.66 | 1259.84 | 1106.15 |
| 1 | 1 | 1 | 1 | 1 | 1 |
| 1 | 1 | 1 | 1 | 1 | 1 |
| 14.56 | 13.08 | 1 | 2.93 | 7.62 | 12.57 |
| 1 | 1 | 1 | 1 | 1 | 1 |
| 1 | 1 | 1 | 1 | 1 | 1 |
| 1 | 1.12 | 1 | 1 | 11.91 | 5.01 |
| 1 | 1 | 1 | 1 | 1 | 1 |
| 1 | 1 | 1 | 1 | 1 | 1 |
| 36.87 | 29.02 | 30.44 | 27.54 | 35.79 | 26 |
| 61.04 | 76.85 | 55.87 | 113.26 | 83.55 | 142.66 |
| 171.67 | 170.53 | 140.32 | 256.94 | 236.02 | 203.08 |

| 338.07 | 366.84 | 321.03 | 298.21 | 307.67 | 271.9 |
| --- | --- | --- | --- | --- | --- |
| 47.1 | 59.91 | 66.77 | 43.41 | 65.18 | 57.89 |
| 7.13 | 20.05 | 12.28 | 9.28 | 22.93 | 39.42 |
| 4.34 | 17.06 | 3.2 | 1 | 9.46 | 6.69 |
| 10.84 | 23.04 | 2.29 | 25.15 | 25.99 | 18.44 |
| 31.3 | 50.94 | 44.97 | 37.06 | 36.4 | 67.12 |
| 1 | 7.1 | 1 | 20.39 | 14.97 | 20.96 |
| 1 | 1 | 1 | 1 | 1 | 1 |
| 1 | 5.1 | 1 | 2.93 | 2.73 | 4.17 |
| 1 | 1 | 1 | 1 | 1 | 1 |
| 168.88 | 170.53 | 146.68 | 131.52 | 138.66 | 206.44 |
| 1 | 1 | 1 | 1 | 1 | 1 |
| 4.34 | 11.08 | 1 | 20.39 | 15.58 | 17.6 |
| 1 | 1 | 1 | 3.72 | 3.95 | 1 |
| 1 | 1 | 1 | 19.6 | 18.65 | 10.89 |
| 3.41 | 11.08 | 1.39 | 25.95 | 16.81 | 19.28 |
| 194.91 | 177.5 | 185.72 | 179.94 | 191.94 | 162.8 |
| 2.48 | 15.07 | 1 | 4.52 | 1.5 | 17.6 |
| 20.14 | 70.87 | 45.88 | 46.59 | 58.45 | 37.75 |
| 5.27 | 3.11 | 1.39 | 1 | 1.5 | 1 |
| 5.27 | 5.1 | 1 | 1 | 2.11 | 1 |
| 1 | 1 | 1 | 1 | 1 | 1 |
| 1 | 1 | 1 | 1 | 1 | 1 |
| 60.11 | 36.99 | 48.61 | 41.03 | 46.2 | 46.14 |
| 1 | 1 | 1 | 1 | 1 | 1 |
| 1 | 1 | 1 | 1 | 1 | 1 |
| 1 | 1 | 1 | 1 | 1 | 1 |
| 1 | 9.09 | 6.83 | 1 | 7.62 | 9.21 |
| 2.48 | 6.1 | 1 | 13.25 | 4.56 | 1.66 |
| 15.49 | 20.05 | 7.74 | 17.22 | 14.97 | 12.57 |
| 38.73 | 42.97 | 55.87 | 61.67 | 44.36 | 62.92 |
| 29.44 | 54.93 | 20.45 | 52.14 | 43.14 | 43.62 |
| 1 | 1 | 1 | 1 | 1 | 1 |
| 1 | 17.06 | 14.1 | 27.54 | 44.36 | 47.82 |
| 1911.93 | 1961.29 | 2010.04 | 1863.55 | 1825.64 | 1874.09 |
| 1 | 1 | 1 | 1 | 1 | 1 |
| 43.38 | 55.93 | 52.24 | 37.85 | 56 | 52.01 |
| 1 | 3.11 | 1 | 1 | 1 | 1 |
| 1 | 1.12 | 1 | 2.93 | 1 | 1 |
| 1 | 2.11 | 1 | 9.28 | 15.58 | 10.89 |
| 1 | 1.12 | 1 | 2.13 | 1 | 1 |
| 1 | 1 | 1 | 1 | 1 | 1 |
| 1 | 16.06 | 2.29 | 1.34 | 3.34 | 1 |
| 254.41 | 251.25 | 264.73 | 168.83 | 196.22 | 198.05 |
| 1 | 1 | 1 | 1 | 1 | 1 |
| 56.4 | 35 | 39.52 | 73.57 | 59.06 | 49.5 |
| 17.35 | 9.09 | 1 | 1 | 2.73 | 1 |
| 1730.65 | 1900.51 | 1722.18 | 1464.28 | 1430.07 | 1961.38 |

| 49.89 | 26.03 | 23.18 | 22.77 | 21.71 | 8.37 |
| --- | --- | --- | --- | --- | --- |
| 2.48 | 10.09 | 14.1 | 1 | 10.07 | 8.37 |
| 1 | 1 | 1 | 1 | 1 | 1 |
| 85.21 | 115.72 | 84.02 | 95.8 | 104.37 | 107.41 |
| 106.6 | 202.42 | 108.54 | 104.53 | 101.92 | 79.71 |
| 14.56 | 5.1 | 8.65 | 5.31 | 4.56 | 5.01 |
| 1 | 1 | 1 | 1 | 1 | 1 |
| 131.7 | 149.6 | 156.67 | 232.33 | 177.85 | 138.46 |
| 1 | 1 | 1 | 1 | 1 | 1 |
| 24.79 | 28.02 | 14.1 | 42.62 | 37.63 | 36.07 |
| 3.41 | 3.11 | 4.11 | 6.1 | 1.5 | 1 |
| 2.48 | 5.1 | 1 | 1 | 14.97 | 2.5 |
| 137.27 | 136.65 | 128.52 | 79.13 | 103.15 | 99.85 |
| 13.63 | 4.11 | 7.74 | 3.72 | 16.2 | 13.41 |
| 2.48 | 23.04 | 11.37 | 19.6 | 17.42 | 31.87 |
| 1 | 1 | 1 | 1 | 1 | 1 |
| 1 | 3.11 | 7.74 | 6.9 | 7.62 | 5.85 |
| 589.07 | 439.59 | 480.85 | 572.07 | 365.84 | 458.23 |
| 1 | 1 | 1 | 1 | 1 | 1 |
| 1 | 10.09 | 1 | 1 | 1 | 1 |
| 3.41 | 1.12 | 5.02 | 8.48 | 10.07 | 21.8 |
| 15400.81 | 14040.25 | 14525.09 | 14530.7 | 14505.21 | 13286.65 |
| 412.44 | 343.92 | 388.22 | 369.65 | 408.7 | 396.96 |
| 988.81 | 784.39 | 898.56 | 812.58 | 863.05 | 686.51 |
| 20.14 | 32.01 | 35.89 | 34.68 | 48.04 | 33.55 |
| 1 | 1 | 1 | 1 | 1 | 1 |
| 1 | 1 | 1 | 1 | 1 | 1 |
| 1586.56 | 1457.05 | 1658.62 | 1542.86 | 1388.43 | 1323.52 |
| 1 | 1 | 1 | 1 | 1 | 1 |
| 141.92 | 151.59 | 123.07 | 145.01 | 120.29 | 123.35 |
| 126.12 | 132.66 | 144.86 | 200.58 | 188.87 | 224.07 |
| 107.53 | 115.72 | 125.79 | 110.88 | 108.05 | 122.51 |
| 248.83 | 327.98 | 317.39 | 356.95 | 352.98 | 475.85 |
| 1.55 | 9.09 | 6.83 | 4.52 | 15.58 | 20.12 |
| 113.1 | 104.76 | 162.11 | 143.43 | 160.1 | 143.49 |
| 1 | 1 | 1 | 1.34 | 1 | 1 |
| 1 | 1 | 1 | 1 | 1 | 1 |
| 430.11 | 487.42 | 528.07 | 460.15 | 470.55 | 511.1 |
| 1 | 1 | 1 | 1 | 1 | 1 |
| 152.15 | 167.54 | 156.67 | 192.64 | 233.58 | 236.66 |
| 946.98 | 1014.59 | 953.95 | 853.86 | 876.52 | 944.17 |
| 1 | 1 | 1 | 1 | 1 | 1 |
| 27.58 | 22.04 | 38.62 | 21.19 | 24.16 | 25.16 |
| 1 | 1 | 1 | 1 | 1 | 1 |
| 1 | 1 | 1 | 1 | 1 | 1 |
| 33.16 | 52.94 | 30.44 | 33.09 | 46.81 | 41.94 |
| 24503.71 | 27031.02 | 28103.5 | 22201 | 23278.72 | 24678.22 |
| 26.65 | 57.92 | 40.43 | 55.32 | 57.22 | 52.85 |

| 1 | 1 | 1 | 1 | 1 | 1 |
| --- | --- | --- | --- | --- | --- |
| 207.93 | 191.45 | 166.65 | 218.84 | 200.51 | 162.8 |
| 657.86 | 679.75 | 649.75 | 727.65 | 758.96 | 725.96 |
| 515.63 | 535.26 | 532.61 | 611.76 | 566.68 | 576.56 |
| 657.86 | 807.31 | 896.74 | 919.74 | 1024.1 | 977.74 |
| 1 | 1 | 1 | 1 | 1 | 1 |
| 1 | 1 | 1 | 4.52 | 11.91 | 5.85 |
| 16.42 | 32.01 | 15.01 | 43.41 | 53.55 | 50.33 |
| 1 | 1.12 | 1 | 1 | 1 | 1 |
| 9241.1 | 9086.49 | 8895.05 | 6827.85 | 7025.57 | 7002.11 |
| 217.22 | 230.32 | 228.4 | 152.95 | 174.18 | 166.99 |
| 1 | 1 | 1 | 1 | 1 | 1 |
| 2165.72 | 2260.25 | 2316.06 | 2879.59 | 2774.14 | 2754.5 |
| 438.47 | 439.59 | 470.86 | 403.79 | 433.2 | 446.48 |
| 2384.18 | 2527.32 | 2691.1 | 2125.5 | 2433.69 | 2840.11 |
| 510.05 | 495.4 | 561.67 | 657 | 676.9 | 658.81 |
| 1 | 1 | 1 | 1 | 2.11 | 1 |
| 1 | 14.07 | 1 | 33.89 | 22.93 | 5.85 |
| 1 | 1 | 1 | 1 | 1 | 1 |
| 1574.48 | 1404.23 | 1378.93 | 1384.9 | 1378.64 | 1573.63 |
| 1 | 1 | 1 | 1 | 1 | 1 |
| 1 | 1 | 1 | 1 | 1 | 1 |
| 1 | 1 | 1 | 1 | 1 | 1 |
| 1 | 8.09 | 8.65 | 1 | 8.85 | 9.21 |
| 1 | 1 | 1 | 1 | 1 | 1 |
| 1 | 1 | 1 | 1 | 1 | 1 |
| 1 | 1 | 1 | 1 | 1 | 1 |
| 35.94 | 46.96 | 64.04 | 68.02 | 72.53 | 65.44 |
| 1 | 1 | 1 | 1 | 1 | 1 |
| 1 | 1 | 1 | 1 | 1 | 1 |
| 101.02 | 37.99 | 26.81 | 75.96 | 51.71 | 46.98 |
| 153.08 | 164.55 | 173.01 | 216.46 | 198.67 | 171.19 |
| 15.49 | 19.05 | 4.11 | 32.3 | 70.08 | 44.46 |
| 1 | 1 | 1 | 1 | 1 | 1 |
| 11.77 | 28.02 | 25 | 21.98 | 21.71 | 31.03 |
| 58.26 | 40.98 | 50.42 | 32.3 | 43.75 | 46.98 |
| 221.87 | 146.61 | 153.94 | 223.6 | 205.41 | 202.24 |
| 173.53 | 217.36 | 206.61 | 170.42 | 176.02 | 181.26 |
| 35.94 | 81.84 | 30.44 | 56.91 | 62.73 | 105.73 |
| 299.03 | 411.69 | 417.28 | 340.28 | 326.04 | 329.82 |
| 27.58 | 39.98 | 37.71 | 28.33 | 43.75 | 35.23 |
| 18.28 | 14.07 | 12.28 | 21.98 | 21.71 | 18.44 |
| 1 | 1 | 2.29 | 1 | 1 | 1 |
| 2.48 | 1 | 1 | 1 | 1 | 1 |
| 1 | 1 | 1 | 1 | 1 | 1 |
| 50.82 | 77.85 | 78.57 | 89.45 | 117.23 | 98.17 |
| 68.48 | 78.85 | 62.23 | 79.13 | 86 | 89.78 |
| 222.8 | 215.37 | 186.63 | 260.11 | 256.84 | 307.99 |

| 103.81 | 118.71 | 100.37 | 125.96 | 128.87 | 162.8 |
| --- | --- | --- | --- | --- | --- |
| 3.41 | 10.09 | 1 | 11.66 | 7.62 | 13.41 |
| 353.88 | 331.97 | 347.36 | 279.16 | 303.99 | 291.21 |
| 122.4 | 177.5 | 184.82 | 195.02 | 232.35 | 267.71 |
| 467.29 | 588.07 | 601.62 | 676.85 | 600.36 | 672.24 |
| 1 | 1 | 1 | 1 | 1 | 1 |
| 1 | 1.12 | 1 | 1 | 1 | 1 |
| 762.91 | 744.53 | 686.98 | 603.03 | 572.2 | 615.17 |
| 3834.4 | 3956.35 | 3761.71 | 3141.54 | 3204 | 3308.43 |
| 54.54 | 61.91 | 32.26 | 20.39 | 70.08 | 40.26 |
| 1 | 1 | 1 | 1 | 1 | 1 |
| 8.99 | 13.08 | 8.65 | 16.42 | 11.91 | 20.12 |
| 107.53 | 139.63 | 114.89 | 81.51 | 81.1 | 102.37 |
| 188.4 | 188.46 | 206.61 | 205.34 | 239.09 | 208.96 |
| 2.48 | 1 | 1 | 1 | 17.42 | 9.21 |
| 1 | 1 | 1 | 1 | 1 | 1 |
| 1 | 1 | 1 | 1 | 1 | 1 |
| 1 | 1 | 1 | 1 | 1 | 1 |
| 336.21 | 241.28 | 269.27 | 250.59 | 157.03 | 191.33 |
| 129.84 | 101.77 | 90.38 | 119.61 | 73.14 | 95.66 |
| 262.77 | 304.06 | 289.24 | 223.6 | 212.76 | 203.08 |
| 1 | 12.08 | 1 | 6.9 | 8.85 | 7.53 |
| 1 | 1 | 1 | 1 | 1 | 1 |
| 45.24 | 47.95 | 58.59 | 100.56 | 56 | 66.28 |
| 253.48 | 255.23 | 266.54 | 653.83 | 444.83 | 424.65 |
| 5.27 | 8.09 | 1.39 | 4.52 | 11.3 | 7.53 |
| 1 | 1 | 1 | 1 | 1 | 1 |
| 1 | 1 | 1 | 1 | 1 | 1 |
| 1 | 1 | 1 | 1 | 1 | 1 |
| 1 | 1 | 1 | 1 | 1 | 1 |
| 1 | 1 | 1 | 1 | 1 | 1 |
| 244.18 | 232.31 | 236.58 | 250.59 | 209.08 | 197.21 |
| 1159.86 | 1038.51 | 1082.9 | 1198.36 | 1019.81 | 981.1 |
| 1 | 1 | 7.74 | 6.9 | 10.69 | 11.73 |
| 15.49 | 19.05 | 21.36 | 33.89 | 40.69 | 43.62 |
| 140.99 | 140.63 | 138.5 | 98.98 | 97.64 | 77.19 |
| 1.55 | 13.08 | 15.01 | 14.83 | 14.97 | 25.16 |
| 591.86 | 750.51 | 791.41 | 540.32 | 594.24 | 739.38 |
| 1 | 1 | 1 | 1 | 1 | 1 |
| 176.32 | 129.67 | 138.5 | 210.1 | 161.32 | 191.33 |
| 8.06 | 1 | 1 | 5.31 | 3.34 | 1.66 |
| 90.79 | 78.85 | 53.15 | 104.53 | 104.37 | 97.33 |
| 194.91 | 176.51 | 165.75 | 202.96 | 185.2 | 182.1 |
| 319.48 | 335.95 | 276.53 | 281.55 | 286.85 | 307.15 |
| 79.64 | 136.65 | 126.7 | 115.64 | 147.24 | 148.53 |
| 1 | 1 | 1 | 1 | 1 | 1 |
| 1 | 1 | 1 | 1 | 1 | 1 |
| 1502.89 | 1623.47 | 1583.25 | 1537.31 | 1413.54 | 1493.9 |

| 405.94 | 420.66 | 385.5 | 383.94 | 370.13 | 406.19 |
| --- | --- | --- | --- | --- | --- |
| 1 | 1 | 1 | 1 | 1 | 1 |
| 1 | 1 | 1 | 1 | 1 | 1 |
| 548.17 | 538.25 | 501.73 | 467.29 | 469.94 | 442.28 |
| 797.31 | 770.44 | 844.08 | 837.19 | 844.07 | 849.33 |
| 299.96 | 314.03 | 260.19 | 365.69 | 378.7 | 328.98 |
| 539.8 | 488.42 | 542.6 | 690.34 | 601.59 | 630.28 |
| 432.89 | 393.75 | 356.44 | 341.87 | 395.23 | 407.03 |
| 9.91 | 25.03 | 27.72 | 30.71 | 24.16 | 25.16 |
| 335.28 | 324.99 | 319.21 | 346.64 | 348.69 | 347.44 |
| 1 | 25.03 | 5.93 | 11.66 | 21.71 | 19.28 |
| 126.12 | 134.65 | 141.23 | 118.82 | 148.46 | 151.05 |
| 1 | 1 | 1 | 1 | 1 | 1 |
| 255.34 | 293.1 | 263.82 | 256.14 | 264.19 | 287.01 |
| 1 | 1 | 1 | 1 | 1 | 1 |
| 86.14 | 81.84 | 108.54 | 102.15 | 113.56 | 104.89 |
| 300.89 | 327.98 | 309.22 | 256.94 | 257.46 | 274.42 |
| 13.63 | 13.08 | 14.1 | 22.77 | 22.93 | 17.6 |
| 32.23 | 21.05 | 25 | 12.45 | 11.3 | 36.07 |
| 1 | 2.11 | 1 | 1 | 1 | 1 |
| 1 | 1 | 1 | 1 | 1 | 1 |
| 1 | 1 | 1 | 1 | 1 | 1 |
| 14.56 | 4.11 | 1.39 | 1 | 1 | 1 |
| 233.03 | 238.29 | 227.5 | 183.91 | 195 | 196.37 |
| 1 | 1 | 1 | 12.45 | 5.17 | 8.37 |
| 15.49 | 22.04 | 11.37 | 31.5 | 36.4 | 32.71 |
| 231.17 | 168.53 | 142.14 | 460.94 | 303.38 | 226.58 |
| 1 | 1 | 1 | 1 | 1 | 1 |
| 1 | 1 | 1 | 1 | 1 | 1 |
| 1 | 2.11 | 1.39 | 1 | 2.11 | 1 |
| 200.49 | 211.39 | 220.23 | 249.79 | 209.69 | 279.46 |
| 17.35 | 30.02 | 24.09 | 16.42 | 30.89 | 24.32 |
| 4.34 | 8.09 | 1 | 23.57 | 8.85 | 1 |
| 1389.48 | 1423.17 | 1343.52 | 995.15 | 1019.81 | 1049.92 |
| 1 | 1 | 1 | 1 | 1 | 1 |
| 1 | 1 | 1 | 1 | 1 | 1 |
| 1 | 1 | 1 | 1 | 1 | 1 |
| 2.48 | 19.05 | 1 | 6.9 | 8.24 | 7.53 |
| 1 | 4.11 | 1 | 1 | 6.4 | 1 |
| 129.84 | 129.67 | 120.34 | 147.4 | 127.03 | 152.73 |
| 1.55 | 7.1 | 1 | 2.93 | 1 | 2.5 |
| 880.97 | 791.37 | 803.21 | 746.7 | 735.08 | 779.67 |
| 4.34 | 2.11 | 1.39 | 1 | 4.56 | 1.66 |
| 1 | 7.1 | 1 | 4.52 | 1 | 7.53 |
| 1 | 1 | 1 | 1 | 1 | 1 |
| 1 | 1 | 1 | 1 | 1 | 1 |
| 1 | 1 | 1 | 1 | 1 | 1 |
| 1 | 1.12 | 1.39 | 1 | 1 | 1 |

| 1 | 2.11 | 1 | 1 | 8.24 | 6.69 |
| --- | --- | --- | --- | --- | --- |
| 336.21 | 215.37 | 108.54 | 316.47 | 199.28 | 224.91 |
| 1 | 1 | 1 | 1 | 1 | 1 |
| 1 | 1 | 1 | 1 | 1 | 1 |
| 1 | 1 | 1 | 1 | 1 | 1 |
| 1 | 1 | 1 | 1 | 1 | 1 |
| 1 | 1 | 1 | 1 | 1 | 1 |
| 16.42 | 11.08 | 5.02 | 1 | 1.5 | 3.33 |
| 1 | 1 | 1 | 1 | 1 | 1 |
| 1 | 1 | 1 | 1 | 1 | 1 |
| 1 | 6.1 | 1 | 5.31 | 6.4 | 3.33 |
| 1 | 10.09 | 1.39 | 12.45 | 5.17 | 5.01 |
| 1 | 1 | 1 | 1 | 1 | 1 |
| 1203.55 | 1136.17 | 1268.15 | 1483.33 | 1420.89 | 1217.78 |
| 1 | 1 | 1 | 1 | 1 | 1 |
| 169.81 | 136.65 | 105.81 | 186.29 | 176.02 | 198.89 |
| 1 | 1 | 1 | 1 | 1 | 1 |
| 1 | 11.08 | 1 | 1 | 1 | 1.66 |
| 97.3 | 99.77 | 97.64 | 102.94 | 109.27 | 108.24 |
| 854.02 | 873.08 | 881.31 | 819.73 | 807.94 | 835.06 |
| 3.41 | 10.09 | 1 | 1 | 2.11 | 1 |
| 13.63 | 4.11 | 8.65 | 7.69 | 7.01 | 12.57 |
| 1 | 1 | 1 | 1 | 1 | 1 |
| 517.49 | 606.01 | 603.44 | 638.75 | 657.31 | 644.55 |
| 563.97 | 508.35 | 558.94 | 630.81 | 649.35 | 629.44 |
| 411.51 | 500.38 | 479.94 | 473.64 | 479.73 | 457.39 |
| 41.52 | 38.99 | 55.87 | 65.64 | 48.04 | 61.25 |
| 91.72 | 112.73 | 94.01 | 81.51 | 88.45 | 98.17 |
| 129.84 | 129.67 | 124.88 | 164.07 | 147.24 | 122.51 |
| 9.91 | 9.09 | 7.74 | 23.57 | 15.58 | 12.57 |
| 1 | 1 | 1 | 1 | 5.79 | 1 |
| 39.66 | 55.93 | 35.89 | 74.37 | 64.57 | 62.08 |
| 1 | 27.03 | 10.47 | 66.43 | 40.69 | 16.76 |
| 26.65 | 37.99 | 31.35 | 52.14 | 73.76 | 51.17 |
| 342.72 | 352.89 | 311.04 | 533.97 | 408.09 | 396.12 |
| 1 | 1 | 1 | 1 | 1 | 1 |
| 213.5 | 267.19 | 226.59 | 137.87 | 228.68 | 224.91 |
| 3.41 | 15.07 | 9.56 | 17.22 | 13.75 | 15.08 |
| 600.23 | 638.9 | 633.4 | 650.65 | 615.67 | 573.21 |
| 11.77 | 6.1 | 1 | 17.22 | 3.34 | 2.5 |
| 124.26 | 158.57 | 143.95 | 195.02 | 215.21 | 173.71 |
| 19.21 | 7.1 | 1 | 40.24 | 21.1 | 18.44 |
| 1777.13 | 1825.77 | 1747.61 | 1419.03 | 1390.88 | 1426.76 |
| 14.56 | 19.05 | 8.65 | 44.2 | 44.98 | 32.71 |
| 1 | 1 | 1 | 1 | 1.5 | 1 |
| 4017.53 | 3956.35 | 3910.64 | 3174.08 | 3114.6 | 3269.82 |
| 1136.62 | 1178.02 | 1129.21 | 1061.04 | 1032.67 | 1229.53 |
| 25.72 | 35 | 43.16 | 35.47 | 79.88 | 71.32 |

| 1 | 1 | 1 | 1 | 1 | 1 |
| --- | --- | --- | --- | --- | --- |
| 1 | 2.11 | 1 | 1 | 1 | 1 |
| 359.45 | 356.88 | 309.22 | 416.49 | 334.61 | 293.73 |
| 1 | 1 | 1 | 1 | 1 | 1 |
| 1 | 1 | 1 | 1 | 1 | 1 |
| 1 | 1 | 1 | 1 | 1 | 1 |
| 1 | 1 | 1 | 1 | 1 | 1 |
| 1 | 1 | 1 | 1 | 1 | 1 |
| 1 | 1 | 1 | 1 | 1 | 1 |
| 1 | 1 | 1 | 1 | 1 | 1 |
| 1 | 1 | 1 | 1 | 1 | 1 |
| 1 | 1 | 1 | 1 | 1 | 1 |
| 1 | 1.12 | 1 | 1 | 1 | 1 |
| 1 | 1 | 1 | 1 | 1 | 1 |
| 1 | 1 | 1 | 1 | 1 | 1 |
| 1 | 8.09 | 1 | 1 | 1 | 1 |
| 116.82 | 117.71 | 83.11 | 125.96 | 123.97 | 105.73 |
| 10.84 | 10.09 | 18.64 | 10.07 | 18.65 | 23.48 |
| 41.52 | 41.97 | 28.63 | 24.36 | 27.83 | 35.23 |
| 191.19 | 150.6 | 186.63 | 177.56 | 246.43 | 165.32 |
| 1 | 6.1 | 1 | 6.1 | 18.03 | 11.73 |
| 3.41 | 4.11 | 1 | 1 | 8.85 | 5.01 |
| 1 | 1 | 1 | 1 | 1 | 1 |
| 1 | 1 | 1 | 1 | 1 | 5.01 |
| 43.38 | 7.1 | 1 | 41.82 | 27.22 | 33.55 |
| 315.76 | 268.19 | 312.85 | 298.21 | 315.63 | 240.01 |
| 5.27 | 1.12 | 1 | 1 | 3.34 | 1 |
| 7.13 | 11.08 | 12.28 | 6.9 | 12.52 | 14.25 |
| 1 | 1 | 1 | 1 | 1 | 1 |
| 1 | 1 | 1 | 1 | 1 | 1 |
| 100.09 | 101.77 | 108.54 | 95.01 | 88.45 | 95.66 |
| 1 | 1 | 1 | 1 | 1 | 1 |
| 425.46 | 449.56 | 411.83 | 477.61 | 460.75 | 442.28 |
| 63.83 | 54.93 | 77.66 | 59.29 | 57.22 | 71.32 |
| 178.18 | 192.45 | 181.18 | 225.98 | 209.69 | 246.73 |
| 1 | 1 | 1 | 1 | 1 | 1 |
| 507.26 | 464.5 | 480.85 | 576.83 | 566.68 | 526.21 |
| 1093.86 | 1065.41 | 1183.7 | 1026.9 | 1024.71 | 1082.65 |
| 9657.57 | 10961.96 | 10130.93 | 7042.97 | 7192.12 | 7780.12 |
| 67.55 | 85.82 | 72.21 | 122 | 123.97 | 146.85 |
| 907 | 1020.57 | 973.93 | 895.93 | 907.75 | 1086.85 |
| 357.6 | 331.97 | 415.47 | 421.25 | 416.05 | 394.44 |
| 1 | 1 | 1 | 1 | 1 | 1 |
| 93.58 | 119.7 | 102.18 | 103.74 | 89.06 | 104.05 |
| 1 | 1 | 1 | 1 | 2.73 | 3.33 |
| 1 | 1 | 1 | 1 | 1 | 1 |
| 7327 | 6760.59 | 8090.49 | 9479.87 | 9490.21 | 8771.31 |
| 4796.56 | 4975.8 | 5217.35 | 5943.58 | 5943.58 | 5560.22 |

| 282.3 | 317.02 | 259.28 | 205.34 | 210.92 | 203.08 |
| --- | --- | --- | --- | --- | --- |
| 182.83 | 151.59 | 176.64 | 183.91 | 183.36 | 207.28 |
| 1 | 1.12 | 1 | 1 | 1 | 1 |
| 1 | 2.11 | 1 | 1 | 5.79 | 4.17 |
| 2.48 | 7.1 | 2.29 | 1 | 11.91 | 4.17 |
| 209.78 | 212.38 | 221.14 | 284.72 | 229.29 | 259.32 |
| 73.13 | 75.86 | 71.31 | 86.28 | 73.76 | 88.1 |
| 6557.27 | 6460.63 | 6596.72 | 6211.08 | 6602.45 | 6031.9 |
| 20.14 | 22.04 | 19.55 | 21.98 | 10.07 | 18.44 |
| 20.14 | 15.07 | 29.54 | 25.15 | 36.4 | 41.94 |
| 246.04 | 264.2 | 232.94 | 226.77 | 236.64 | 224.07 |
| 262.77 | 234.31 | 253.83 | 288.69 | 249.5 | 254.28 |
| 314.83 | 357.88 | 318.3 | 275.99 | 304.61 | 352.48 |
| 136.34 | 117.71 | 129.42 | 149.78 | 168.67 | 144.33 |
| 57.33 | 53.93 | 51.33 | 66.43 | 62.12 | 66.28 |
| 284.15 | 301.07 | 358.26 | 356.16 | 299.71 | 342.4 |
| 98.23 | 145.61 | 101.27 | 164.07 | 162.54 | 165.32 |
| 1 | 3.11 | 1 | 1 | 13.14 | 1 |
| 1.55 | 16.06 | 1 | 9.28 | 1 | 1 |
| 277.65 | 307.05 | 296.51 | 373.62 | 401.35 | 337.37 |
| 219.08 | 257.23 | 272.9 | 258.53 | 235.41 | 249.24 |
| 1 | 1 | 1 | 1 | 1 | 1 |
| 1 | 1 | 1 | 1 | 1 | 1 |
| 4.34 | 4.11 | 5.02 | 6.1 | 7.01 | 1 |
| 2062.53 | 2083.87 | 2101.76 | 1846.88 | 1998.32 | 1911.86 |
| 117.75 | 177.5 | 114.89 | 109.29 | 108.05 | 109.92 |
| 1 | 21.05 | 1 | 2.93 | 12.52 | 1.66 |
| 1 | 5.1 | 1 | 1 | 1 | 1 |
| 1 | 1 | 1 | 1 | 1 | 1 |
| 104.74 | 93.79 | 99.46 | 93.42 | 115.39 | 83.91 |
| 435.68 | 558.18 | 559.85 | 499.04 | 442.38 | 552.22 |
| 147.5 | 166.54 | 176.64 | 201.37 | 182.75 | 199.73 |
| 560.25 | 619.96 | 587.09 | 584.77 | 503 | 530.4 |
| 2492.02 | 2557.22 | 2552.16 | 2394.59 | 2357.14 | 2189.66 |
| 923.74 | 988.68 | 1059.29 | 956.26 | 1005.73 | 1021.38 |
| 1 | 1 | 1 | 1 | 1 | 1 |
| 1 | 1 | 1 | 1 | 1 | 1 |
| 1 | 1 | 1 | 1 | 1 | 1 |
| 41.52 | 36 | 34.08 | 45 | 44.36 | 46.98 |
| 23.86 | 43.97 | 28.63 | 47.38 | 29.67 | 39.42 |
| 317.62 | 366.84 | 296.51 | 306.95 | 288.07 | 311.35 |
| 147.5 | 134.65 | 165.75 | 168.83 | 163.16 | 197.21 |
| 48.96 | 49.95 | 37.71 | 58.49 | 51.1 | 81.39 |
| 1 | 1 | 1 | 2.93 | 12.52 | 9.21 |
| 3.41 | 1.12 | 1 | 1.34 | 8.24 | 10.89 |
| 1 | 1 | 1 | 1 | 1 | 1 |
| 192.12 | 165.54 | 154.85 | 168.83 | 177.85 | 179.58 |
| 1547.52 | 716.63 | 1177.34 | 1818.3 | 1547.64 | 1284.92 |

| 61.04 | 94.79 | 59.5 | 114.85 | 102.54 | 100.69 |
| --- | --- | --- | --- | --- | --- |
| 1 | 1 | 1 | 1 | 1 | 1 |
| 2713.27 | 2731.61 | 2806.42 | 2245.36 | 2281.22 | 2448.16 |
| 2782.06 | 2723.64 | 2709.26 | 2133.43 | 2028.32 | 2184.63 |
| 42.45 | 36.99 | 22.27 | 65.64 | 58.45 | 50.33 |
| 4023.11 | 4071.95 | 3900.65 | 3162.97 | 3239.52 | 3243.8 |
| 3574.1 | 3654.4 | 3573.74 | 2891.49 | 2993.36 | 3108.68 |
| 13.63 | 25.03 | 17.73 | 37.06 | 40.08 | 34.39 |
| 1 | 3.11 | 1 | 1 | 5.17 | 1 |
| 188.4 | 255.23 | 270.17 | 183.91 | 193.16 | 251.76 |
| 1974.22 | 2121.73 | 1931.04 | 1738.93 | 1735.01 | 2210.65 |
| 607.66 | 574.12 | 562.57 | 463.32 | 468.1 | 503.55 |
| 244.18 | 167.54 | 74.03 | 292.66 | 268.48 | 265.19 |
| 84.29 | 72.87 | 56.78 | 107.71 | 96.41 | 70.48 |
| 35.01 | 49.95 | 36.8 | 50.55 | 42.53 | 52.01 |
| 1111.52 | 1064.42 | 1101.06 | 1096.76 | 1073.08 | 1000.4 |
| 1 | 1 | 1 | 1 | 1 | 2.5 |
| 1 | 1 | 1 | 1 | 1 | 1 |
| 413.37 | 366.84 | 350.99 | 364.1 | 333.39 | 334.01 |
| 106.6 | 127.68 | 119.43 | 140.25 | 112.95 | 140.98 |
| 129.84 | 117.71 | 106.72 | 232.33 | 174.18 | 151.89 |
| 29.44 | 36.99 | 24.09 | 47.38 | 46.2 | 60.41 |
| 1 | 1 | 1 | 1 | 1 | 1 |
| 76.85 | 122.69 | 143.04 | 187.88 | 207.24 | 139.3 |
| 50.82 | 105.75 | 73.12 | 107.71 | 119.07 | 78.87 |
| 1 | 1 | 1 | 1 | 1 | 1 |
| 1 | 1 | 1 | 1 | 2.11 | 1 |
| 1 | 1 | 1 | 1 | 1 | 1 |
| 76.85 | 76.85 | 113.99 | 140.25 | 122.74 | 135.94 |
| 1 | 1 | 1 | 1 | 1 | 1 |
| 19.21 | 34 | 25 | 23.57 | 36.4 | 34.39 |
| 946.98 | 1089.33 | 1087.44 | 1434.11 | 1180.24 | 1367.17 |
| 1 | 1 | 1 | 1 | 1 | 1 |
| 1 | 1 | 1 | 1 | 5.17 | 1 |
| 1055.74 | 1231.83 | 1169.17 | 1037.22 | 974.5 | 1250.51 |
| 1465.71 | 1496.91 | 1378.93 | 1223.76 | 1228 | 1358.77 |
| 36.87 | 42.97 | 25.9 | 45.79 | 34.57 | 31.87 |
| 8.99 | 28.02 | 13.19 | 14.04 | 24.16 | 36.07 |
| 1 | 1 | 1 | 4.52 | 1 | 1 |
| 958.13 | 1068.4 | 1056.57 | 1237.26 | 1249.44 | 1265.61 |
| 365.03 | 371.83 | 374.6 | 660.18 | 543.42 | 509.42 |
| 228.38 | 249.25 | 223.86 | 414.11 | 345.63 | 341.57 |
| 1106.87 | 1075.38 | 1185.51 | 1115.81 | 1171.67 | 1183.36 |
| 1 | 1 | 1 | 1 | 3.34 | 2.5 |
| 44.31 | 34 | 41.34 | 83.89 | 49.26 | 55.37 |
| 834.49 | 771.44 | 744.19 | 862.59 | 809.17 | 820.8 |
| 196.77 | 207.4 | 236.58 | 218.84 | 247.05 | 255.12 |
| 44.31 | 59.91 | 58.59 | 36.27 | 46.2 | 52.01 |

| 10.84 | 25.03 | 15.91 | 25.95 | 21.71 | 10.05 |
| --- | --- | --- | --- | --- | --- |
| 101.02 | 86.82 | 125.79 | 68.81 | 141.11 | 66.28 |
| 61.04 | 59.91 | 59.5 | 135.49 | 93.35 | 94.82 |
| 186.54 | 227.33 | 203.89 | 218.84 | 204.18 | 215.67 |
| 545.38 | 585.08 | 577.1 | 509.36 | 491.98 | 512.78 |
| 1 | 1 | 1 | 1 | 2.73 | 9.21 |
| 6704.15 | 7287.75 | 6742.01 | 5976.92 | 5728.65 | 6908.11 |
| 47.1 | 61.91 | 64.04 | 63.26 | 65.18 | 67.12 |
| 4424.71 | 3912.5 | 3701.78 | 2806.56 | 2697.6 | 2556.43 |
| 187.47 | 151.59 | 154.85 | 202.17 | 172.34 | 176.23 |
| 234.88 | 234.31 | 261.09 | 225.19 | 204.18 | 175.39 |
| 237.67 | 278.15 | 257.46 | 337.9 | 384.21 | 408.71 |
| 134.48 | 99.77 | 134.87 | 137.08 | 128.87 | 149.37 |
| 599.3 | 635.91 | 605.25 | 601.44 | 640.78 | 590.83 |
| 375.26 | 344.92 | 353.72 | 429.19 | 377.47 | 450.67 |
| 7493.41 | 7639.53 | 7842.59 | 6700.05 | 6616.53 | 6578.27 |
| 1 | 1 | 1 | 1 | 1 | 1 |
| 39.66 | 36 | 29.54 | 48.17 | 52.32 | 45.3 |
| 1 | 14.07 | 4.11 | 1 | 10.69 | 10.89 |
| 13.63 | 14.07 | 20.45 | 18.8 | 32.73 | 24.32 |
| 136.34 | 158.57 | 138.5 | 143.43 | 176.02 | 150.21 |
| 48.96 | 42.97 | 52.24 | 40.24 | 57.22 | 67.96 |
| 1 | 6.1 | 5.02 | 1 | 7.01 | 5.85 |
| 2.48 | 2.11 | 1 | 1 | 1.5 | 1.66 |
| 14.56 | 16.06 | 15.01 | 26.74 | 22.93 | 16.76 |
| 1 | 3.11 | 1 | 1 | 6.4 | 3.33 |
| 1 | 1 | 1 | 1 | 1 | 1 |
| 49.89 | 93.79 | 77.66 | 47.38 | 59.67 | 65.44 |
| 787.08 | 828.24 | 806.85 | 758.61 | 758.96 | 729.31 |
| 236.74 | 218.36 | 232.94 | 221.22 | 202.96 | 208.96 |
| 7.13 | 16.06 | 1 | 4.52 | 11.91 | 9.21 |
| 1 | 5.1 | 1 | 1 | 1 | 1 |
| 8.06 | 27.03 | 7.74 | 18.8 | 24.16 | 24.32 |
| 1 | 1 | 1 | 2.93 | 8.24 | 1 |
| 13.63 | 17.06 | 6.83 | 25.95 | 37.63 | 33.55 |
| 10.84 | 22.04 | 13.19 | 25.95 | 33.95 | 28.51 |
| 9.91 | 9.09 | 16.82 | 7.69 | 15.58 | 15.08 |
| 1 | 1 | 1 | 1 | 1 | 1 |
| 67.55 | 50.94 | 64.04 | 75.16 | 50.49 | 42.78 |
| 1 | 1 | 1 | 1 | 1 | 1 |
| 167.02 | 162.56 | 159.39 | 226.77 | 201.73 | 206.44 |
| 14.56 | 10.09 | 10.47 | 14.04 | 27.22 | 5.85 |
| 1 | 1 | 1 | 1 | 1 | 1 |
| 1 | 3.11 | 1 | 1 | 1 | 1 |
| 1 | 1 | 1 | 1 | 1 | 1 |
| 1 | 1 | 1 | 1 | 1 | 1 |
| 3382.6 | 3311.59 | 3323.11 | 3130.42 | 3083.37 | 3183.37 |
| 12.7 | 1 | 6.83 | 6.1 | 12.52 | 11.73 |

| 25.72 | 25.03 | 38.62 | 40.24 | 43.14 | 44.46 |
| --- | --- | --- | --- | --- | --- |
| 558.39 | 604.02 | 606.16 | 514.92 | 554.44 | 574.89 |
| 46.17 | 62.9 | 41.34 | 60.08 | 56.61 | 41.1 |
| 50.82 | 91.8 | 61.32 | 83.1 | 56 | 69.64 |
| 431.04 | 458.52 | 433.63 | 452.21 | 441.77 | 451.51 |
| 1 | 1 | 1 | 1 | 1 | 1 |
| 133.56 | 134.65 | 137.6 | 270.43 | 247.05 | 198.05 |
| 140.99 | 161.56 | 179.37 | 180.73 | 164.99 | 190.49 |
| 1 | 1 | 1 | 1 | 1 | 1.66 |
| 51.75 | 72.87 | 76.76 | 86.28 | 86 | 70.48 |
| 1208.2 | 1022.56 | 980.29 | 1231.7 | 1209.63 | 1193.44 |
| 2704.9 | 2626.98 | 2698.36 | 2423.16 | 2474.1 | 2426.34 |
| 483.09 | 449.56 | 517.17 | 477.61 | 370.13 | 325.62 |
| 490.53 | 450.55 | 492.65 | 413.31 | 403.19 | 463.26 |
| 2558.95 | 2372.86 | 2487.69 | 2088.98 | 2475.94 | 2628.61 |
| 1 | 1 | 1 | 1 | 1 | 1 |
| 1 | 1 | 1 | 1 | 1 | 1 |
| 14.56 | 7.1 | 8.65 | 14.04 | 16.81 | 22.64 |
| 126.12 | 98.78 | 130.33 | 157.72 | 160.1 | 118.32 |
| 1874.75 | 1868.62 | 1798.46 | 1670.66 | 1619.28 | 1599.65 |
| 364.1 | 429.63 | 409.11 | 544.29 | 558.11 | 542.99 |
| 2297.73 | 2325.03 | 2208 | 1858.79 | 2022.2 | 1986.56 |
| 933.96 | 778.41 | 823.19 | 1006.27 | 1040.63 | 928.22 |
| 83083.16 | 85437.7 | 86286.43 | 84456.38 | 83717.18 | 86787.59 |
| 202.35 | 198.43 | 206.61 | 187.09 | 212.14 | 208.12 |
| 2063.46 | 1848.69 | 1968.27 | 2030.24 | 2001.99 | 1993.27 |
| 10641.12 | 10729.77 | 10862.84 | 10060.12 | 10101.32 | 10342.45 |
| 147.5 | 166.54 | 141.23 | 160.1 | 149.07 | 152.73 |
| 1644.2 | 1559.69 | 1610.49 | 1664.31 | 1602.14 | 1443.54 |
| 34107.67 | 34859.77 | 32957.15 | 26404.06 | 26247.92 | 27760.06 |
| 11070.61 | 11079.55 | 11096.21 | 10926.93 | 11498.66 | 11775.1 |
| 4254.59 | 4215.45 | 4562.63 | 4133.76 | 4138.42 | 4200.58 |
| 609.52 | 583.09 | 563.48 | 490.31 | 476.67 | 569.85 |
| 467.29 | 496.39 | 528.07 | 625.25 | 533.62 | 497.67 |

| 1Aged Repop 0h | 2Aged Repop 0h | 3Aged Repop 0h | 1Young Con 6h | 2Young Con 6h | 3Young Con 6h |
| --- | --- | --- | --- | --- | --- |
| 338.67 | 337.86 | 353.85 | 365.21 | 346.29 | 328.6 |
| 1177.93 | 998.98 | 912.12 | 230.75 | 206.65 | 211.22 |
| 484.87 | 468.49 | 436.88 | 704.9 | 744.18 | 634.12 |
| 169.92 | 166.6 | 171.88 | 152.9 | 131.57 | 150.12 |
| 432.53 | 449.37 | 405.96 | 250.21 | 271.21 | 212.83 |
| 106.75 | 145.1 | 102.09 | 124.6 | 134.58 | 166.2 |
| 1 | 1 | 1 | 1.77 | 1.5 | 1.61 |
| 1 | 1 | 1 | 1.77 | 1.5 | 2.18 |
| 33942.28 | 35055.56 | 36579.64 | 27659.03 | 26775.45 | 30235.25 |
| 417.19 | 350.6 | 336.18 | 342.21 | 325.27 | 362.37 |
| 1561.46 | 1597.18 | 1556.07 | 1582.44 | 1464.89 | 1648.75 |
| 134.73 | 159.44 | 132.13 | 126.37 | 154.1 | 150.12 |
| 9273.58 | 7048.65 | 6414.44 | 10117.17 | 8870.22 | 9352.54 |
| 1 | 1 | 1 | 75.06 | 48.99 | 71.33 |
| 2.07 | 24.82 | 12.88 | 25.52 | 29.47 | 24.7 |
| 472.23 | 479.64 | 415.68 | 356.36 | 430.37 | 434.73 |
| 1733.82 | 1634.61 | 1725.67 | 2026.51 | 2169.09 | 2045.92 |
| 154.58 | 140.32 | 167.46 | 174.13 | 148.09 | 148.51 |
| 2.97 | 1 | 1 | 83.9 | 36.98 | 69.72 |
| 455.09 | 430.26 | 392.71 | 726.13 | 955.89 | 632.51 |
| 242.11 | 237.5 | 208.09 | 296.21 | 215.66 | 298.05 |
| 148.26 | 104.47 | 153.33 | 99.83 | 83.53 | 196.75 |
| 1 | 1 | 1 | 36.13 | 6.95 | 31.13 |
| 204.21 | 191.3 | 142.73 | 719.06 | 550.49 | 465.28 |
| 30.95 | 15.26 | 6.69 | 44.98 | 24.97 | 40.78 |
| 20.12 | 31.99 | 9.34 | 27.29 | 9.95 | 42.38 |
| 1 | 1 | 1 | 1.77 | 1.5 | 1.61 |
| 1 | 1 | 1 | 1.77 | 1.5 | 1.61 |
| 1610.19 | 1071.47 | 1073.77 | 894.21 | 919.85 | 1176 |
| 2435.91 | 1762.86 | 1746.87 | 1743.44 | 1709.64 | 1979.99 |
| 1951.31 | 1946.06 | 1980.96 | 1585.98 | 1621.05 | 2021.8 |
| 49 | 54.29 | 32.31 | 250.21 | 167.61 | 196.75 |
| 64.34 | 68.63 | 42.91 | 82.13 | 65.51 | 64.9 |
| 76.07 | 47.12 | 15.53 | 96.29 | 70.01 | 53.64 |
| 137.43 | 39.16 | 27.89 | 103.37 | 68.51 | 37.56 |
| 2147.13 | 1142.36 | 1226.59 | 579.29 | 585.02 | 402.57 |
| 1 | 1 | 1 | 1.77 | 1.5 | 1.61 |
| 1 | 1 | 1 | 1.77 | 1.5 | 1.61 |
| 1 | 4.91 | 1 | 1.77 | 1.5 | 5.4 |
| 1 | 1 | 1 | 1.77 | 1.5 | 1.61 |
| 3.87 | 5.7 | 1 | 1.77 | 3.95 | 29.52 |
| 2.07 | 4.11 | 1 | 6.06 | 6.95 | 8.62 |
| 1 | 1 | 1 | 1.77 | 1.5 | 1.61 |
| 1 | 1 | 1 | 1.77 | 1.5 | 15.05 |
| 57.12 | 34.38 | 23.48 | 55.6 | 47.49 | 43.99 |
| 142.85 | 149.88 | 110.93 | 48.52 | 94.04 | 61.68 |
| 184.36 | 161.03 | 157.74 | 149.37 | 191.63 | 135.65 |

| 262.87 | 332.28 | 273.46 | 310.36 | 254.7 | 306.09 |
| --- | --- | --- | --- | --- | --- |
| 71.56 | 48.72 | 40.26 | 90.98 | 88.03 | 95.45 |
| 10.19 | 16.86 | 1 | 4.29 | 12.96 | 21.48 |
| 1 | 16.06 | 1 | 319.21 | 212.65 | 204.79 |
| 41.78 | 35.97 | 6.69 | 922.52 | 594.03 | 412.22 |
| 49 | 23.23 | 22.59 | 204.21 | 253.19 | 248.2 |
| 51.7 | 12.08 | 12.88 | 4636.12 | 2097.02 | 2758.25 |
| 1 | 1 | 1 | 39.67 | 1.5 | 3.79 |
| 2.97 | 4.11 | 1 | 181.21 | 55 | 98.66 |
| 1 | 1 | 1 | 1.77 | 1.5 | 1.61 |
| 142.85 | 161.82 | 135.66 | 232.52 | 185.63 | 191.93 |
| 1 | 1 | 1 | 1.77 | 1.5 | 1.61 |
| 57.12 | 29.6 | 19.06 | 409.44 | 89.53 | 225.69 |
| 10.19 | 6.5 | 1 | 151.13 | 11.46 | 66.5 |
| 39.07 | 12.08 | 3.16 | 2141.51 | 1009.94 | 1571.56 |
| 29.14 | 28.01 | 14.64 | 1.77 | 3.95 | 3.79 |
| 169.02 | 190.5 | 162.16 | 430.67 | 199.14 | 198.36 |
| 19.22 | 4.11 | 1 | 1.77 | 1.5 | 5.4 |
| 92.31 | 67.83 | 44.68 | 1150.75 | 726.16 | 1021.64 |
| 1 | 4.91 | 1 | 1.77 | 1.5 | 1.61 |
| 2.07 | 12.08 | 1 | 9.6 | 9.95 | 11.83 |
| 1 | 1 | 1 | 1.77 | 1.5 | 1.61 |
| 1 | 1 | 1 | 1.77 | 1.5 | 1.61 |
| 35.46 | 32.79 | 45.56 | 41.44 | 74.52 | 31.13 |
| 1 | 1 | 1 | 1.77 | 1.5 | 1.61 |
| 1 | 1 | 1 | 4.29 | 1.5 | 1.61 |
| 1 | 1 | 1 | 1.77 | 1.5 | 1.61 |
| 15.61 | 1 | 1 | 9.6 | 14.46 | 1.61 |
| 7.48 | 8.09 | 1 | 1.77 | 3.95 | 1.61 |
| 17.41 | 13.67 | 4.04 | 119.29 | 61 | 146.9 |
| 104.04 | 51.9 | 32.31 | 64.44 | 101.54 | 39.17 |
| 60.73 | 39.96 | 20.83 | 1338.28 | 652.59 | 1328.76 |
| 1 | 1 | 1 | 1.77 | 1.5 | 1.61 |
| 12 | 27.21 | 10.23 | 2.52 | 8.45 | 7.01 |
| 2016.28 | 2006.6 | 1989.79 | 1877.9 | 2343.26 | 2184.2 |
| 1 | 1 | 1 | 1.77 | 1.5 | 1.61 |
| 67.04 | 63.05 | 43.79 | 39.67 | 41.49 | 48.82 |
| 1 | 1 | 1 | 1.77 | 1.5 | 1.61 |
| 1 | 1 | 1 | 1.77 | 1.5 | 1.61 |
| 9.29 | 13.67 | 1 | 1.77 | 1.5 | 1.61 |
| 5.68 | 1 | 1 | 1.77 | 1.5 | 1.61 |
| 1 | 1 | 1 | 25.52 | 24.97 | 40.78 |
| 2.07 | 2.52 | 1 | 1.77 | 1.5 | 1.61 |
| 194.29 | 210.41 | 207.21 | 230.75 | 350.79 | 256.24 |
| 1 | 1 | 1 | 1.77 | 1.5 | 1.61 |
| 89.6 | 59.07 | 63.23 | 750.9 | 449.89 | 688.79 |
| 1 | 1 | 1 | 1.77 | 1.5 | 1.61 |
| 1786.16 | 2011.38 | 1857.29 | 1509.9 | 1311.74 | 2195.46 |

| 23.73 | 31.99 | 5.81 | 9.6 | 14.46 | 19.87 |
| --- | --- | --- | --- | --- | --- |
| 12 | 7.3 | 1 | 18.44 | 12.96 | 16.66 |
| 1.17 | 1 | 1 | 1.77 | 1.5 | 1.61 |
| 72.46 | 83.76 | 78.24 | 96.29 | 106.05 | 142.08 |
| 67.04 | 48.72 | 78.24 | 87.44 | 29.47 | 101.88 |
| 9.29 | 3.31 | 1 | 128.13 | 48.99 | 140.47 |
| 1 | 1 | 1 | 1.77 | 1.5 | 1.61 |
| 248.43 | 193.69 | 167.46 | 703.13 | 745.68 | 471.71 |
| 1 | 1 | 1 | 1.77 | 1.5 | 1.61 |
| 56.22 | 47.12 | 19.94 | 1.77 | 6.95 | 11.83 |
| 3.87 | 1 | 1 | 4.29 | 1.5 | 8.62 |
| 6.58 | 12.87 | 1 | 11.37 | 1.5 | 1.61 |
| 100.43 | 114.83 | 100.33 | 138.75 | 139.08 | 180.67 |
| 12.9 | 14.47 | 5.81 | 11.37 | 6.95 | 7.01 |
| 7.48 | 9.69 | 1 | 4.29 | 3.95 | 5.4 |
| 1 | 1 | 1 | 1.77 | 1.5 | 2.18 |
| 1 | 8.89 | 1 | 9.6 | 1.5 | 7.01 |
| 827.79 | 444.59 | 307.91 | 388.21 | 406.35 | 489.4 |
| 1 | 1 | 1 | 1.77 | 1.5 | 1.61 |
| 1 | 8.89 | 1 | 1.77 | 1.5 | 1.61 |
| 12 | 3.31 | 4.04 | 34.37 | 21.97 | 63.29 |
| 15839.63 | 13956.99 | 14881.26 | 11106.17 | 12428.75 | 11700.17 |
| 430.72 | 395.21 | 403.31 | 609.36 | 542.98 | 494.22 |
| 1143.64 | 970.31 | 1030.48 | 876.52 | 919.85 | 984.66 |
| 41.78 | 39.96 | 40.26 | 94.52 | 98.54 | 148.51 |
| 1 | 1 | 1 | 1.77 | 1.5 | 1.61 |
| 1 | 1 | 1 | 1.77 | 1.5 | 1.61 |
| 2120.06 | 1688.78 | 1776.02 | 1603.67 | 1559.49 | 1418.81 |
| 1 | 1 | 1 | 1.77 | 1.5 | 1.61 |
| 131.12 | 122.79 | 118.88 | 80.37 | 100.04 | 117.96 |
| 130.21 | 148.28 | 134.78 | 205.98 | 178.12 | 227.3 |
| 122.99 | 118.81 | 86.19 | 439.52 | 344.79 | 503.87 |
| 295.36 | 292.46 | 330 | 2509.51 | 2595.51 | 2549.21 |
| 10.19 | 12.87 | 4.04 | 20.21 | 58 | 21.48 |
| 118.48 | 154.66 | 112.69 | 883.59 | 694.63 | 883.35 |
| 1 | 1 | 1 | 121.06 | 92.54 | 60.07 |
| 1 | 1 | 1 | 1.77 | 1.5 | 1.61 |
| 735.74 | 524.25 | 431.58 | 492.59 | 536.98 | 568.19 |
| 1 | 1 | 1 | 1.77 | 1.5 | 1.61 |
| 206.92 | 185.72 | 174.53 | 184.75 | 238.18 | 89.02 |
| 1052.49 | 1002.96 | 1013.7 | 1212.67 | 1142.07 | 1073.09 |
| 1 | 1 | 1 | 1.77 | 1.5 | 1.61 |
| 33.65 | 37.57 | 18.18 | 43.21 | 14.46 | 48.82 |
| 1 | 1 | 1 | 1.77 | 1.5 | 1.61 |
| 1 | 1 | 1 | 60.9 | 53.5 | 53.64 |
| 65.24 | 50.31 | 35.84 | 167.06 | 104.55 | 180.67 |
| 26825.75 | 27105.36 | 26089.97 | 27271.57 | 27882.04 | 29831.65 |
| 49 | 58.28 | 30.54 | 43.21 | 41.49 | 64.9 |

| 1 | 1 | 1 | 1.77 | 1.5 | 1.61 |
| --- | --- | --- | --- | --- | --- |
| 185.26 | 212.01 | 161.28 | 179.44 | 208.15 | 217.65 |
| 808.84 | 752.06 | 799.93 | 747.36 | 700.64 | 823.86 |
| 768.23 | 609.48 | 627.68 | 1065.82 | 994.93 | 1015.21 |
| 936.98 | 850.83 | 898.87 | 420.06 | 407.85 | 479.75 |
| 1 | 1 | 1 | 1.77 | 1.5 | 1.61 |
| 2.97 | 1 | 1 | 53.83 | 35.48 | 58.46 |
| 28.24 | 43.94 | 28.78 | 29.06 | 18.96 | 37.56 |
| 1 | 1 | 1 | 1.77 | 1.5 | 1.61 |
| 9059.71 | 9183.36 | 8305.68 | 9022.02 | 9580.43 | 9432.93 |
| 230.38 | 210.41 | 167.46 | 398.83 | 331.27 | 204.79 |
| 1 | 1 | 1 | 1.77 | 1.5 | 1.61 |
| 3952.89 | 2964.03 | 2823.67 | 1838.97 | 1963.39 | 2025.01 |
| 420.79 | 437.43 | 430.7 | 474.9 | 506.95 | 536.03 |
| 1829.48 | 2244.76 | 2153.21 | 4322.96 | 3508.42 | 4919.36 |
| 715.89 | 589.56 | 574.68 | 340.44 | 385.33 | 399.35 |
| 1 | 1 | 1 | 1145.44 | 591.03 | 426.69 |
| 29.14 | 13.67 | 4.04 | 4078.81 | 1643.57 | 3422.34 |
| 1 | 1 | 1 | 27.29 | 24.97 | 48.82 |
| 1808.72 | 1564.52 | 1369.69 | 582.83 | 691.63 | 701.65 |
| 1 | 1 | 1 | 1.77 | 8.45 | 1.61 |
| 1 | 1 | 1 | 1.77 | 1.5 | 1.61 |
| 1 | 1 | 1 | 7.83 | 1.5 | 1.61 |
| 4.78 | 10.48 | 1 | 117.52 | 48.99 | 68.11 |
| 1 | 1 | 1 | 1.77 | 1.5 | 1.61 |
| 1 | 1 | 1 | 2.52 | 9.95 | 13.44 |
| 1 | 1 | 1 | 1.77 | 1.5 | 1.61 |
| 45.39 | 34.38 | 28.78 | 2.52 | 32.48 | 23.09 |
| 1 | 1 | 1 | 1.77 | 1.5 | 1.61 |
| 2.07 | 1 | 1 | 1.77 | 1.5 | 1.61 |
| 121.19 | 54.29 | 22.59 | 230.75 | 146.59 | 225.69 |
| 286.33 | 235.11 | 166.58 | 1083.52 | 690.13 | 938.02 |
| 46.29 | 58.28 | 64.11 | 18.44 | 5.45 | 1.61 |
| 1 | 1 | 1 | 1.77 | 1.5 | 1.61 |
| 43.58 | 39.96 | 11.11 | 18.44 | 3.95 | 7.01 |
| 57.12 | 55.09 | 19.94 | 220.13 | 133.08 | 179.06 |
| 222.26 | 196.87 | 162.16 | 138.75 | 112.06 | 122.78 |
| 224.07 | 180.15 | 167.46 | 101.6 | 83.53 | 156.55 |
| 84.19 | 56.68 | 55.28 | 20.21 | 71.51 | 19.87 |
| 321.53 | 327.5 | 278.76 | 269.67 | 286.23 | 318.96 |
| 30.04 | 55.89 | 27.01 | 37.9 | 59.5 | 61.68 |
| 33.65 | 26.41 | 13.76 | 188.29 | 188.63 | 211.22 |
| 1 | 4.11 | 1 | 1.77 | 1.5 | 1.61 |
| 1 | 1 | 1 | 1.77 | 1.5 | 1.61 |
| 1 | 1 | 1 | 1.77 | 1.5 | 1.61 |
| 122.09 | 100.49 | 87.96 | 151.13 | 142.08 | 198.36 |
| 130.21 | 92.53 | 78.24 | 221.9 | 221.66 | 219.26 |
| 447.87 | 302.81 | 261.98 | 1065.82 | 1047.48 | 875.31 |

| 208.73 | 165.81 | 136.54 | 317.44 | 317.76 | 409 |
| --- | --- | --- | --- | --- | --- |
| 23.73 | 8.89 | 1 | 168.83 | 124.07 | 193.53 |
| 372.06 | 316.35 | 249.61 | 151.13 | 238.18 | 174.24 |
| 152.77 | 212.01 | 184.24 | 688.98 | 715.65 | 757.93 |
| 654.52 | 564.87 | 395.36 | 734.98 | 768.2 | 788.48 |
| 1 | 1 | 1 | 1.77 | 1.5 | 1.61 |
| 1 | 1 | 1 | 1.77 | 1.5 | 1.61 |
| 674.38 | 756.84 | 698.35 | 517.36 | 604.54 | 683.96 |
| 3608.16 | 3694.45 | 3576.27 | 3647.12 | 3832.74 | 4099.29 |
| 58.92 | 54.29 | 44.68 | 89.21 | 65.51 | 68.11 |
| 1 | 1 | 1 | 1.77 | 1.5 | 1.61 |
| 32.75 | 12.87 | 8.46 | 37.9 | 17.46 | 18.26 |
| 69.75 | 89.34 | 88.84 | 82.13 | 95.54 | 126 |
| 218.65 | 231.12 | 193.96 | 175.9 | 182.62 | 201.57 |
| 6.58 | 5.7 | 1 | 6.06 | 1.5 | 19.87 |
| 1 | 1 | 1 | 1.77 | 1.5 | 1.61 |
| 1 | 1 | 1 | 1.77 | 1.5 | 16.66 |
| 1 | 1 | 1 | 1.77 | 1.5 | 15.05 |
| 416.28 | 231.12 | 135.66 | 90.98 | 100.04 | 158.16 |
| 164.51 | 98.9 | 50.86 | 50.29 | 45.99 | 82.58 |
| 246.63 | 264.58 | 229.29 | 151.13 | 166.11 | 241.77 |
| 16.51 | 10.48 | 1 | 4.29 | 1.5 | 1.61 |
| 1 | 1 | 1 | 1.77 | 1.5 | 1.61 |
| 70.65 | 63.05 | 9.34 | 34.37 | 15.96 | 31.13 |
| 415.38 | 315.56 | 323.81 | 734.98 | 618.06 | 629.29 |
| 4.78 | 16.06 | 4.04 | 13.13 | 1.5 | 7.01 |
| 1 | 1 | 1 | 1.77 | 1.5 | 1.61 |
| 1 | 1 | 1 | 1.77 | 1.5 | 1.61 |
| 1 | 1 | 1 | 1.77 | 1.5 | 1.61 |
| 1 | 1 | 1 | 1.77 | 1.5 | 1.61 |
| 1 | 1 | 1 | 1.77 | 1.5 | 1.61 |
| 315.21 | 238.29 | 177.18 | 190.06 | 184.13 | 208.01 |
| 1202.3 | 1120.85 | 1096.74 | 1440.9 | 1560.99 | 1425.24 |
| 1 | 2.52 | 1 | 4.29 | 1.5 | 18.26 |
| 54.41 | 26.41 | 4.93 | 506.75 | 320.76 | 454.02 |
| 131.12 | 117.22 | 132.13 | 52.06 | 62.51 | 56.86 |
| 6.58 | 21.64 | 13.76 | 4.29 | 9.95 | 21.48 |
| 509.23 | 549.74 | 495.18 | 927.82 | 687.12 | 1095.61 |
| 1 | 1 | 1 | 1.77 | 1.5 | 5.4 |
| 272.8 | 176.96 | 148.03 | 230.75 | 223.17 | 294.84 |
| 25.53 | 16.06 | 1 | 133.44 | 64.01 | 148.51 |
| 154.58 | 133.95 | 73.83 | 565.13 | 532.47 | 491.01 |
| 286.33 | 231.12 | 195.73 | 853.52 | 621.06 | 965.36 |
| 395.53 | 407.16 | 372.4 | 1292.28 | 1080.51 | 1841.7 |
| 88.7 | 94.12 | 59.69 | 1000.36 | 855.29 | 669.49 |
| 1 | 1 | 1 | 1.77 | 1.5 | 1.61 |
| 1 | 1 | 1 | 1.77 | 1.5 | 1.61 |
| 1425.19 | 1531.86 | 1518.97 | 1294.05 | 1379.31 | 1404.34 |

| 440.65 | 398.4 | 363.56 | 466.06 | 440.88 | 471.71 |
| --- | --- | --- | --- | --- | --- |
| 1 | 1 | 1 | 1.77 | 1.5 | 1.61 |
| 1 | 1 | 1 | 1.77 | 1.5 | 1.61 |
| 570.6 | 505.13 | 510.2 | 540.36 | 579.02 | 589.09 |
| 746.57 | 799.85 | 790.22 | 876.52 | 858.29 | 860.84 |
| 298.07 | 298.03 | 319.4 | 448.36 | 412.35 | 397.75 |
| 514.65 | 533.81 | 550.83 | 736.75 | 757.69 | 695.22 |
| 421.7 | 403.17 | 369.75 | 393.52 | 389.83 | 333.43 |
| 23.73 | 27.21 | 1 | 126.37 | 74.52 | 100.27 |
| 338.67 | 328.3 | 319.4 | 328.06 | 317.76 | 363.98 |
| 16.51 | 10.48 | 7.58 | 16.67 | 1.5 | 8.62 |
| 114.87 | 140.32 | 117.11 | 165.29 | 220.16 | 188.71 |
| 1 | 1 | 1 | 1.77 | 1.5 | 1.61 |
| 257.46 | 261.39 | 210.74 | 260.83 | 241.18 | 261.07 |
| 1 | 1 | 1 | 1.77 | 1.5 | 1.61 |
| 123.9 | 80.58 | 95.91 | 46.75 | 53.5 | 61.68 |
| 359.43 | 340.25 | 304.38 | 308.6 | 280.22 | 330.21 |
| 9.29 | 16.86 | 1.39 | 7.83 | 17.46 | 8.62 |
| 13.8 | 21.64 | 15.53 | 2.52 | 1.5 | 5.4 |
| 1 | 1 | 1 | 69.75 | 1.5 | 11.83 |
| 1 | 1 | 1 | 1.77 | 1.5 | 1.61 |
| 1 | 1 | 1 | 1.77 | 1.5 | 1.61 |
| 1 | 1 | 1 | 1.77 | 1.5 | 1.61 |
| 260.16 | 223.16 | 183.36 | 611.13 | 530.97 | 527.99 |
| 1.17 | 11.28 | 1 | 11.37 | 1.5 | 23.09 |
| 25.53 | 20.04 | 5.81 | 30.83 | 41.49 | 52.03 |
| 281.82 | 208.82 | 176.29 | 280.29 | 529.47 | 185.49 |
| 1 | 1 | 1 | 1.77 | 1.5 | 1.61 |
| 1 | 1 | 1 | 1.77 | 1.5 | 1.61 |
| 2.97 | 1 | 1 | 1.77 | 1.5 | 1.61 |
| 206.92 | 213.6 | 215.16 | 441.29 | 564 | 507.09 |
| 30.04 | 35.97 | 22.59 | 7.83 | 3.95 | 13.44 |
| 10.19 | 1 | 1 | 1.77 | 1.5 | 1.61 |
| 1434.22 | 1362.2 | 1376.75 | 1071.13 | 909.34 | 1052.19 |
| 1 | 1 | 1 | 1.77 | 1.5 | 1.61 |
| 1 | 1 | 1 | 1.77 | 1.5 | 1.61 |
| 1 | 1 | 1 | 117.52 | 95.54 | 105.1 |
| 10.19 | 8.09 | 1 | 29.06 | 1.5 | 18.26 |
| 1 | 8.89 | 1 | 87.44 | 24.97 | 56.86 |
| 156.38 | 183.33 | 92.38 | 405.9 | 509.95 | 507.09 |
| 11.09 | 12.87 | 1 | 73.29 | 65.51 | 142.08 |
| 811.54 | 887.47 | 855.58 | 595.21 | 597.04 | 568.19 |
| 1 | 7.3 | 1 | 1.77 | 1.5 | 1.61 |
| 1 | 1 | 1 | 1.77 | 11.46 | 8.62 |
| 1 | 1 | 1 | 952.59 | 409.35 | 1010.38 |
| 1 | 1 | 1 | 1.77 | 1.5 | 1.61 |
| 1 | 1 | 1 | 1.77 | 1.5 | 1.61 |
| 2.07 | 3.31 | 1 | 2.52 | 1.5 | 1.61 |

| 1.17 | 6.5 | 1 | 1.77 | 1.5 | 1.61 |
| --- | --- | --- | --- | --- | --- |
| 307.09 | 239.89 | 212.51 | 267.9 | 451.39 | 159.77 |
| 1 | 1 | 1 | 1.77 | 1.5 | 1.61 |
| 1 | 1.72 | 1 | 1.77 | 1.5 | 1.61 |
| 1 | 1 | 1 | 1.77 | 1.5 | 1.61 |
| 1 | 1 | 1 | 1.77 | 1.5 | 1.61 |
| 1 | 1 | 1 | 1.77 | 1.5 | 1.61 |
| 1 | 1.72 | 1 | 1.77 | 1.5 | 1.61 |
| 1 | 1 | 1 | 1.77 | 1.5 | 1.61 |
| 1 | 1 | 1 | 1.77 | 1.5 | 1.61 |
| 7.48 | 6.5 | 1 | 4.29 | 8.45 | 1.61 |
| 6.58 | 1 | 1 | 39.67 | 14.46 | 23.09 |
| 1 | 1 | 1 | 1.77 | 1.5 | 1.61 |
| 1743.75 | 1498.41 | 1754.82 | 1584.21 | 1404.83 | 1415.59 |
| 1 | 1 | 1 | 1.77 | 1.5 | 1.61 |
| 195.19 | 159.44 | 144.49 | 772.13 | 784.72 | 968.58 |
| 1 | 1 | 1 | 1.77 | 1.5 | 1.61 |
| 1 | 1 | 1 | 117.52 | 47.49 | 101.88 |
| 109.46 | 89.34 | 100.33 | 204.21 | 271.21 | 270.72 |
| 1000.15 | 925.7 | 914.77 | 809.29 | 688.63 | 579.45 |
| 1 | 7.3 | 1 | 1.77 | 1.5 | 1.61 |
| 6.58 | 4.11 | 1 | 1.77 | 1.5 | 1.61 |
| 1 | 1 | 1 | 1.77 | 1.5 | 1.61 |
| 645.5 | 567.26 | 529.63 | 696.06 | 759.2 | 692 |
| 577.82 | 562.48 | 503.13 | 443.06 | 544.48 | 478.14 |
| 521.87 | 534.6 | 531.4 | 1171.98 | 1003.94 | 1135.8 |
| 54.41 | 39.96 | 23.48 | 253.75 | 221.66 | 273.93 |
| 144.65 | 94.92 | 68.53 | 126.37 | 152.6 | 103.49 |
| 145.56 | 124.39 | 114.46 | 568.67 | 367.31 | 645.37 |
| 10.19 | 12.08 | 1 | 4.29 | 3.95 | 1.61 |
| 1 | 5.7 | 1 | 1.77 | 1.5 | 1.61 |
| 70.65 | 53.5 | 73.83 | 64.44 | 24.97 | 71.33 |
| 71.56 | 42.34 | 13.76 | 653.59 | 500.94 | 529.6 |
| 76.97 | 56.68 | 38.49 | 67.98 | 47.49 | 76.15 |
| 688.82 | 491.59 | 458.96 | 4289.35 | 2978.39 | 4745.7 |
| 1 | 1 | 1 | 1.77 | 1.5 | 1.61 |
| 198.8 | 259 | 156.86 | 197.13 | 193.14 | 251.42 |
| 8.39 | 11.28 | 1 | 94.52 | 74.52 | 56.86 |
| 589.55 | 681.96 | 616.2 | 466.06 | 446.89 | 510.3 |
| 12.9 | 17.65 | 1 | 43.21 | 11.46 | 29.52 |
| 187.97 | 196.87 | 171.88 | 158.21 | 149.59 | 216.05 |
| 53.51 | 25.62 | 3.16 | 1.77 | 1.5 | 5.4 |
| 1713.97 | 1715.86 | 1716.84 | 1731.05 | 1724.65 | 1761.3 |
| 44.48 | 28.01 | 19.94 | 67.98 | 80.52 | 79.37 |
| 1 | 1 | 1 | 1.77 | 1.5 | 1.61 |
| 3797.67 | 4007.49 | 3955.22 | 4022.2 | 4296.7 | 4723.19 |
| 1177.93 | 1231.57 | 1291.95 | 1302.9 | 1145.08 | 1291.78 |
| 39.97 | 32.79 | 14.64 | 75.06 | 101.54 | 97.06 |

| 1 | 1 | 1 | 1.77 | 1.5 | 1.61 |
| --- | --- | --- | --- | --- | --- |
| 1.17 | 1 | 1 | 6.06 | 1.5 | 3.79 |
| 366.65 | 371.31 | 312.33 | 370.52 | 493.43 | 392.92 |
| 1 | 1 | 1 | 1.77 | 1.5 | 1.61 |
| 1 | 1 | 1 | 1.77 | 1.5 | 1.61 |
| 1 | 1 | 1 | 1.77 | 1.5 | 1.61 |
| 1 | 1 | 1 | 1.77 | 1.5 | 1.61 |
| 1 | 1 | 1 | 1.77 | 1.5 | 1.61 |
| 1 | 1 | 1 | 1.77 | 1.5 | 1.61 |
| 1 | 1 | 1 | 1.77 | 1.5 | 1.61 |
| 1 | 1 | 1 | 1.77 | 1.5 | 1.61 |
| 1 | 1 | 1 | 1.77 | 1.5 | 1.61 |
| 1 | 5.7 | 1 | 1.77 | 1.5 | 1.61 |
| 1 | 1 | 1 | 1.77 | 1.5 | 1.61 |
| 1 | 1 | 1 | 1.77 | 1.5 | 1.61 |
| 1 | 2.52 | 1 | 1.77 | 1.5 | 1.61 |
| 149.16 | 116.42 | 123.29 | 87.44 | 82.03 | 74.54 |
| 11.09 | 20.04 | 11.99 | 50.29 | 53.5 | 43.99 |
| 41.78 | 43.14 | 17.29 | 69.75 | 55 | 64.9 |
| 186.16 | 201.65 | 163.93 | 165.29 | 158.6 | 151.73 |
| 10.19 | 13.67 | 1 | 115.75 | 55 | 42.38 |
| 1 | 7.3 | 1 | 1.77 | 1.5 | 1.61 |
| 1 | 1 | 1 | 1.77 | 1.5 | 1.61 |
| 1 | 1 | 1 | 13.13 | 11.46 | 23.09 |
| 84.19 | 35.18 | 16.41 | 181.21 | 137.58 | 175.85 |
| 344.09 | 325.11 | 397.13 | 490.83 | 457.4 | 505.48 |
| 1 | 1 | 1 | 1.77 | 1.5 | 1.61 |
| 18.31 | 12.08 | 1 | 1.77 | 1.5 | 7.01 |
| 1 | 1 | 1 | 1.77 | 1.5 | 1.61 |
| 1 | 1 | 1 | 1.77 | 1.5 | 1.61 |
| 122.99 | 102.88 | 94.14 | 262.6 | 227.67 | 225.69 |
| 1 | 1 | 1 | 1.77 | 1.5 | 1.61 |
| 735.74 | 501.15 | 516.38 | 228.98 | 280.22 | 257.85 |
| 74.26 | 78.99 | 44.68 | 92.75 | 77.52 | 124.39 |
| 181.65 | 196.87 | 185.13 | 225.44 | 176.62 | 167.81 |
| 1 | 4.11 | 1 | 1.77 | 1.5 | 1.61 |
| 539.01 | 530.62 | 512.85 | 483.75 | 478.42 | 508.7 |
| 1113.86 | 1031.64 | 1215.1 | 954.36 | 1104.54 | 936.42 |
| 8597.67 | 9552.95 | 8837.45 | 9494.41 | 8787.64 | 11415.56 |
| 63.43 | 84.56 | 73.83 | 105.13 | 94.04 | 137.25 |
| 906.3 | 864.37 | 863.53 | 1097.67 | 945.38 | 921.94 |
| 363.94 | 402.38 | 343.25 | 621.75 | 482.92 | 606.78 |
| 1 | 1 | 1 | 41.44 | 1.5 | 27.91 |
| 113.97 | 122.79 | 102.09 | 89.21 | 86.53 | 119.57 |
| 1 | 1 | 1 | 2.52 | 1.5 | 2.18 |
| 1 | 1 | 1 | 1.77 | 1.5 | 1.61 |
| 8603.99 | 7258.14 | 9534.4 | 5497.73 | 6181.06 | 5589.88 |
| 4804.78 | 4839.86 | 5192.78 | 4892.65 | 4712.61 | 5176.63 |

| 226.77 | 235.11 | 243.43 | 259.06 | 196.14 | 264.28 |
| --- | --- | --- | --- | --- | --- |
| 206.92 | 216.79 | 149.79 | 82.13 | 148.09 | 93.84 |
| 1 | 1 | 1 | 4.29 | 1.5 | 1.61 |
| 2.97 | 8.89 | 1 | 175.9 | 133.08 | 177.45 |
| 6.58 | 14.47 | 1 | 44.98 | 35.48 | 40.78 |
| 255.65 | 271.75 | 211.63 | 427.13 | 326.77 | 428.3 |
| 87.8 | 71.82 | 56.16 | 336.9 | 295.24 | 314.13 |
| 6701.67 | 6485.5 | 6358.79 | 6722.03 | 7353.72 | 7223.58 |
| 39.97 | 22.43 | 16.41 | 71.52 | 77.52 | 138.86 |
| 23.73 | 42.34 | 13.76 | 7.83 | 11.46 | 5.4 |
| 249.33 | 234.31 | 248.73 | 333.36 | 356.8 | 281.97 |
| 310.7 | 274.14 | 240.78 | 234.29 | 298.24 | 244.99 |
| 344.99 | 315.56 | 301.73 | 443.06 | 446.89 | 429.91 |
| 140.14 | 138.73 | 124.18 | 501.44 | 410.85 | 442.77 |
| 76.97 | 63.05 | 40.26 | 466.06 | 377.82 | 347.9 |
| 289.94 | 274.93 | 320.28 | 1400.21 | 1086.52 | 1372.18 |
| 139.24 | 134.74 | 130.36 | 527.98 | 385.33 | 425.08 |
| 5.68 | 1.72 | 1 | 32.6 | 1.5 | 27.91 |
| 9.29 | 7.3 | 1 | 1.77 | 1.5 | 1.61 |
| 303.48 | 309.18 | 314.98 | 356.36 | 323.76 | 283.58 |
| 236.7 | 208.02 | 209.86 | 136.98 | 166.11 | 169.41 |
| 1 | 1 | 1 | 1.77 | 1.5 | 1.61 |
| 1 | 1 | 1 | 1.77 | 1.5 | 1.61 |
| 7.48 | 15.26 | 1 | 4.29 | 1.5 | 10.22 |
| 2407.94 | 2294.14 | 2435 | 1761.13 | 1861.29 | 1738.79 |
| 132.02 | 118.81 | 91.49 | 352.83 | 301.24 | 404.18 |
| 2.97 | 2.52 | 1 | 9.6 | 17.46 | 1.61 |
| 5.68 | 4.91 | 1 | 1.77 | 1.5 | 1.61 |
| 1 | 1 | 1 | 1.77 | 1.5 | 1.61 |
| 88.7 | 101.29 | 81.78 | 129.9 | 139.08 | 111.53 |
| 505.62 | 521.06 | 488.11 | 425.36 | 437.88 | 479.75 |
| 150.07 | 142.71 | 117.11 | 119.29 | 157.1 | 177.45 |
| 690.62 | 694.71 | 630.33 | 510.29 | 514.45 | 476.54 |
| 2607.37 | 2754.54 | 2840.45 | 2068.97 | 2143.57 | 2282.29 |
| 919.84 | 1065.89 | 1007.52 | 938.44 | 871.81 | 920.34 |
| 1 | 1 | 1 | 1.77 | 1.5 | 1.61 |
| 1 | 1 | 1 | 1.77 | 1.5 | 1.61 |
| 1 | 1 | 1 | 1.77 | 1.5 | 1.61 |
| 70.65 | 51.11 | 42.03 | 7.83 | 1.5 | 8.62 |
| 35.46 | 35.97 | 31.43 | 343.98 | 239.68 | 304.48 |
| 317.92 | 329.89 | 324.7 | 489.06 | 503.94 | 442.77 |
| 141.95 | 159.44 | 142.73 | 209.52 | 157.1 | 183.89 |
| 52.61 | 44.73 | 26.13 | 27.29 | 14.46 | 16.66 |
| 35.46 | 16.06 | 85.31 | 1.77 | 1.5 | 2.18 |
| 1 | 1 | 1 | 1.77 | 1.5 | 1.61 |
| 1 | 1 | 1 | 1.77 | 1.5 | 1.61 |
| 235.8 | 190.5 | 206.33 | 228.98 | 287.73 | 198.36 |
| 1643.58 | 1888.71 | 1465.97 | 1432.05 | 1771.2 | 786.87 |

| 62.53 | 75 | 54.39 | 303.29 | 169.11 | 248.2 |
| --- | --- | --- | --- | --- | --- |
| 1 | 1 | 1 | 1.77 | 1.5 | 1.61 |
| 2576.69 | 2690.02 | 2689.4 | 3190.66 | 3362.77 | 3454.5 |
| 2657 | 2751.35 | 2606.36 | 3399.43 | 3562.47 | 3790.56 |
| 70.65 | 48.72 | 30.54 | 108.67 | 65.51 | 61.68 |
| 3924.01 | 3962.88 | 3935.79 | 3969.12 | 4107.51 | 4689.42 |
| 3430.38 | 3702.42 | 3582.46 | 3848.81 | 4142.04 | 4244.01 |
| 27.34 | 26.41 | 30.54 | 50.29 | 8.45 | 42.38 |
| 1 | 1 | 1 | 1.77 | 1.5 | 3.79 |
| 199.7 | 248.65 | 208.09 | 803.98 | 604.54 | 536.03 |
| 1789.77 | 1946.86 | 1945.63 | 1856.67 | 1532.46 | 2145.61 |
| 600.38 | 586.38 | 546.41 | 734.98 | 649.59 | 619.65 |
| 307.09 | 215.19 | 172.76 | 218.36 | 503.94 | 98.66 |
| 143.75 | 118.02 | 79.13 | 105.13 | 67.01 | 95.45 |
| 86 | 63.85 | 40.26 | 83.9 | 52 | 68.11 |
| 1037.15 | 1101.73 | 1101.15 | 1205.59 | 1326.76 | 1459.01 |
| 1 | 1 | 1 | 1.77 | 2.45 | 1.61 |
| 1 | 1 | 1 | 1.77 | 1.5 | 1.61 |
| 372.06 | 359.37 | 368.86 | 563.36 | 506.95 | 495.83 |
| 139.24 | 137.13 | 116.23 | 517.36 | 386.83 | 566.58 |
| 201.51 | 147.49 | 148.91 | 117.52 | 169.11 | 58.46 |
| 75.17 | 49.51 | 42.91 | 144.06 | 134.58 | 179.06 |
| 1 | 1 | 1 | 1.77 | 1.5 | 1.61 |
| 192.48 | 102.08 | 277 | 1138.36 | 736.67 | 2472.03 |
| 136.53 | 51.11 | 125.06 | 940.21 | 561 | 2079.68 |
| 1 | 1 | 1 | 57.37 | 26.47 | 8.62 |
| 5.68 | 4.11 | 1 | 57.37 | 33.98 | 80.98 |
| 1 | 1 | 1 | 1.77 | 1.5 | 1.61 |
| 129.31 | 87.75 | 57.93 | 161.75 | 194.64 | 138.86 |
| 1 | 1 | 1 | 1.77 | 1.5 | 1.61 |
| 36.36 | 25.62 | 23.48 | 4.29 | 2.45 | 18.26 |
| 983.01 | 987.03 | 929.78 | 1106.52 | 1161.59 | 1143.84 |
| 1 | 1 | 1 | 7.83 | 1.5 | 5.4 |
| 2.97 | 1 | 1 | 11.37 | 1.5 | 1.61 |
| 1025.42 | 1133.6 | 975.72 | 1460.36 | 1313.24 | 1611.76 |
| 1615.61 | 1460.97 | 1464.2 | 1237.44 | 1344.78 | 1040.93 |
| 30.04 | 39.96 | 32.31 | 105.13 | 94.04 | 121.17 |
| 12 | 16.86 | 1 | 306.83 | 197.64 | 372.02 |
| 1 | 1 | 1 | 2.52 | 1.5 | 1.61 |
| 942.4 | 1093.77 | 1019 | 1313.51 | 1281.71 | 1537.8 |
| 564.28 | 486.01 | 428.05 | 1051.67 | 781.72 | 687.18 |
| 365.75 | 262.19 | 246.96 | 736.75 | 678.12 | 531.21 |
| 1311.49 | 1180.59 | 1284 | 3758.58 | 3224.64 | 3128.08 |
| 1 | 1.72 | 1 | 1.77 | 1.5 | 13.44 |
| 59.82 | 43.14 | 34.08 | 136.98 | 94.04 | 109.92 |
| 927.06 | 797.46 | 763.72 | 782.75 | 862.8 | 642.16 |
| 238.51 | 247.05 | 254.03 | 204.21 | 161.6 | 217.65 |
| 52.61 | 51.9 | 42.03 | 78.6 | 39.98 | 56.86 |

| 23.73 | 16.06 | 1 | 23.75 | 27.97 | 42.38 |
| --- | --- | --- | --- | --- | --- |
| 129.31 | 108.46 | 104.74 | 115.75 | 146.59 | 114.74 |
| 115.78 | 73.41 | 59.69 | 600.52 | 379.32 | 593.92 |
| 243.92 | 221.56 | 168.34 | 315.67 | 326.77 | 322.17 |
| 562.48 | 602.31 | 538.46 | 844.67 | 856.79 | 933.2 |
| 1 | 1 | 1 | 1.77 | 1.5 | 1.61 |
| 6527.51 | 7145.83 | 6194.49 | 6458.42 | 7262.13 | 7620.75 |
| 60.73 | 51.11 | 46.44 | 44.98 | 17.46 | 23.09 |
| 3295.02 | 3183.08 | 3086.9 | 3450.74 | 3301.21 | 4451.44 |
| 206.02 | 171.38 | 182.48 | 152.9 | 139.08 | 180.67 |
| 178.95 | 237.5 | 184.24 | 121.06 | 107.55 | 105.1 |
| 295.36 | 329.1 | 315.86 | 421.83 | 497.94 | 314.13 |
| 133.82 | 113.24 | 80.89 | 121.06 | 133.08 | 169.41 |
| 687.01 | 664.44 | 718.67 | 306.83 | 374.82 | 497.44 |
| 491.18 | 387.24 | 341.48 | 497.9 | 503.94 | 508.7 |
| 6857.79 | 7313.9 | 7265.98 | 7992.34 | 7652.52 | 9244.8 |
| 1 | 1 | 1 | 1.77 | 1.5 | 1.61 |
| 44.48 | 31.99 | 26.13 | 108.67 | 74.52 | 74.54 |
| 22.83 | 11.28 | 1 | 55.6 | 42.99 | 32.74 |
| 39.97 | 16.86 | 6.69 | 273.21 | 185.63 | 265.89 |
| 136.53 | 148.28 | 129.48 | 267.9 | 166.11 | 203.18 |
| 57.12 | 55.09 | 45.56 | 73.29 | 74.52 | 60.07 |
| 1 | 5.7 | 1 | 1.77 | 1.5 | 1.61 |
| 1 | 1 | 1 | 7.83 | 1.5 | 13.44 |
| 34.56 | 18.45 | 4.04 | 9.6 | 1.5 | 35.95 |
| 4.78 | 2.52 | 1 | 34.37 | 30.97 | 29.52 |
| 1 | 1 | 1 | 2.52 | 1.5 | 1.61 |
| 60.73 | 85.36 | 48.21 | 329.83 | 161.6 | 248.2 |
| 1017.3 | 981.46 | 1183.3 | 517.36 | 512.95 | 510.3 |
| 269.19 | 240.68 | 216.04 | 122.83 | 142.08 | 138.86 |
| 12 | 7.3 | 1 | 1.77 | 1.5 | 1.61 |
| 1 | 1 | 1 | 1.77 | 1.5 | 1.61 |
| 16.51 | 13.67 | 4.93 | 41.44 | 36.98 | 40.78 |
| 1 | 8.89 | 1 | 1.77 | 1.5 | 1.61 |
| 25.53 | 26.41 | 10.23 | 62.67 | 44.49 | 82.58 |
| 29.14 | 25.62 | 9.34 | 23.75 | 14.46 | 32.74 |
| 16.51 | 16.86 | 1 | 13.13 | 6.95 | 3.79 |
| 1 | 1 | 1 | 1.77 | 1.5 | 1.61 |
| 65.24 | 56.68 | 33.19 | 122.83 | 92.54 | 232.12 |
| 1 | 1 | 1 | 29.06 | 5.45 | 16.66 |
| 178.95 | 177.76 | 152.44 | 113.98 | 134.58 | 121.17 |
| 20.12 | 26.41 | 7.58 | 4.29 | 1.5 | 16.66 |
| 1 | 1 | 1 | 1.77 | 1.5 | 1.61 |
| 1 | 1 | 1 | 1.77 | 1.5 | 1.61 |
| 1 | 1 | 1 | 1.77 | 1.5 | 1.61 |
| 1 | 1 | 1 | 1.77 | 1.5 | 1.61 |
| 3089.27 | 3265.12 | 3265.34 | 3905.43 | 4118.02 | 4147.53 |
| 6.58 | 10.48 | 1 | 1.77 | 6.95 | 7.01 |

| 39.07 | 37.57 | 21.71 | 105.13 | 74.52 | 87.41 |
| --- | --- | --- | --- | --- | --- |
| 523.67 | 585.58 | 572.91 | 402.36 | 460.4 | 465.28 |
| 48.09 | 50.31 | 34.96 | 32.6 | 36.98 | 23.09 |
| 67.95 | 82.17 | 64.11 | 21.98 | 26.47 | 42.38 |
| 455.99 | 480.44 | 489.88 | 374.06 | 466.41 | 399.35 |
| 1 | 1 | 1 | 1.77 | 3.95 | 23.09 |
| 255.65 | 200.85 | 175.41 | 18.44 | 26.47 | 53.64 |
| 133.82 | 137.13 | 172.76 | 190.06 | 161.6 | 162.98 |
| 4.78 | 1 | 1 | 9.6 | 1.5 | 3.79 |
| 72.46 | 56.68 | 50.86 | 83.9 | 61 | 61.68 |
| 1901.67 | 1449.82 | 1407.67 | 750.9 | 721.66 | 909.08 |
| 2826.66 | 2844.55 | 2808.65 | 2528.97 | 2685.6 | 2782.37 |
| 482.16 | 516.28 | 429.81 | 869.44 | 568.51 | 645.37 |
| 472.23 | 463.71 | 370.63 | 404.13 | 496.44 | 510.3 |
| 2602.86 | 2714.71 | 2728.26 | 2884.59 | 2820.74 | 2880.45 |
| 1 | 1 | 1 | 1.77 | 1.5 | 1.61 |
| 1 | 1 | 1 | 1.77 | 1.5 | 1.61 |
| 1 | 18.45 | 4.93 | 11.37 | 17.46 | 39.17 |
| 122.09 | 151.47 | 146.26 | 271.44 | 253.19 | 195.14 |
| 1793.38 | 1813.04 | 1745.99 | 1782.36 | 1891.32 | 1910.85 |
| 413.58 | 419.9 | 405.08 | 604.06 | 517.46 | 590.7 |
| 2336.64 | 2333.97 | 2316.63 | 3813.43 | 4063.97 | 4043.02 |
| 944.2 | 831.71 | 954.52 | 823.44 | 853.79 | 730.6 |
| 80100.42 | 83214.76 | 83854.24 | 71105.87 | 71353.07 | 80574.38 |
| 242.11 | 189.7 | 178.06 | 172.37 | 167.61 | 174.24 |
| 1852.04 | 1965.18 | 1979.19 | 1518.74 | 1316.25 | 1558.7 |
| 10305.96 | 10363.03 | 10548.48 | 10354.25 | 10751.59 | 11053.77 |
| 146.46 | 152.27 | 172.76 | 213.06 | 197.64 | 177.45 |
| 1770.82 | 1699.93 | 1617.91 | 2097.28 | 2287.71 | 2008.93 |
| 32825.98 | 33908.55 | 33673.45 | 17312.61 | 17466.23 | 19664.45 |
| 11481.82 | 11651.82 | 11537.82 | 12657.78 | 12721.54 | 13885.4 |
| 4024.18 | 4427.26 | 4420.75 | 4213.27 | 4640.54 | 4837.35 |
| 533.6 | 584.78 | 517.26 | 400.6 | 430.37 | 254.64 |
| 451.48 | 461.32 | 461.61 | 529.75 | 524.96 | 632.51 |

| 1Young Repop 6h | 2Young Repop 6h | 3Young Repop 6h | 1Aged Con 6h | 2Aged Con 6h | 3Aged Con 6h |
| --- | --- | --- | --- | --- | --- |
| 245.89 | 284.3 | 351.36 | 337.86 | 306.86 | 353.73 |
| 196.28 | 212.56 | 217.4 | 455.32 | 407.35 | 300.75 |
| 537.32 | 598.43 | 623.73 | 539.44 | 676.84 | 622.71 |
| 197.83 | 183.47 | 174.98 | 158.5 | 182.01 | 186.64 |
| 210.23 | 253.28 | 259.82 | 285.48 | 247.48 | 294.64 |
| 114.12 | 125.3 | 110.23 | 137.87 | 95.23 | 109.2 |
| 1.55 | 1.94 | 2.23 | 1.59 | 1.52 | 2.04 |
| 5.61 | 1.94 | 2.23 | 1.59 | 1.52 | 2.04 |
| 27897.7 | 27948.87 | 29337.32 | 28202.08 | 29469.89 | 29330.18 |
| 318.74 | 292.06 | 317.87 | 371.19 | 312.95 | 437.28 |
| 1608.48 | 1593.17 | 1474.36 | 1450.52 | 1553.83 | 1551.91 |
| 129.62 | 125.3 | 137.03 | 137.87 | 163.74 | 135.69 |
| 8395.09 | 10448.85 | 8920.11 | 9774.08 | 10495.8 | 10263.19 |
| 11.81 | 14.77 | 36.56 | 1.59 | 28.23 | 13.43 |
| 13.36 | 16.71 | 25.4 | 12.47 | 14.53 | 33.81 |
| 411.75 | 441.37 | 353.59 | 418.81 | 379.94 | 353.73 |
| 1910.76 | 1967.41 | 2010.18 | 2009.24 | 1951.22 | 2110.25 |
| 143.58 | 193.17 | 179.45 | 142.63 | 156.13 | 172.37 |
| 47.47 | 61.31 | 56.65 | 10.89 | 49.55 | 31.77 |
| 652.03 | 639.15 | 804.58 | 523.57 | 719.47 | 565.65 |
| 227.28 | 247.46 | 244.19 | 215.64 | 256.62 | 296.67 |
| 188.53 | 185.41 | 150.42 | 198.18 | 175.92 | 239.62 |
| 7.16 | 1.94 | 29.86 | 4.54 | 1.52 | 21.58 |
| 483.06 | 726.41 | 650.53 | 290.24 | 472.82 | 506.56 |
| 24.21 | 16.71 | 36.56 | 12.47 | 8.44 | 23.62 |
| 25.76 | 30.29 | 32.09 | 22 | 9.96 | 39.92 |
| 1.55 | 1.94 | 2.23 | 1.59 | 1.52 | 2.04 |
| 1.55 | 1.94 | 2.23 | 1.59 | 1.52 | 2.04 |
| 1214.74 | 1249.95 | 1059.09 | 1753.69 | 1386.35 | 1757.72 |
| 1996.02 | 2132.23 | 1782.46 | 2652.07 | 2453.66 | 2719.53 |
| 1789.85 | 1800.65 | 1590.45 | 1890.19 | 1814.19 | 1847.38 |
| 120.32 | 202.86 | 188.38 | 77.55 | 107.41 | 111.24 |
| 41.26 | 38.04 | 74.51 | 44.22 | 83.05 | 78.64 |
| 52.12 | 82.64 | 54.42 | 56.92 | 41.94 | 31.77 |
| 33.51 | 63.25 | 52.19 | 34.69 | 35.85 | 64.37 |
| 462.91 | 675.99 | 681.78 | 1353.7 | 1194.51 | 1714.93 |
| 1.55 | 1.94 | 2.23 | 1.59 | 1.52 | 2.04 |
| 1.55 | 1.94 | 2.23 | 1.59 | 1.52 | 2.04 |
| 4.06 | 1.94 | 2.23 | 1.59 | 1.52 | 2.04 |
| 2.51 | 1.94 | 2.23 | 1.59 | 1.52 | 2.04 |
| 13.36 | 10.89 | 2.23 | 1.59 | 13.01 | 3.24 |
| 10.26 | 5.08 | 18.7 | 1.59 | 5.39 | 11.39 |
| 1.55 | 1.94 | 2.23 | 1.59 | 1.52 | 2.04 |
| 1.55 | 1.94 | 2.23 | 1.59 | 6.92 | 2.04 |
| 44.36 | 39.98 | 52.19 | 50.57 | 55.64 | 90.86 |
| 69.17 | 86.52 | 36.56 | 118.82 | 121.11 | 117.35 |
| 177.68 | 158.26 | 208.47 | 117.23 | 128.72 | 168.3 |

| 239.69 | 264.91 | 221.86 | 209.29 | 252.05 | 235.54 |
| --- | --- | --- | --- | --- | --- |
| 81.57 | 146.63 | 45.49 | 83.9 | 81.52 | 101.05 |
| 36.61 | 14.77 | 38.79 | 14.06 | 11.48 | 35.84 |
| 83.12 | 90.4 | 83.44 | 7.71 | 154.61 | 52.15 |
| 338.9 | 1339.15 | 603.64 | 304.53 | 1051.39 | 734.78 |
| 197.83 | 222.25 | 208.47 | 37.87 | 121.11 | 56.22 |
| 562.12 | 1593.17 | 840.3 | 64.85 | 1314.79 | 337.43 |
| 1.55 | 10.89 | 2.23 | 1.59 | 1.52 | 2.04 |
| 53.67 | 88.46 | 41.02 | 6.12 | 8.44 | 2.04 |
| 10.26 | 1.94 | 2.23 | 1.59 | 1.52 | 2.04 |
| 204.03 | 160.2 | 168.28 | 193.42 | 218.55 | 207.01 |
| 1.55 | 1.94 | 2.23 | 1.59 | 1.52 | 2.04 |
| 41.26 | 20.59 | 36.56 | 20.41 | 60.21 | 76.6 |
| 1.55 | 1.94 | 2.23 | 2.95 | 31.28 | 2.04 |
| 514.06 | 1209.23 | 764.39 | 48.98 | 206.37 | 94.94 |
| 8.71 | 1.94 | 3.07 | 14.06 | 25.19 | 76.6 |
| 61.42 | 212.56 | 63.35 | 15.65 | 122.63 | 60.3 |
| 8.71 | 22.53 | 9.77 | 33.11 | 9.96 | 5.28 |
| 360.6 | 462.7 | 422.8 | 142.63 | 323.61 | 431.16 |
| 1.55 | 1.94 | 2.23 | 1.59 | 1.52 | 2.04 |
| 8.71 | 1.94 | 5.3 | 6.12 | 1.52 | 50.11 |
| 1.55 | 1.94 | 2.23 | 1.59 | 1.52 | 2.04 |
| 1.55 | 1.94 | 2.23 | 1.59 | 1.52 | 2.04 |
| 80.02 | 90.4 | 47.72 | 90.25 | 76.95 | 103.09 |
| 1.55 | 1.94 | 5.3 | 1.59 | 1.52 | 2.04 |
| 7.16 | 7.02 | 2.23 | 1.59 | 1.52 | 2.04 |
| 1.55 | 1.94 | 2.23 | 1.59 | 1.52 | 2.04 |
| 2.51 | 1.94 | 2.23 | 6.12 | 1.52 | 13.43 |
| 1.55 | 1.94 | 2.23 | 1.59 | 1.52 | 2.04 |
| 53.67 | 119.48 | 45.49 | 9.3 | 300.77 | 33.81 |
| 44.36 | 55.49 | 65.58 | 72.79 | 61.73 | 111.24 |
| 289.29 | 361.86 | 364.75 | 121.99 | 510.88 | 410.79 |
| 1.55 | 1.94 | 2.23 | 1.59 | 1.52 | 2.04 |
| 5.61 | 1.94 | 2.23 | 37.87 | 16.05 | 33.81 |
| 1910.76 | 2095.38 | 2077.16 | 2125.11 | 2132.41 | 2281.42 |
| 1.55 | 1.94 | 2.23 | 1.59 | 1.52 | 2.04 |
| 45.91 | 51.61 | 65.58 | 45.81 | 46.5 | 82.71 |
| 1.55 | 1.94 | 2.23 | 1.59 | 3.87 | 13.43 |
| 1.55 | 1.94 | 2.23 | 7.71 | 1.52 | 2.04 |
| 1.55 | 1.94 | 2.23 | 4.54 | 1.52 | 11.39 |
| 1.55 | 1.94 | 2.23 | 1.59 | 1.52 | 2.04 |
| 45.91 | 38.04 | 70.05 | 45.81 | 25.19 | 54.18 |
| 2.51 | 1.94 | 3.07 | 1.59 | 1.52 | 2.04 |
| 270.69 | 311.45 | 302.24 | 177.55 | 268.8 | 300.75 |
| 1.55 | 1.94 | 2.23 | 1.59 | 1.52 | 2.04 |
| 421.05 | 908.68 | 467.45 | 91.84 | 1126 | 382.26 |
| 2.51 | 1.94 | 2.23 | 1.59 | 1.52 | 11.39 |
| 1670.49 | 1232.5 | 1306.91 | 1617.19 | 1645.19 | 1441.87 |

| 11.81 | 14.77 | 5.3 | 29.93 | 13.01 | 21.58 |
| --- | --- | --- | --- | --- | --- |
| 14.91 | 14.77 | 7.53 | 22 | 1.52 | 31.77 |
| 1.55 | 1.94 | 2.23 | 1.59 | 1.52 | 3.24 |
| 135.82 | 152.45 | 152.65 | 110.88 | 93.7 | 127.54 |
| 117.22 | 166.02 | 157.12 | 82.31 | 84.57 | 107.16 |
| 66.07 | 134.99 | 72.28 | 4.54 | 204.85 | 17.5 |
| 1.55 | 1.94 | 2.23 | 1.59 | 1.52 | 2.04 |
| 275.34 | 456.88 | 474.15 | 268.02 | 335.79 | 296.67 |
| 2.51 | 1.94 | 2.23 | 1.59 | 1.52 | 2.04 |
| 13.36 | 12.83 | 2.23 | 17.24 | 17.58 | 19.54 |
| 4.06 | 1.94 | 2.23 | 7.71 | 1.52 | 3.24 |
| 5.61 | 7.02 | 12 | 1.59 | 6.92 | 15.47 |
| 129.62 | 127.24 | 94.61 | 117.23 | 115.02 | 111.24 |
| 27.31 | 1.94 | 12 | 7.71 | 5.39 | 2.04 |
| 14.91 | 7.02 | 2.23 | 1.59 | 11.48 | 5.28 |
| 1.55 | 1.94 | 2.23 | 1.59 | 1.52 | 2.04 |
| 28.86 | 7.02 | 36.56 | 4.54 | 1.52 | 15.47 |
| 340.45 | 484.03 | 373.68 | 1037.84 | 620.51 | 663.46 |
| 1.55 | 1.94 | 2.23 | 1.59 | 1.52 | 2.04 |
| 1.55 | 1.94 | 2.23 | 1.59 | 1.52 | 2.04 |
| 24.21 | 36.1 | 5.3 | 12.47 | 16.05 | 13.43 |
| 11614.78 | 12449.96 | 12740.1 | 12332.73 | 12088.4 | 12708.46 |
| 422.6 | 491.78 | 498.71 | 487.06 | 568.74 | 577.88 |
| 723.34 | 906.74 | 858.16 | 877.53 | 1173.2 | 887.61 |
| 149.78 | 129.18 | 72.28 | 69.61 | 188.1 | 135.69 |
| 1.55 | 1.94 | 2.23 | 1.59 | 1.52 | 2.04 |
| 1.55 | 1.94 | 2.23 | 1.59 | 1.52 | 2.04 |
| 1386.81 | 1529.18 | 1536.87 | 1460.05 | 1736.54 | 1663.99 |
| 1.55 | 1.94 | 2.23 | 1.59 | 1.52 | 2.04 |
| 86.22 | 36.1 | 101.3 | 74.38 | 69.34 | 131.62 |
| 205.58 | 193.17 | 152.65 | 175.96 | 220.08 | 255.92 |
| 286.19 | 402.58 | 358.05 | 141.04 | 411.92 | 471.92 |
| 2169.64 | 2215.61 | 2501.36 | 874.35 | 1843.12 | 1955.38 |
| 41.26 | 16.71 | 7.53 | 23.58 | 2.35 | 46.03 |
| 520.26 | 631.39 | 536.66 | 334.69 | 815.39 | 714.41 |
| 33.51 | 63.25 | 70.05 | 1.59 | 37.37 | 37.88 |
| 1.55 | 1.94 | 2.23 | 1.59 | 1.52 | 2.04 |
| 422.6 | 433.61 | 467.45 | 539.44 | 498.7 | 583.99 |
| 1.55 | 1.94 | 2.23 | 1.59 | 1.52 | 2.04 |
| 163.73 | 167.96 | 257.59 | 198.18 | 110.45 | 121.43 |
| 1135.68 | 1083.19 | 1199.75 | 1167.99 | 984.4 | 1109.73 |
| 1.55 | 1.94 | 2.23 | 1.59 | 1.52 | 2.04 |
| 27.31 | 12.83 | 34.33 | 7.71 | 37.37 | 62.33 |
| 1.55 | 1.94 | 2.23 | 1.59 | 1.52 | 2.04 |
| 24.21 | 18.65 | 27.63 | 1.59 | 23.67 | 27.69 |
| 92.42 | 82.64 | 74.51 | 83.9 | 51.07 | 92.9 |
| 24797.37 | 26081.55 | 25008.29 | 26154.52 | 26129.41 | 29006.18 |
| 75.37 | 51.61 | 52.19 | 52.15 | 35.85 | 70.49 |

| 1.55 | 1.94 | 2.23 | 1.59 | 1.52 | 2.04 |
| --- | --- | --- | --- | --- | --- |
| 217.98 | 200.92 | 192.84 | 166.44 | 252.05 | 231.47 |
| 693.88 | 811.73 | 594.71 | 831.5 | 932.63 | 1060.82 |
| 684.58 | 953.28 | 806.81 | 556.9 | 847.37 | 736.82 |
| 552.82 | 565.47 | 433.96 | 568.01 | 564.17 | 720.52 |
| 1.55 | 1.94 | 2.23 | 1.59 | 1.52 | 2.04 |
| 53.67 | 24.47 | 36.56 | 14.06 | 35.85 | 48.07 |
| 22.66 | 24.47 | 38.79 | 66.44 | 43.46 | 27.69 |
| 2.51 | 1.94 | 2.23 | 1.59 | 1.52 | 3.24 |
| 8646.22 | 9033.34 | 8940.2 | 9296.31 | 9139.21 | 9672.25 |
| 231.93 | 284.3 | 291.08 | 263.26 | 116.54 | 225.35 |
| 1.55 | 1.94 | 2.23 | 1.59 | 1.52 | 2.04 |
| 2625.39 | 2952.45 | 2671.04 | 3617.12 | 3224.08 | 3756.73 |
| 448.96 | 509.23 | 462.99 | 528.33 | 507.84 | 498.41 |
| 4169.35 | 4499.82 | 3925.76 | 4142.5 | 4373.61 | 4437.33 |
| 310.99 | 297.88 | 364.75 | 601.34 | 398.21 | 714.41 |
| 202.48 | 295.94 | 268.75 | 2.95 | 317.52 | 50.11 |
| 1027.17 | 3035.83 | 1103.75 | 26.76 | 4781.65 | 323.16 |
| 27.31 | 109.79 | 12 | 1.59 | 332.74 | 5.28 |
| 631.88 | 639.15 | 688.48 | 1106.09 | 746.88 | 816.29 |
| 2.51 | 8.96 | 14.23 | 14.06 | 37.37 | 111.24 |
| 1.55 | 1.94 | 2.23 | 1.59 | 1.52 | 2.04 |
| 1.55 | 1.94 | 2.23 | 1.59 | 1.52 | 9.35 |
| 160.63 | 513.11 | 210.7 | 15.65 | 299.25 | 54.18 |
| 1.55 | 1.94 | 2.23 | 1.59 | 1.52 | 2.04 |
| 4.06 | 1.94 | 7.53 | 1.59 | 1.52 | 9.35 |
| 1.55 | 1.94 | 2.23 | 1.59 | 1.52 | 2.04 |
| 24.21 | 30.29 | 20.93 | 20.41 | 1.52 | 21.58 |
| 1.55 | 1.94 | 2.23 | 1.59 | 1.52 | 2.04 |
| 1.55 | 1.94 | 2.23 | 1.59 | 1.52 | 2.04 |
| 89.32 | 88.46 | 112.47 | 22 | 111.97 | 74.56 |
| 588.47 | 945.52 | 637.13 | 236.28 | 827.57 | 451.54 |
| 11.81 | 30.29 | 20.93 | 93.42 | 54.12 | 76.6 |
| 1.55 | 1.94 | 2.23 | 1.59 | 1.52 | 2.04 |
| 2.51 | 7.02 | 14.23 | 14.06 | 25.19 | 25.66 |
| 70.72 | 76.82 | 114.7 | 48.98 | 81.52 | 80.67 |
| 83.12 | 76.82 | 101.3 | 156.91 | 159.17 | 168.3 |
| 103.27 | 117.54 | 72.28 | 137.87 | 192.67 | 198.86 |
| 50.57 | 18.65 | 41.02 | 20.41 | 28.23 | 31.77 |
| 261.39 | 218.37 | 235.26 | 285.48 | 274.89 | 323.16 |
| 27.31 | 16.71 | 49.95 | 56.92 | 14.53 | 43.99 |
| 117.22 | 129.18 | 134.79 | 69.61 | 223.12 | 133.65 |
| 1.55 | 1.94 | 2.23 | 1.59 | 1.52 | 2.04 |
| 1.55 | 1.94 | 2.23 | 1.59 | 1.52 | 2.04 |
| 1.55 | 1.94 | 2.23 | 1.59 | 1.52 | 2.04 |
| 117.22 | 127.24 | 132.56 | 148.98 | 136.33 | 162.18 |
| 261.39 | 474.33 | 306.7 | 171.2 | 274.89 | 306.86 |
| 769.84 | 916.44 | 954.16 | 899.75 | 783.42 | 1022.1 |

| 351.3 | 357.99 | 286.61 | 320.4 | 463.68 | 494.33 |
| --- | --- | --- | --- | --- | --- |
| 137.37 | 216.43 | 208.47 | 60.09 | 86.09 | 129.58 |
| 186.98 | 160.2 | 163.82 | 258.5 | 224.64 | 268.14 |
| 760.54 | 687.63 | 833.6 | 407.7 | 396.69 | 620.67 |
| 560.57 | 722.53 | 581.32 | 768.01 | 673.8 | 783.69 |
| 1.55 | 1.94 | 2.23 | 1.59 | 1.52 | 2.04 |
| 1.55 | 1.94 | 2.23 | 1.59 | 1.52 | 2.04 |
| 687.68 | 654.66 | 596.94 | 591.82 | 707.29 | 659.39 |
| 3792.66 | 3856.05 | 3758.32 | 3518.71 | 3545.34 | 3536.66 |
| 42.81 | 36.1 | 29.86 | 83.9 | 43.46 | 92.9 |
| 1.55 | 1.94 | 2.23 | 1.59 | 1.52 | 2.04 |
| 11.81 | 26.41 | 25.4 | 26.76 | 6.92 | 23.62 |
| 118.77 | 125.3 | 101.3 | 83.9 | 96.75 | 107.16 |
| 182.33 | 160.2 | 172.75 | 229.93 | 213.98 | 219.24 |
| 4.06 | 7.02 | 2.23 | 2.95 | 6.92 | 11.39 |
| 1.55 | 1.94 | 2.23 | 1.59 | 1.52 | 2.04 |
| 1.55 | 1.94 | 5.3 | 1.59 | 1.52 | 7.32 |
| 10.26 | 1.94 | 16.46 | 1.59 | 1.52 | 2.04 |
| 95.52 | 169.9 | 101.3 | 447.38 | 213.98 | 213.13 |
| 49.02 | 117.54 | 76.75 | 274.37 | 108.93 | 92.9 |
| 235.03 | 262.97 | 224.1 | 236.28 | 264.23 | 288.52 |
| 21.11 | 8.96 | 2.23 | 14.06 | 1.52 | 2.04 |
| 1.55 | 1.94 | 2.23 | 1.59 | 1.52 | 2.04 |
| 31.96 | 38.04 | 20.93 | 75.96 | 35.85 | 52.15 |
| 517.16 | 761.31 | 592.48 | 444.21 | 699.68 | 598.26 |
| 5.61 | 1.94 | 2.23 | 1.59 | 1.52 | 7.32 |
| 1.55 | 1.94 | 2.23 | 1.59 | 1.52 | 3.24 |
| 1.55 | 1.94 | 2.23 | 1.59 | 1.52 | 2.04 |
| 2.51 | 1.94 | 2.23 | 1.59 | 1.52 | 2.04 |
| 1.55 | 1.94 | 2.23 | 1.59 | 1.52 | 2.04 |
| 1.55 | 1.94 | 2.23 | 1.59 | 1.52 | 2.04 |
| 222.63 | 210.62 | 224.1 | 201.36 | 252.05 | 229.43 |
| 1348.05 | 1486.52 | 1501.15 | 1280.69 | 1527.95 | 1395.01 |
| 7.16 | 7.02 | 18.7 | 1.59 | 6.92 | 13.43 |
| 186.98 | 336.66 | 177.21 | 44.22 | 605.28 | 123.47 |
| 69.17 | 49.68 | 63.35 | 72.79 | 23.67 | 54.18 |
| 25.76 | 7.02 | 16.46 | 17.24 | 19.1 | 37.88 |
| 910.91 | 881.53 | 795.65 | 760.07 | 821.48 | 795.92 |
| 1.55 | 1.94 | 2.23 | 1.59 | 1.52 | 2.04 |
| 221.08 | 202.86 | 197.31 | 161.68 | 247.48 | 188.67 |
| 103.27 | 220.31 | 81.21 | 1.59 | 113.5 | 76.6 |
| 473.76 | 623.64 | 485.31 | 236.28 | 465.21 | 363.92 |
| 532.67 | 829.18 | 518.8 | 237.86 | 1179.29 | 469.88 |
| 955.86 | 1899.54 | 759.92 | 288.66 | 2573.95 | 649.2 |
| 549.72 | 751.62 | 721.97 | 247.39 | 267.27 | 333.35 |
| 10.26 | 1.94 | 2.23 | 1.59 | 1.52 | 2.04 |
| 1.55 | 1.94 | 2.23 | 1.59 | 1.52 | 2.04 |
| 1155.83 | 1236.38 | 1309.15 | 1207.67 | 1366.56 | 1217.72 |

| 399.35 | 377.38 | 378.15 | 406.11 | 379.94 | 390.41 |
| --- | --- | --- | --- | --- | --- |
| 1.55 | 1.94 | 2.23 | 1.59 | 1.52 | 2.04 |
| 1.55 | 1.94 | 2.23 | 1.59 | 1.52 | 2.04 |
| 591.57 | 579.04 | 563.45 | 507.7 | 580.92 | 612.52 |
| 872.15 | 906.74 | 858.16 | 875.94 | 781.9 | 816.29 |
| 355.95 | 350.23 | 391.54 | 344.21 | 340.36 | 363.92 |
| 562.12 | 652.72 | 690.71 | 598.17 | 664.66 | 610.48 |
| 342 | 348.29 | 306.7 | 334.69 | 347.97 | 339.47 |
| 55.22 | 32.22 | 72.28 | 9.3 | 51.07 | 60.3 |
| 317.19 | 342.47 | 353.59 | 356.91 | 381.47 | 353.73 |
| 10.26 | 8.96 | 3.07 | 2.95 | 1.52 | 33.81 |
| 188.53 | 152.45 | 212.93 | 139.45 | 160.7 | 164.22 |
| 1.55 | 1.94 | 2.23 | 1.59 | 1.52 | 2.04 |
| 219.53 | 259.09 | 282.15 | 228.34 | 241.39 | 247.77 |
| 1.55 | 1.94 | 2.23 | 1.59 | 1.52 | 2.04 |
| 78.47 | 67.13 | 65.58 | 93.42 | 76.95 | 94.94 |
| 255.19 | 274.61 | 219.63 | 309.29 | 253.57 | 274.26 |
| 14.91 | 12.83 | 20.93 | 9.3 | 8.44 | 3.24 |
| 19.56 | 3.14 | 5.3 | 4.54 | 8.44 | 7.32 |
| 1.55 | 1.94 | 2.23 | 1.59 | 1.52 | 2.04 |
| 1.55 | 3.14 | 2.23 | 1.59 | 1.52 | 2.04 |
| 1.55 | 1.94 | 2.23 | 1.59 | 1.52 | 2.04 |
| 1.55 | 1.94 | 2.23 | 1.59 | 1.52 | 2.04 |
| 368.35 | 493.72 | 442.89 | 291.83 | 430.19 | 463.77 |
| 11.81 | 7.02 | 5.3 | 1.59 | 34.32 | 19.54 |
| 31.96 | 43.86 | 29.86 | 7.71 | 38.89 | 35.84 |
| 276.89 | 282.36 | 420.57 | 220.4 | 331.22 | 219.24 |
| 1.55 | 1.94 | 2.23 | 1.59 | 1.52 | 2.04 |
| 1.55 | 1.94 | 2.23 | 1.59 | 1.52 | 2.04 |
| 1.55 | 1.94 | 2.23 | 2.95 | 2.35 | 2.04 |
| 335.8 | 451.06 | 456.29 | 356.91 | 539.81 | 365.96 |
| 25.76 | 12.83 | 16.46 | 28.35 | 17.58 | 23.62 |
| 7.16 | 1.94 | 2.23 | 1.59 | 1.52 | 7.32 |
| 1081.42 | 1166.57 | 1052.4 | 1074.35 | 1057.48 | 1162.71 |
| 7.16 | 1.94 | 2.23 | 1.59 | 1.52 | 2.04 |
| 1.55 | 1.94 | 2.23 | 1.59 | 1.52 | 2.04 |
| 52.12 | 57.43 | 23.16 | 1.59 | 23.67 | 3.24 |
| 4.06 | 12.83 | 2.23 | 1.59 | 35.85 | 3.24 |
| 18.01 | 14.77 | 83.44 | 15.65 | 41.94 | 64.37 |
| 417.95 | 361.86 | 371.45 | 241.04 | 284.02 | 327.24 |
| 66.07 | 51.61 | 128.1 | 25.17 | 26.71 | 105.13 |
| 565.22 | 522.81 | 467.45 | 615.63 | 692.07 | 573.8 |
| 4.06 | 1.94 | 2.23 | 1.59 | 1.52 | 2.04 |
| 1.55 | 1.94 | 7.53 | 1.59 | 1.52 | 13.43 |
| 154.43 | 330.84 | 311.17 | 4.54 | 200.28 | 247.77 |
| 1.55 | 1.94 | 2.23 | 1.59 | 1.52 | 2.04 |
| 1.55 | 1.94 | 2.23 | 1.59 | 1.52 | 2.04 |
| 2.51 | 1.94 | 2.23 | 1.59 | 1.52 | 2.04 |

| 4.06 | 5.08 | 2.23 | 10.89 | 6.92 | 7.32 |
| --- | --- | --- | --- | --- | --- |
| 273.79 | 241.64 | 300.01 | 196.59 | 259.66 | 156.07 |
| 1.55 | 1.94 | 2.23 | 1.59 | 1.52 | 2.04 |
| 1.55 | 1.94 | 2.23 | 1.59 | 1.52 | 2.04 |
| 1.55 | 1.94 | 2.23 | 1.59 | 1.52 | 2.04 |
| 1.55 | 1.94 | 2.23 | 1.59 | 1.52 | 2.04 |
| 1.55 | 1.94 | 2.23 | 1.59 | 1.52 | 2.04 |
| 10.26 | 1.94 | 2.23 | 2.95 | 1.52 | 13.43 |
| 1.55 | 1.94 | 2.23 | 1.59 | 1.52 | 2.04 |
| 4.06 | 1.94 | 3.07 | 1.59 | 1.52 | 2.04 |
| 19.56 | 10.89 | 2.23 | 4.54 | 1.52 | 13.43 |
| 30.41 | 32.22 | 25.4 | 26.76 | 23.67 | 56.22 |
| 1.55 | 1.94 | 2.23 | 1.59 | 1.52 | 2.04 |
| 1160.48 | 1515.61 | 1222.07 | 1855.27 | 1967.97 | 2077.64 |
| 1.55 | 1.94 | 2.23 | 1.59 | 1.52 | 2.04 |
| 726.44 | 743.86 | 715.27 | 469.6 | 906.75 | 889.65 |
| 1.55 | 1.94 | 2.23 | 1.59 | 1.52 | 2.04 |
| 30.41 | 32.22 | 38.79 | 1.59 | 122.63 | 19.54 |
| 276.89 | 226.13 | 230.8 | 131.52 | 249 | 241.65 |
| 498.56 | 621.7 | 632.67 | 545.79 | 596.15 | 608.44 |
| 1.55 | 1.94 | 2.23 | 1.59 | 1.52 | 2.04 |
| 1.55 | 1.94 | 2.23 | 14.06 | 1.52 | 2.04 |
| 1.55 | 1.94 | 2.23 | 1.59 | 1.52 | 2.04 |
| 692.33 | 627.52 | 717.5 | 664.83 | 649.44 | 791.84 |
| 500.11 | 439.43 | 487.55 | 460.08 | 500.23 | 455.62 |
| 869.05 | 914.5 | 931.83 | 718.8 | 1045.3 | 956.9 |
| 142.02 | 140.81 | 139.26 | 44.22 | 140.9 | 141.81 |
| 128.07 | 115.6 | 139.26 | 125.17 | 70.86 | 115.32 |
| 292.39 | 577.1 | 409.4 | 126.76 | 425.62 | 257.96 |
| 10.26 | 5.08 | 5.3 | 15.65 | 1.52 | 2.04 |
| 1.55 | 1.94 | 2.23 | 1.59 | 1.52 | 2.04 |
| 62.97 | 34.16 | 70.05 | 45.81 | 52.59 | 62.33 |
| 467.56 | 732.22 | 483.08 | 136.28 | 454.55 | 394.48 |
| 47.47 | 98.15 | 87.91 | 55.33 | 54.12 | 46.03 |
| 2589.73 | 4453.28 | 2811.69 | 698.17 | 3664.1 | 1517.27 |
| 1.55 | 1.94 | 2.23 | 1.59 | 1.52 | 23.62 |
| 210.23 | 173.78 | 197.31 | 212.47 | 156.13 | 178.48 |
| 36.61 | 45.8 | 54.42 | 28.35 | 25.19 | 33.81 |
| 503.21 | 472.39 | 505.41 | 577.54 | 541.33 | 547.31 |
| 10.26 | 12.83 | 9.77 | 2.95 | 11.48 | 17.5 |
| 152.88 | 208.68 | 197.31 | 204.53 | 209.42 | 241.65 |
| 7.16 | 1.94 | 2.23 | 6.12 | 6.92 | 7.32 |
| 1574.38 | 1628.07 | 1670.83 | 1704.48 | 1645.19 | 1778.1 |
| 76.92 | 74.88 | 105.77 | 95.01 | 130.24 | 168.3 |
| 1.55 | 1.94 | 2.23 | 1.59 | 1.52 | 2.04 |
| 4049.99 | 4189.57 | 3800.74 | 3790.13 | 4270.07 | 4003.29 |
| 993.06 | 1153 | 1371.66 | 1102.92 | 1334.59 | 1199.39 |
| 83.12 | 80.7 | 92.37 | 101.36 | 95.23 | 151.99 |

| 1.55 | 1.94 | 2.23 | 1.59 | 1.52 | 2.04 |
| --- | --- | --- | --- | --- | --- |
| 2.51 | 1.94 | 2.23 | 1.59 | 1.52 | 5.28 |
| 433.46 | 315.33 | 351.36 | 325.16 | 393.65 | 319.09 |
| 1.55 | 1.94 | 2.23 | 1.59 | 1.52 | 2.04 |
| 1.55 | 1.94 | 2.23 | 1.59 | 1.52 | 2.04 |
| 1.55 | 1.94 | 2.23 | 1.59 | 1.52 | 2.04 |
| 1.55 | 1.94 | 2.23 | 1.59 | 1.52 | 2.04 |
| 1.55 | 1.94 | 2.23 | 1.59 | 1.52 | 2.04 |
| 1.55 | 1.94 | 2.23 | 1.59 | 1.52 | 2.04 |
| 1.55 | 1.94 | 2.23 | 1.59 | 1.52 | 2.04 |
| 1.55 | 1.94 | 2.23 | 1.59 | 1.52 | 2.04 |
| 1.55 | 1.94 | 2.23 | 1.59 | 1.52 | 2.04 |
| 1.55 | 1.94 | 2.23 | 1.59 | 1.52 | 2.04 |
| 1.55 | 1.94 | 2.23 | 1.59 | 1.52 | 2.04 |
| 2.51 | 1.94 | 2.23 | 1.59 | 1.52 | 2.04 |
| 2.51 | 1.94 | 2.23 | 1.59 | 1.52 | 2.04 |
| 112.57 | 121.42 | 63.35 | 136.28 | 102.84 | 162.18 |
| 42.81 | 39.98 | 47.72 | 15.65 | 44.98 | 27.69 |
| 36.61 | 45.8 | 27.63 | 42.63 | 52.59 | 72.52 |
| 115.67 | 166.02 | 132.56 | 201.36 | 146.99 | 188.67 |
| 4.06 | 20.59 | 14.23 | 15.65 | 32.8 | 23.62 |
| 4.06 | 1.94 | 2.23 | 9.3 | 1.52 | 2.04 |
| 1.55 | 1.94 | 2.23 | 1.59 | 1.52 | 2.04 |
| 1.55 | 1.94 | 9.77 | 1.59 | 1.52 | 2.04 |
| 72.27 | 78.76 | 103.54 | 72.79 | 192.67 | 239.62 |
| 390.05 | 439.43 | 351.36 | 283.89 | 602.24 | 492.29 |
| 1.55 | 1.94 | 2.23 | 1.59 | 2.35 | 9.35 |
| 13.36 | 5.08 | 5.3 | 15.65 | 1.52 | 3.24 |
| 1.55 | 1.94 | 2.23 | 1.59 | 1.52 | 3.24 |
| 1.55 | 1.94 | 2.23 | 1.59 | 1.52 | 2.04 |
| 191.63 | 257.16 | 230.8 | 163.26 | 148.51 | 217.2 |
| 1.55 | 1.94 | 2.23 | 1.59 | 1.52 | 2.04 |
| 315.64 | 354.11 | 329.03 | 637.85 | 449.98 | 431.16 |
| 78.47 | 76.82 | 101.3 | 58.5 | 64.77 | 80.67 |
| 168.38 | 146.63 | 154.89 | 160.09 | 166.79 | 151.99 |
| 1.55 | 1.94 | 2.23 | 1.59 | 1.52 | 2.04 |
| 528.02 | 577.1 | 478.62 | 480.71 | 561.13 | 667.54 |
| 941.91 | 912.56 | 960.86 | 952.13 | 1124.47 | 1077.12 |
| 10008.81 | 9314.5 | 9335.38 | 9297.9 | 9626.42 | 9863.79 |
| 135.82 | 84.58 | 94.61 | 85.49 | 116.54 | 127.54 |
| 893.85 | 889.29 | 918.44 | 947.36 | 896.09 | 954.86 |
| 438.11 | 516.99 | 494.24 | 331.51 | 555.04 | 429.13 |
| 1.55 | 1.94 | 2.23 | 1.59 | 1.52 | 2.04 |
| 121.87 | 111.73 | 67.81 | 83.9 | 119.59 | 96.98 |
| 1.55 | 1.94 | 2.23 | 1.59 | 1.52 | 2.04 |
| 1.55 | 1.94 | 2.23 | 1.59 | 1.52 | 2.04 |
| 6198.51 | 7133.06 | 6531.22 | 7264.63 | 7385.22 | 8633.01 |
| 4966.13 | 5131.95 | 5088.95 | 5045.65 | 5276.48 | 5177.02 |

| 266.04 | 340.53 | 201.77 | 234.69 | 258.14 | 388.37 |
| --- | --- | --- | --- | --- | --- |
| 103.27 | 134.99 | 108 | 153.74 | 146.99 | 151.99 |
| 4.06 | 1.94 | 2.23 | 1.59 | 1.52 | 2.04 |
| 97.07 | 61.31 | 76.75 | 22 | 61.73 | 101.05 |
| 33.51 | 55.49 | 49.95 | 18.82 | 8.44 | 17.5 |
| 233.48 | 381.25 | 279.91 | 206.12 | 468.25 | 280.37 |
| 216.43 | 295.94 | 268.75 | 134.69 | 201.8 | 221.28 |
| 6534.9 | 6933.34 | 6595.96 | 7034.48 | 7298.44 | 8044.1 |
| 86.22 | 69.07 | 56.65 | 44.22 | 75.43 | 109.2 |
| 8.71 | 14.77 | 9.77 | 9.3 | 16.05 | 15.47 |
| 309.44 | 373.5 | 304.47 | 301.35 | 367.76 | 339.47 |
| 214.88 | 261.03 | 291.08 | 271.2 | 249 | 294.64 |
| 334.25 | 330.84 | 380.38 | 331.51 | 422.57 | 382.26 |
| 340.45 | 404.52 | 387.08 | 350.56 | 456.07 | 372.07 |
| 166.83 | 233.89 | 210.7 | 53.74 | 258.14 | 129.58 |
| 946.56 | 1021.14 | 1099.28 | 496.59 | 1330.02 | 993.57 |
| 236.58 | 338.6 | 324.56 | 136.28 | 418.01 | 231.47 |
| 13.36 | 24.47 | 2.23 | 14.06 | 26.71 | 39.92 |
| 7.16 | 1.94 | 2.23 | 2.95 | 1.52 | 3.24 |
| 244.34 | 313.39 | 311.17 | 183.9 | 285.54 | 294.64 |
| 97.07 | 119.48 | 152.65 | 110.88 | 157.65 | 135.69 |
| 1.55 | 1.94 | 2.23 | 1.59 | 1.52 | 2.04 |
| 1.55 | 1.94 | 2.23 | 1.59 | 1.52 | 2.04 |
| 1.55 | 5.08 | 12 | 1.59 | 1.52 | 2.04 |
| 1763.5 | 1882.09 | 1695.39 | 2117.17 | 2108.04 | 2181.57 |
| 255.19 | 307.57 | 244.19 | 137.87 | 213.98 | 288.52 |
| 2.51 | 1.94 | 2.23 | 1.59 | 1.52 | 13.43 |
| 1.55 | 1.94 | 2.23 | 4.54 | 1.52 | 2.04 |
| 1.55 | 1.94 | 2.23 | 1.59 | 1.52 | 2.04 |
| 111.02 | 105.91 | 101.3 | 106.12 | 108.93 | 86.79 |
| 532.67 | 474.33 | 465.22 | 575.95 | 421.05 | 394.48 |
| 154.43 | 117.54 | 157.12 | 109.3 | 140.9 | 141.81 |
| 456.71 | 326.96 | 485.31 | 775.94 | 404.3 | 632.9 |
| 2016.17 | 2188.46 | 1923.11 | 2396.53 | 2816.03 | 2733.79 |
| 932.61 | 867.96 | 902.81 | 937.84 | 925.02 | 938.56 |
| 1.55 | 1.94 | 2.23 | 1.59 | 1.52 | 2.04 |
| 1.55 | 1.94 | 2.23 | 1.59 | 1.52 | 2.04 |
| 1.55 | 1.94 | 2.23 | 1.59 | 1.52 | 2.04 |
| 14.91 | 1.94 | 9.77 | 29.93 | 3.87 | 21.58 |
| 217.98 | 326.96 | 302.24 | 91.84 | 393.65 | 347.62 |
| 424.15 | 551.89 | 391.54 | 266.43 | 520.02 | 370.03 |
| 163.73 | 140.81 | 132.56 | 172.79 | 194.19 | 141.81 |
| 27.31 | 1.94 | 16.46 | 18.82 | 13.01 | 23.62 |
| 4.06 | 1.94 | 2.23 | 4.54 | 17.58 | 31.77 |
| 2.51 | 1.94 | 2.23 | 1.59 | 1.52 | 7.32 |
| 1.55 | 1.94 | 2.23 | 1.59 | 1.52 | 2.04 |
| 205.58 | 216.43 | 248.66 | 215.64 | 213.98 | 229.43 |
| 827.2 | 2128.35 | 1655.2 | 1852.1 | 1386.35 | 1549.87 |

| 159.08 | 216.43 | 241.96 | 152.15 | 175.92 | 202.94 |
| --- | --- | --- | --- | --- | --- |
| 1.55 | 1.94 | 2.23 | 1.59 | 1.52 | 2.04 |
| 3366.36 | 3225.86 | 3039.42 | 3363.16 | 3140.34 | 3412.35 |
| 3569.44 | 3295.66 | 3418.96 | 3372.69 | 3555.99 | 3781.18 |
| 115.67 | 119.48 | 76.75 | 48.98 | 83.05 | 82.71 |
| 4062.39 | 4034.44 | 3865.48 | 4056.79 | 4250.28 | 4298.77 |
| 3907.37 | 3580.7 | 3861.02 | 3877.43 | 3890.96 | 4115.37 |
| 18.01 | 14.77 | 36.56 | 7.71 | 17.58 | 19.54 |
| 1.55 | 1.94 | 2.23 | 1.59 | 1.52 | 2.04 |
| 506.31 | 548.01 | 476.38 | 299.77 | 527.63 | 323.16 |
| 1796.05 | 1725.02 | 1744.5 | 1680.68 | 2202.44 | 1875.91 |
| 631.88 | 648.85 | 610.34 | 536.27 | 591.58 | 545.28 |
| 278.44 | 264.91 | 268.75 | 198.18 | 284.02 | 176.45 |
| 55.22 | 65.19 | 90.14 | 77.55 | 81.52 | 109.2 |
| 56.77 | 59.37 | 58.88 | 79.14 | 89.14 | 119.39 |
| 1282.94 | 1203.42 | 1213.14 | 1182.28 | 1264.55 | 1344.06 |
| 2.51 | 1.94 | 2.23 | 1.59 | 1.52 | 2.04 |
| 1.55 | 1.94 | 2.23 | 1.59 | 1.52 | 2.04 |
| 366.8 | 454.94 | 398.24 | 336.27 | 465.21 | 410.79 |
| 250.54 | 259.09 | 273.21 | 148.98 | 422.57 | 211.09 |
| 138.92 | 131.12 | 141.49 | 145.8 | 182.01 | 141.81 |
| 148.23 | 117.54 | 137.03 | 110.88 | 306.86 | 166.26 |
| 1.55 | 1.94 | 2.23 | 1.59 | 1.52 | 2.04 |
| 373 | 295.94 | 967.56 | 588.65 | 827.57 | 1922.78 |
| 276.89 | 158.26 | 715.27 | 429.92 | 678.36 | 1482.63 |
| 13.36 | 14.77 | 20.93 | 23.58 | 38.89 | 13.43 |
| 11.81 | 20.59 | 49.95 | 1.59 | 19.1 | 50.11 |
| 1.55 | 1.94 | 2.23 | 1.59 | 1.52 | 2.04 |
| 112.57 | 233.89 | 141.49 | 99.77 | 110.45 | 149.96 |
| 1.55 | 1.94 | 2.23 | 1.59 | 1.52 | 2.04 |
| 14.91 | 1.94 | 12 | 29.93 | 3.87 | 15.47 |
| 1165.13 | 1139.43 | 1117.14 | 1068 | 1331.54 | 1156.59 |
| 1.55 | 1.94 | 2.23 | 1.59 | 1.52 | 2.04 |
| 1.55 | 5.08 | 2.23 | 6.12 | 16.05 | 2.04 |
| 1549.57 | 1350.78 | 1378.36 | 1290.21 | 1741.11 | 1562.1 |
| 1019.42 | 1277.1 | 1309.15 | 1198.15 | 1127.52 | 1123.99 |
| 62.97 | 111.73 | 81.21 | 12.47 | 105.88 | 64.37 |
| 138.92 | 166.02 | 112.47 | 61.68 | 440.85 | 245.73 |
| 1.55 | 1.94 | 2.23 | 1.59 | 1.52 | 5.28 |
| 1295.35 | 1346.91 | 1306.91 | 1333.07 | 1412.24 | 1317.57 |
| 642.73 | 1193.72 | 842.53 | 474.36 | 684.45 | 626.78 |
| 552.82 | 714.77 | 632.67 | 371.19 | 465.21 | 408.75 |
| 2509.12 | 3053.28 | 3072.91 | 1894.95 | 2479.55 | 2548.36 |
| 2.51 | 1.94 | 2.23 | 1.59 | 1.52 | 2.04 |
| 72.27 | 94.27 | 65.58 | 36.28 | 48.03 | 56.22 |
| 664.43 | 728.35 | 864.86 | 826.73 | 702.73 | 781.65 |
| 162.18 | 164.08 | 233.03 | 248.97 | 220.08 | 268.14 |
| 49.02 | 47.74 | 38.79 | 48.98 | 54.12 | 56.22 |

| 22.66 | 24.47 | 27.63 | 17.24 | 48.03 | 41.96 |
| --- | --- | --- | --- | --- | --- |
| 112.57 | 94.27 | 101.3 | 126.76 | 76.95 | 141.81 |
| 357.5 | 646.91 | 378.15 | 88.66 | 440.85 | 178.48 |
| 303.24 | 315.33 | 215.17 | 168.02 | 317.52 | 270.18 |
| 755.89 | 780.7 | 809.04 | 788.64 | 914.36 | 818.33 |
| 1.55 | 1.94 | 2.23 | 1.59 | 1.52 | 2.04 |
| 6663.56 | 5905.64 | 6662.94 | 7513.83 | 8347.48 | 6731.81 |
| 44.36 | 65.19 | 29.86 | 63.27 | 29.76 | 62.33 |
| 3774.06 | 3541.92 | 3012.63 | 2555.25 | 3082.48 | 3031.3 |
| 104.82 | 133.06 | 130.33 | 152.15 | 288.59 | 170.33 |
| 146.68 | 119.48 | 132.56 | 129.93 | 128.72 | 125.5 |
| 413.3 | 435.55 | 592.48 | 372.78 | 418.01 | 500.45 |
| 104.82 | 67.13 | 103.54 | 95.01 | 122.63 | 151.99 |
| 405.55 | 423.91 | 346.89 | 593.41 | 523.06 | 655.31 |
| 467.56 | 511.17 | 543.36 | 561.66 | 533.72 | 604.37 |
| 8210.62 | 8238.33 | 7542.59 | 7967.78 | 8021.65 | 8302.89 |
| 1.55 | 1.94 | 2.23 | 1.59 | 1.52 | 2.04 |
| 81.57 | 57.43 | 76.75 | 37.87 | 78.48 | 70.49 |
| 35.06 | 24.47 | 25.4 | 15.65 | 20.62 | 29.73 |
| 89.32 | 162.14 | 114.7 | 18.82 | 159.17 | 54.18 |
| 165.28 | 208.68 | 204 | 110.88 | 203.33 | 117.35 |
| 49.02 | 69.07 | 49.95 | 61.68 | 40.41 | 84.75 |
| 1.55 | 1.94 | 2.23 | 1.59 | 1.52 | 2.04 |
| 1.55 | 1.94 | 2.23 | 1.59 | 8.44 | 5.28 |
| 22.66 | 14.77 | 25.4 | 4.54 | 8.44 | 31.77 |
| 22.66 | 32.22 | 43.26 | 25.17 | 28.23 | 41.96 |
| 5.61 | 1.94 | 2.23 | 1.59 | 1.52 | 2.04 |
| 114.12 | 146.63 | 105.77 | 52.15 | 198.76 | 82.71 |
| 428.81 | 499.54 | 389.31 | 745.78 | 731.65 | 767.39 |
| 145.13 | 142.75 | 116.93 | 98.18 | 156.13 | 164.22 |
| 1.55 | 1.94 | 2.23 | 1.59 | 1.52 | 2.04 |
| 8.71 | 1.94 | 2.23 | 1.59 | 1.52 | 2.04 |
| 28.86 | 53.55 | 63.35 | 31.52 | 26.71 | 41.96 |
| 1.55 | 1.94 | 2.23 | 1.59 | 1.52 | 2.04 |
| 47.47 | 36.1 | 43.26 | 34.69 | 26.71 | 48.07 |
| 18.01 | 36.1 | 9.77 | 7.71 | 57.16 | 11.39 |
| 13.36 | 28.35 | 25.4 | 18.82 | 14.53 | 25.66 |
| 1.55 | 1.94 | 2.23 | 1.59 | 1.52 | 2.04 |
| 155.98 | 274.61 | 130.33 | 45.81 | 405.83 | 109.2 |
| 10.26 | 8.96 | 5.3 | 1.59 | 2.35 | 11.39 |
| 154.43 | 76.82 | 157.12 | 161.68 | 125.68 | 164.22 |
| 1.55 | 3.14 | 9.77 | 4.54 | 13.01 | 2.04 |
| 1.55 | 1.94 | 2.23 | 1.59 | 1.52 | 2.04 |
| 1.55 | 1.94 | 2.23 | 1.59 | 1.52 | 2.04 |
| 1.55 | 1.94 | 2.23 | 1.59 | 1.52 | 2.04 |
| 1.55 | 1.94 | 2.23 | 1.59 | 1.52 | 2.04 |
| 3938.37 | 3923.92 | 4044.09 | 3647.28 | 3673.23 | 3785.26 |
| 10.26 | 1.94 | 3.07 | 1.59 | 2.35 | 2.04 |

| 81.57 | 84.58 | 85.68 | 93.42 | 54.12 | 76.6 |
| --- | --- | --- | --- | --- | --- |
| 404 | 338.6 | 438.43 | 410.87 | 431.71 | 437.28 |
| 39.71 | 30.29 | 27.63 | 77.55 | 57.16 | 29.73 |
| 47.47 | 38.04 | 36.56 | 52.15 | 43.46 | 48.07 |
| 354.4 | 390.95 | 366.98 | 369.61 | 488.04 | 418.94 |
| 7.16 | 1.94 | 12 | 1.59 | 9.96 | 29.73 |
| 50.57 | 53.55 | 34.33 | 128.34 | 105.88 | 99.01 |
| 146.68 | 103.97 | 145.96 | 104.53 | 130.24 | 145.88 |
| 11.81 | 1.94 | 2.23 | 1.59 | 5.39 | 2.04 |
| 44.36 | 51.61 | 61.12 | 61.68 | 51.07 | 52.15 |
| 895.4 | 900.92 | 907.28 | 1277.51 | 1097.07 | 1490.78 |
| 2630.04 | 2737.21 | 2258 | 2631.44 | 2633.33 | 2786.77 |
| 579.17 | 720.59 | 563.45 | 418.81 | 1186.9 | 537.12 |
| 439.66 | 437.49 | 407.17 | 571.19 | 494.14 | 581.95 |
| 2268.85 | 2471.56 | 3101.93 | 2937.78 | 2848.01 | 3241.19 |
| 1.55 | 1.94 | 2.23 | 1.59 | 1.52 | 2.04 |
| 1.55 | 1.94 | 2.23 | 1.59 | 1.52 | 2.04 |
| 28.86 | 22.53 | 27.63 | 29.93 | 9.96 | 31.77 |
| 188.53 | 212.56 | 241.96 | 187.07 | 168.31 | 292.6 |
| 1734.04 | 1905.36 | 1715.48 | 1763.21 | 2048.66 | 2014.48 |
| 583.82 | 538.32 | 612.57 | 501.35 | 509.36 | 522.86 |
| 3722.9 | 3664.08 | 3769.48 | 4052.03 | 3878.78 | 4294.69 |
| 771.39 | 883.47 | 876.02 | 844.19 | 885.43 | 775.54 |
| 75241.23 | 75481.05 | 76371.7 | 74753.02 | 77422.79 | 80493.38 |
| 177.68 | 158.26 | 172.75 | 234.69 | 166.79 | 194.79 |
| 1484.47 | 1395.38 | 1523.48 | 1418.78 | 1531 | 1437.8 |
| 10428.91 | 10615.61 | 10750.85 | 11194.67 | 11349.96 | 11377.82 |
| 186.98 | 222.25 | 154.89 | 174.37 | 172.88 | 125.5 |
| 1912.31 | 2050.79 | 2048.14 | 1728.29 | 2025.83 | 2053.19 |
| 18080.51 | 17689.3 | 17895.2 | 18797.61 | 18711.51 | 18768.65 |
| 12690.59 | 12880.44 | 11764.45 | 13653.32 | 14034.22 | 14491.47 |
| 4609.6 | 4941.92 | 4655.83 | 4685.34 | 4873.01 | 5101.63 |
| 394.7 | 334.72 | 440.66 | 404.52 | 341.88 | 345.58 |
| 617.93 | 604.25 | 534.43 | 456.9 | 532.2 | 579.92 |

| 1Aged Repop 6h | 2Aged Repop 6h | 3Aged Repop 6h | 1Young Con 48h | 2Young Con 48h |
| --- | --- | --- | --- | --- |
| 336.07 | 332.13 | 310.48 | 316.56 | 307.43 |
| 247.72 | 272.94 | 290.5 | 1084 | 974 |
| 591.33 | 650.7 | 544.27 | 467.8 | 478.09 |
| 169.18 | 159.78 | 136.64 | 174.51 | 166.89 |
| 205.18 | 229.42 | 186.6 | 425.9 | 449.98 |
| 98.82 | 142.38 | 80.69 | 138.75 | 125.73 |
| 1.64 | 1.74 | 2 | 1.02 | 1 |
| 12.1 | 1.74 | 8.76 | 1.02 | 1 |
| 29585.13 | 31512.36 | 30730.64 | 35880.64 | 35660.57 |
| 273.9 | 319.94 | 410.39 | 369.7 | 296.39 |
| 1645.05 | 1608.17 | 1517.38 | 1626.63 | 1522.11 |
| 134.82 | 130.19 | 112.66 | 138.75 | 124.73 |
| 7947.77 | 10112.18 | 11628.12 | 6897.58 | 5408.07 |
| 10.47 | 10.07 | 16.75 | 1.02 | 1 |
| 21.92 | 18.78 | 30.74 | 12.03 | 2.26 |
| 375.34 | 386.09 | 400.4 | 498.45 | 401.8 |
| 1929.75 | 2119.97 | 2248.71 | 1615.39 | 1791.15 |
| 164.27 | 182.42 | 174.61 | 174.51 | 151.83 |
| 15.38 | 37.93 | 78.7 | 1.02 | 1 |
| 556.96 | 685.52 | 730.1 | 507.65 | 461.02 |
| 270.63 | 246.83 | 250.54 | 177.58 | 175.93 |
| 257.54 | 222.45 | 234.55 | 146.92 | 105.65 |
| 2.29 | 1.74 | 42.73 | 1.02 | 1 |
| 262.44 | 415.69 | 536.28 | 183.71 | 103.65 |
| 21.92 | 11.81 | 16.75 | 13.05 | 15.31 |
| 30.1 | 34.44 | 22.75 | 12.03 | 17.31 |
| 1.64 | 1.74 | 2 | 1.02 | 1 |
| 1.64 | 1.74 | 2 | 1.02 | 1 |
| 1555.06 | 1743.95 | 2100.84 | 944 | 806.35 |
| 2523.7 | 2680.52 | 3371.68 | 1592.91 | 1435.78 |
| 2075.38 | 1968.52 | 1996.94 | 1900.5 | 1814.23 |
| 89.01 | 149.34 | 170.61 | 51.89 | 28.36 |
| 30.1 | 67.52 | 64.71 | 27.36 | 27.35 |
| 74.28 | 34.44 | 70.7 | 51.89 | 31.37 |
| 35.01 | 37.93 | 116.66 | 5.9 | 18.32 |
| 1332.53 | 1517.64 | 2456.52 | 396.27 | 257.24 |
| 1.64 | 1.74 | 2 | 1.02 | 1 |
| 1.64 | 1.74 | 2 | 1.02 | 1 |
| 1.64 | 1.74 | 2 | 11.01 | 1 |
| 1.64 | 1.74 | 2 | 1.02 | 1 |
| 30.1 | 4.85 | 26.74 | 6.92 | 1 |
| 1.64 | 3.11 | 2 | 1.02 | 1 |
| 1.64 | 1.74 | 2 | 1.02 | 1 |
| 2.29 | 1.74 | 44.73 | 1.02 | 1 |
| 39.92 | 67.52 | 64.71 | 26.34 | 27.35 |
| 89.01 | 77.97 | 94.68 | 68.24 | 60.48 |
| 129.91 | 147.6 | 156.62 | 197 | 228.13 |

| 241.17 | 236.38 | 206.58 | 294.08 | 301.41 |
| --- | --- | --- | --- | --- |
| 49.74 | 105.82 | 122.66 | 70.28 | 47.43 |
| 26.83 | 8.33 | 18.75 | 8.97 | 9.28 |
| 12.1 | 71 | 170.61 | 1.02 | 1 |
| 149.55 | 520.14 | 92.68 | 18.16 | 5.27 |
| 107 | 114.52 | 202.58 | 41.67 | 22.33 |
| 115.19 | 286.87 | 1259.61 | 1.02 | 3.26 |
| 1.64 | 1.74 | 2 | 1.02 | 1 |
| 1.64 | 20.52 | 2.76 | 5.9 | 1 |
| 1.64 | 1.74 | 2 | 1.02 | 1 |
| 218.27 | 276.42 | 246.54 | 157.14 | 149.82 |
| 1.64 | 1.74 | 2 | 1.02 | 1 |
| 21.92 | 36.18 | 60.71 | 1.02 | 4.26 |
| 1.64 | 3.11 | 2 | 1.02 | 1 |
| 30.1 | 137.15 | 818.02 | 18.16 | 10.29 |
| 35.01 | 3.11 | 30.74 | 7.94 | 16.31 |
| 15.38 | 24 | 130.65 | 176.56 | 165.89 |
| 1.64 | 3.11 | 18.75 | 4.88 | 1 |
| 193.72 | 325.16 | 518.29 | 39.62 | 38.4 |
| 1.64 | 1.74 | 2 | 1.02 | 1 |
| 3.92 | 4.85 | 2 | 1.02 | 1 |
| 1.64 | 1.74 | 2 | 1.02 | 1 |
| 1.64 | 1.74 | 2 | 1.02 | 1 |
| 66.1 | 102.34 | 32.74 | 63.13 | 25.35 |
| 1.64 | 1.74 | 2 | 1.02 | 1 |
| 1.64 | 1.74 | 4.76 | 1.02 | 1 |
| 1.64 | 1.74 | 2 | 1.02 | 1 |
| 1.64 | 6.59 | 2 | 1.02 | 1 |
| 1.64 | 1.74 | 2 | 11.01 | 3.26 |
| 35.01 | 84.93 | 90.68 | 14.08 | 9.28 |
| 72.64 | 93.63 | 138.64 | 35.54 | 23.34 |
| 494.79 | 495.77 | 1309.57 | 34.51 | 44.42 |
| 1.64 | 1.74 | 2 | 1.02 | 1 |
| 5.56 | 4.85 | 2 | 19.18 | 12.3 |
| 1949.39 | 2212.24 | 2056.88 | 1799.33 | 1792.15 |
| 1.64 | 1.74 | 2 | 1.02 | 1 |
| 44.83 | 46.63 | 36.73 | 32.47 | 37.39 |
| 1.64 | 1.74 | 2 | 1.02 | 1 |
| 1.64 | 1.74 | 2 | 1.02 | 1 |
| 1.64 | 1.74 | 2 | 1.02 | 1 |
| 1.64 | 1.74 | 2 | 1.02 | 1 |
| 43.19 | 91.89 | 68.7 | 1.02 | 1 |
| 1.64 | 1.74 | 4.76 | 1.02 | 1.25 |
| 234.63 | 283.38 | 270.52 | 200.06 | 202.03 |
| 1.64 | 1.74 | 2 | 1.02 | 1 |
| 182.27 | 429.62 | 436.37 | 42.69 | 35.38 |
| 1.64 | 1.74 | 2 | 3.86 | 2.26 |
| 1738.31 | 1571.61 | 1875.05 | 2222.4 | 1747.98 |

| 17.01 | 1.74 | 2 | 13.05 | 18.32 |
| --- | --- | --- | --- | --- |
| 1.64 | 1.74 | 6.76 | 7.94 | 1.25 |
| 1.64 | 1.74 | 2 | 1.02 | 1 |
| 125 | 112.78 | 134.64 | 85.61 | 89.59 |
| 93.91 | 121.49 | 50.72 | 86.63 | 112.68 |
| 2.29 | 53.59 | 12.76 | 5.9 | 8.28 |
| 1.64 | 1.74 | 2 | 1.02 | 1 |
| 265.72 | 394.8 | 650.17 | 142.83 | 153.84 |
| 1.64 | 1.74 | 2 | 1.02 | 1 |
| 8.83 | 17.04 | 8.76 | 13.05 | 11.29 |
| 7.19 | 1.74 | 2 | 1.81 | 1 |
| 5.56 | 1.74 | 2 | 2.83 | 1.25 |
| 77.55 | 86.67 | 58.71 | 102.98 | 103.65 |
| 1.64 | 18.78 | 2 | 1.81 | 9.28 |
| 1.64 | 1.74 | 2.76 | 22.25 | 10.29 |
| 1.64 | 1.74 | 2 | 1.02 | 1 |
| 20.28 | 24 | 4.76 | 5.9 | 7.28 |
| 484.97 | 629.81 | 1131.73 | 205.17 | 120.71 |
| 1.64 | 1.74 | 2 | 1.02 | 1 |
| 1.64 | 1.74 | 2 | 4.88 | 1 |
| 5.56 | 17.04 | 32.74 | 1.02 | 7.28 |
| 11841.97 | 13050.72 | 13952 | 13550.13 | 14780.17 |
| 439.16 | 528.84 | 608.21 | 301.23 | 366.66 |
| 810.58 | 816.08 | 877.96 | 671.16 | 857.55 |
| 156.09 | 126.71 | 136.64 | 36.56 | 20.33 |
| 1.64 | 1.74 | 2 | 1.02 | 1 |
| 1.64 | 1.74 | 2 | 1.02 | 1 |
| 1465.07 | 1677.8 | 1777.14 | 1512.18 | 1389.6 |
| 1.64 | 1.74 | 2 | 1.02 | 1 |
| 66.1 | 79.71 | 118.66 | 178.6 | 133.76 |
| 208.45 | 265.98 | 252.54 | 152.03 | 148.82 |
| 372.07 | 534.07 | 562.25 | 141.81 | 119.71 |
| 2281.54 | 2109.53 | 2940.08 | 403.42 | 441.95 |
| 26.83 | 29.22 | 20.75 | 13.05 | 12.3 |
| 676.41 | 753.41 | 847.99 | 172.47 | 140.79 |
| 1.64 | 24 | 56.72 | 1.02 | 1 |
| 1.64 | 1.74 | 2 | 1.02 | 1 |
| 545.51 | 567.14 | 694.13 | 489.26 | 368.67 |
| 1.64 | 1.74 | 2 | 1.02 | 1 |
| 113.55 | 100.6 | 124.65 | 238.89 | 195 |
| 961.11 | 936.2 | 977.87 | 978.75 | 853.53 |
| 1.64 | 1.74 | 2 | 1.02 | 1 |
| 25.19 | 11.81 | 24.74 | 36.56 | 26.35 |
| 1.64 | 1.74 | 2 | 1.02 | 1 |
| 12.1 | 25.74 | 40.73 | 1.02 | 1 |
| 56.28 | 51.85 | 100.68 | 31.45 | 12.3 |
| 25285.15 | 26312.46 | 31256.16 | 25367.36 | 23456.58 |
| 46.46 | 51.85 | 66.71 | 68.24 | 27.35 |

| 1.64 | 1.74 | 2 | 1.02 | 1 |
| --- | --- | --- | --- | --- |
| 228.08 | 274.68 | 224.56 | 145.9 | 178.94 |
| 970.93 | 971.02 | 1023.83 | 679.33 | 610.6 |
| 583.14 | 734.26 | 1139.72 | 464.73 | 441.95 |
| 650.23 | 671.59 | 544.27 | 1202.54 | 964.96 |
| 1.64 | 1.74 | 2 | 1.02 | 1 |
| 61.19 | 46.63 | 104.67 | 1.02 | 1 |
| 39.92 | 24 | 46.72 | 47.8 | 33.38 |
| 1.64 | 1.74 | 2 | 1.02 | 1 |
| 9045.67 | 8808.29 | 8834.68 | 8402.83 | 8295.19 |
| 103.73 | 128.45 | 222.56 | 262.4 | 183.96 |
| 1.64 | 1.74 | 2 | 1.02 | 1 |
| 3680.51 | 3869.52 | 4722.44 | 2543.27 | 2313.16 |
| 445.7 | 492.29 | 520.29 | 403.42 | 377.7 |
| 4227 | 4391.77 | 3979.12 | 2923.42 | 2568.14 |
| 262.44 | 292.09 | 296.5 | 577.14 | 534.31 |
| 71.01 | 65.78 | 254.53 | 1.02 | 1 |
| 67.73 | 816.08 | 1205.66 | 29.4 | 1 |
| 1.64 | 22.26 | 2 | 1.02 | 1 |
| 736.95 | 701.19 | 706.12 | 1465.17 | 1119.56 |
| 1.64 | 30.96 | 2 | 1.02 | 1 |
| 1.64 | 1.74 | 2 | 1.02 | 1 |
| 3.92 | 1.74 | 8.76 | 1.02 | 1 |
| 3.92 | 81.45 | 12.76 | 8.97 | 12.3 |
| 1.64 | 1.74 | 2 | 1.02 | 1 |
| 8.83 | 1.74 | 2 | 1.02 | 2.26 |
| 1.64 | 1.74 | 2 | 1.02 | 1 |
| 30.1 | 6.59 | 32.74 | 38.6 | 60.48 |
| 1.64 | 1.74 | 2 | 1.02 | 1 |
| 1.64 | 1.74 | 2 | 1.02 | 1 |
| 64.46 | 116.26 | 212.57 | 18.16 | 22.33 |
| 303.35 | 535.81 | 670.15 | 198.02 | 146.81 |
| 74.28 | 48.37 | 82.69 | 14.08 | 26.35 |
| 1.64 | 1.74 | 2 | 1.02 | 1 |
| 10.47 | 13.55 | 14.75 | 27.36 | 6.27 |
| 62.83 | 83.19 | 98.68 | 53.93 | 34.38 |
| 141.36 | 131.93 | 162.62 | 205.17 | 169.9 |
| 228.08 | 203.31 | 198.59 | 208.24 | 177.93 |
| 28.47 | 18.78 | 14.75 | 135.68 | 128.74 |
| 229.72 | 208.53 | 294.5 | 360.5 | 300.4 |
| 26.83 | 30.96 | 36.73 | 35.54 | 14.3 |
| 152.82 | 145.86 | 206.58 | 11.01 | 13.3 |
| 1.64 | 1.74 | 2 | 1.02 | 1 |
| 1.64 | 1.74 | 2 | 1.02 | 1 |
| 1.64 | 1.74 | 2 | 1.02 | 1 |
| 144.64 | 151.08 | 228.56 | 93.78 | 78.55 |
| 141.36 | 229.42 | 246.54 | 107.07 | 71.52 |
| 812.21 | 977.98 | 1771.15 | 257.29 | 184.96 |

| 452.25 | 532.32 | 520.29 | 184.73 | 121.72 |
| --- | --- | --- | --- | --- |
| 84.1 | 97.11 | 230.56 | 18.16 | 6.27 |
| 224.81 | 201.56 | 240.55 | 327.8 | 268.28 |
| 686.23 | 636.78 | 726.1 | 235.83 | 191.99 |
| 682.95 | 810.86 | 899.94 | 671.16 | 495.15 |
| 1.64 | 1.74 | 2 | 1.02 | 1 |
| 1.64 | 1.74 | 2 | 1.02 | 1 |
| 664.95 | 687.26 | 546.27 | 809.11 | 773.23 |
| 3642.87 | 3563.13 | 3387.67 | 3861.52 | 3825.98 |
| 30.1 | 39.67 | 42.73 | 14.08 | 42.41 |
| 1.64 | 1.74 | 2 | 1.02 | 1 |
| 13.74 | 24 | 28.74 | 13.05 | 19.32 |
| 71.01 | 84.93 | 100.68 | 122.4 | 93.61 |
| 213.36 | 236.38 | 236.55 | 185.75 | 167.89 |
| 3.92 | 13.55 | 8.76 | 1.02 | 9.28 |
| 1.64 | 1.74 | 2 | 1.02 | 1 |
| 1.64 | 1.74 | 2 | 1.02 | 1 |
| 1.64 | 1.74 | 2 | 1.02 | 1 |
| 201.9 | 274.68 | 396.4 | 115.24 | 63.49 |
| 133.18 | 131.93 | 218.57 | 45.75 | 13.3 |
| 280.44 | 267.72 | 268.52 | 266.48 | 202.03 |
| 13.74 | 3.11 | 2 | 1.81 | 1 |
| 1.64 | 1.74 | 2 | 1.02 | 1 |
| 33.37 | 76.22 | 94.68 | 36.56 | 11.29 |
| 406.43 | 612.4 | 784.05 | 428.97 | 301.41 |
| 13.74 | 1.74 | 2.76 | 1.81 | 1 |
| 1.64 | 1.74 | 2 | 1.02 | 1 |
| 1.64 | 1.74 | 2 | 1.02 | 1 |
| 1.64 | 1.74 | 2 | 1.02 | 1 |
| 1.64 | 1.74 | 2 | 1.02 | 1 |
| 1.64 | 1.74 | 2 | 1.02 | 1 |
| 304.99 | 271.2 | 270.52 | 191.89 | 168.9 |
| 1275.26 | 1383.6 | 1359.52 | 1033.93 | 1102.49 |
| 7.19 | 1.74 | 2 | 1.02 | 1 |
| 121.73 | 231.16 | 270.52 | 31.45 | 14.3 |
| 39.92 | 32.7 | 46.72 | 146.92 | 112.68 |
| 17.01 | 24 | 30.74 | 5.9 | 7.28 |
| 907.12 | 830.01 | 861.98 | 795.83 | 565.43 |
| 1.64 | 1.74 | 2 | 1.02 | 1 |
| 236.27 | 191.12 | 260.53 | 154.08 | 137.78 |
| 30.1 | 90.15 | 126.65 | 9.99 | 1 |
| 241.17 | 389.58 | 470.34 | 96.85 | 53.45 |
| 340.98 | 567.14 | 654.17 | 204.15 | 156.85 |
| 509.51 | 1249.55 | 971.88 | 310.43 | 295.39 |
| 246.08 | 413.95 | 450.36 | 126.48 | 109.67 |
| 1.64 | 1.74 | 2 | 1.02 | 1 |
| 1.64 | 1.74 | 2 | 1.02 | 1 |
| 1170.55 | 1260 | 1287.59 | 1652.18 | 1622.5 |

| 347.53 | 434.84 | 560.25 | 420.79 | 355.62 |
| --- | --- | --- | --- | --- |
| 1.64 | 1.74 | 2 | 1.02 | 1 |
| 1.64 | 1.74 | 2 | 1.02 | 1 |
| 619.14 | 680.3 | 746.08 | 469.84 | 433.92 |
| 741.86 | 857.86 | 784.05 | 956.27 | 834.46 |
| 354.07 | 462.69 | 444.36 | 366.63 | 324.5 |
| 594.6 | 685.52 | 592.23 | 557.72 | 547.36 |
| 285.35 | 377.39 | 294.5 | 368.67 | 375.69 |
| 28.47 | 37.93 | 100.68 | 21.23 | 11.29 |
| 380.25 | 333.87 | 358.44 | 362.54 | 339.56 |
| 12.1 | 4.85 | 2.76 | 14.08 | 5.27 |
| 159.36 | 151.08 | 134.64 | 132.62 | 125.73 |
| 1.64 | 1.74 | 2 | 1.02 | 1 |
| 228.08 | 239.86 | 276.51 | 242.98 | 197.01 |
| 1.64 | 1.74 | 2 | 1.02 | 1 |
| 80.82 | 69.26 | 72.7 | 101.96 | 113.69 |
| 190.45 | 253.79 | 308.48 | 341.08 | 248.2 |
| 12.1 | 1.74 | 10.76 | 7.94 | 13.3 |
| 8.83 | 13.55 | 8.76 | 34.51 | 12.3 |
| 1.64 | 1.74 | 2 | 1.02 | 1 |
| 1.64 | 1.74 | 2 | 1.02 | 1 |
| 1.64 | 1.74 | 2 | 1.02 | 1 |
| 1.64 | 1.74 | 2 | 1.02 | 1 |
| 301.71 | 433.1 | 602.22 | 157.14 | 177.93 |
| 1.64 | 8.33 | 2 | 1.81 | 8.28 |
| 33.37 | 34.44 | 34.74 | 19.18 | 22.33 |
| 232.99 | 302.53 | 292.5 | 307.36 | 325.5 |
| 1.64 | 1.74 | 2 | 1.02 | 1 |
| 1.64 | 1.74 | 2 | 1.02 | 1 |
| 1.64 | 1.74 | 4.76 | 1.02 | 1 |
| 394.98 | 520.14 | 606.21 | 281.81 | 213.07 |
| 25.19 | 25.74 | 14.75 | 22.25 | 31.37 |
| 1.64 | 1.74 | 2 | 1.02 | 1.25 |
| 1092.01 | 1061.54 | 851.99 | 1139.18 | 1212.92 |
| 1.64 | 1.74 | 2 | 1.02 | 1 |
| 2.29 | 1.74 | 2 | 1.02 | 1 |
| 1.64 | 24 | 122.66 | 1.02 | 1 |
| 1.64 | 1.74 | 2 | 5.9 | 4.26 |
| 25.19 | 27.48 | 34.74 | 1.02 | 2.26 |
| 298.44 | 375.65 | 484.32 | 183.71 | 140.79 |
| 82.46 | 55.33 | 138.64 | 1.02 | 14.3 |
| 579.87 | 690.74 | 560.25 | 823.42 | 781.26 |
| 1.64 | 1.74 | 2 | 1.02 | 1 |
| 1.64 | 1.74 | 4.76 | 1.02 | 1 |
| 93.91 | 225.94 | 859.98 | 1.02 | 1 |
| 1.64 | 1.74 | 2 | 1.02 | 1 |
| 1.64 | 1.74 | 2 | 1.02 | 1 |
| 1.64 | 1.74 | 2 | 1.02 | 1 |

| 3.92 | 1.74 | 2 | 1.02 | 2.26 |
| --- | --- | --- | --- | --- |
| 141.36 | 196.34 | 152.63 | 354.37 | 404.81 |
| 1.64 | 1.74 | 2 | 1.02 | 1 |
| 1.64 | 1.74 | 2 | 1.02 | 1 |
| 1.64 | 1.74 | 2 | 1.02 | 1 |
| 1.64 | 1.74 | 2 | 1.02 | 1 |
| 1.64 | 1.74 | 2 | 1.02 | 1 |
| 1.64 | 1.74 | 2 | 1.02 | 1 |
| 1.64 | 1.74 | 2 | 1.02 | 1 |
| 1.64 | 1.74 | 2 | 1.02 | 1 |
| 7.19 | 4.85 | 2 | 1.02 | 12.3 |
| 18.65 | 32.7 | 46.72 | 6.92 | 4.26 |
| 1.64 | 1.74 | 2 | 1.02 | 1 |
| 1638.51 | 2057.3 | 1974.96 | 988.97 | 1180.8 |
| 1.64 | 1.74 | 2 | 1.02 | 1 |
| 601.14 | 934.46 | 1077.78 | 168.38 | 160.87 |
| 1.64 | 1.74 | 2 | 1.02 | 1 |
| 1.64 | 17.04 | 40.73 | 1.02 | 1 |
| 223.18 | 306.02 | 318.48 | 138.75 | 104.65 |
| 506.24 | 635.03 | 692.13 | 815.24 | 833.46 |
| 1.64 | 1.74 | 2 | 1.02 | 1 |
| 1.64 | 1.74 | 2 | 11.01 | 9.28 |
| 1.64 | 1.74 | 2 | 1.02 | 1 |
| 712.41 | 742.97 | 678.15 | 571.01 | 605.58 |
| 483.33 | 520.14 | 424.38 | 608.82 | 565.43 |
| 1033.1 | 936.2 | 927.92 | 465.75 | 476.08 |
| 146.27 | 156.3 | 278.51 | 13.05 | 16.31 |
| 62.83 | 83.19 | 122.66 | 81.52 | 73.53 |
| 126.64 | 234.64 | 536.28 | 132.62 | 80.56 |
| 2.29 | 1.74 | 2 | 2.83 | 11.29 |
| 1.64 | 1.74 | 2 | 1.81 | 1 |
| 38.28 | 55.33 | 64.71 | 73.35 | 55.46 |
| 156.09 | 368.69 | 484.32 | 81.52 | 35.38 |
| 57.92 | 65.78 | 72.7 | 38.6 | 38.4 |
| 1069.1 | 2276.65 | 5307.91 | 394.22 | 305.42 |
| 1.64 | 1.74 | 2 | 1.02 | 1 |
| 175.73 | 173.71 | 124.65 | 220.5 | 162.88 |
| 26.83 | 32.7 | 82.69 | 20.21 | 16.31 |
| 548.78 | 561.92 | 572.24 | 666.05 | 652.76 |
| 1.64 | 11.81 | 28.74 | 1.02 | 1 |
| 188.82 | 231.16 | 334.46 | 203.13 | 205.04 |
| 2.29 | 17.04 | 18.75 | 1.02 | 1 |
| 1582.87 | 1703.91 | 1829.09 | 1577.58 | 1578.33 |
| 126.64 | 124.97 | 198.59 | 21.23 | 20.33 |
| 1.64 | 1.74 | 2 | 1.02 | 1 |
| 3899.76 | 4555.41 | 4422.72 | 3801.23 | 3690.46 |
| 1106.73 | 1265.22 | 1101.76 | 1214.81 | 1140.64 |
| 134.82 | 95.37 | 114.66 | 38.6 | 49.44 |

| 1.64 | 1.74 | 2 | 1.02 | 1 |
| --- | --- | --- | --- | --- |
| 1.64 | 1.74 | 2 | 1.02 | 6.27 |
| 316.44 | 412.21 | 404.4 | 391.16 | 329.52 |
| 1.64 | 1.74 | 2 | 1.02 | 1 |
| 1.64 | 1.74 | 2 | 1.02 | 1 |
| 1.64 | 1.74 | 2 | 1.02 | 1 |
| 1.64 | 1.74 | 2 | 1.02 | 1 |
| 1.64 | 1.74 | 2 | 1.02 | 1 |
| 1.64 | 1.74 | 2 | 1.02 | 1 |
| 1.64 | 1.74 | 2 | 1.02 | 1 |
| 1.64 | 1.74 | 2 | 1.02 | 1 |
| 1.64 | 1.74 | 2 | 1.02 | 1 |
| 1.64 | 1.74 | 2 | 1.02 | 1 |
| 1.64 | 1.74 | 2 | 1.02 | 1 |
| 1.64 | 1.74 | 2 | 1.02 | 1 |
| 1.64 | 1.74 | 2 | 1.02 | 1 |
| 107 | 130.19 | 98.68 | 96.85 | 100.64 |
| 36.65 | 41.41 | 38.73 | 7.94 | 15.31 |
| 51.37 | 69.26 | 68.7 | 46.78 | 22.33 |
| 201.9 | 205.05 | 174.61 | 134.66 | 236.16 |
| 1.64 | 8.33 | 98.68 | 9.99 | 2.26 |
| 1.64 | 1.74 | 2 | 2.83 | 3.26 |
| 1.64 | 1.74 | 2 | 1.02 | 1 |
| 1.64 | 8.33 | 28.74 | 1.02 | 1 |
| 126.64 | 201.56 | 374.42 | 1.02 | 5.27 |
| 475.15 | 507.95 | 652.17 | 211.3 | 267.28 |
| 1.64 | 1.74 | 2 | 1.02 | 1 |
| 1.64 | 3.11 | 2 | 9.99 | 9.28 |
| 1.64 | 1.74 | 2 | 1.02 | 1 |
| 1.64 | 1.74 | 2 | 1.02 | 1 |
| 146.27 | 194.6 | 272.52 | 109.11 | 83.57 |
| 1.64 | 1.74 | 2 | 1.02 | 1 |
| 421.16 | 523.62 | 428.38 | 391.16 | 392.76 |
| 75.92 | 112.78 | 86.69 | 67.21 | 40.4 |
| 159.36 | 158.04 | 176.61 | 214.37 | 176.93 |
| 1.64 | 1.74 | 2 | 1.02 | 1 |
| 552.06 | 751.67 | 572.24 | 463.71 | 516.24 |
| 959.47 | 1061.54 | 963.89 | 1062.54 | 1128.59 |
| 9921.05 | 9485.48 | 8980.55 | 9275.54 | 8282.14 |
| 92.28 | 121.49 | 96.68 | 128.53 | 108.67 |
| 804.03 | 857.86 | 893.95 | 1014.51 | 921.8 |
| 426.07 | 485.32 | 710.12 | 356.41 | 344.57 |
| 1.64 | 1.74 | 2 | 1.02 | 1 |
| 66.1 | 74.48 | 74.7 | 100.94 | 89.59 |
| 1.64 | 1.74 | 2 | 1.02 | 1 |
| 1.64 | 1.74 | 2 | 1.02 | 1 |
| 7088.75 | 9107.71 | 8710.79 | 6015.68 | 8075.35 |
| 5208.74 | 5565.1 | 6221.07 | 5328.97 | 5826.69 |

| 236.27 | 274.68 | 208.58 | 164.29 | 240.17 |
| --- | --- | --- | --- | --- |
| 182.27 | 144.12 | 146.63 | 124.44 | 139.79 |
| 1.64 | 1.74 | 2 | 1.02 | 1 |
| 25.19 | 97.11 | 122.66 | 1.02 | 1 |
| 31.74 | 13.55 | 50.72 | 5.9 | 7.28 |
| 237.9 | 339.09 | 266.52 | 228.67 | 221.1 |
| 123.37 | 151.08 | 308.48 | 69.26 | 83.57 |
| 6804.05 | 7439.99 | 7148.22 | 6088.24 | 6111.78 |
| 35.01 | 62.3 | 122.66 | 18.16 | 10.29 |
| 30.1 | 1.74 | 30.74 | 28.38 | 21.33 |
| 255.9 | 316.46 | 292.5 | 203.13 | 267.28 |
| 231.36 | 220.71 | 256.53 | 266.48 | 267.28 |
| 339.35 | 314.72 | 392.41 | 374.81 | 366.66 |
| 324.62 | 431.36 | 430.37 | 156.12 | 133.76 |
| 77.55 | 100.6 | 274.52 | 63.13 | 65.5 |
| 1021.65 | 1172.96 | 1483.41 | 382.98 | 457.01 |
| 254.26 | 288.61 | 416.39 | 169.4 | 130.75 |
| 7.19 | 17.04 | 28.74 | 1.02 | 1 |
| 1.64 | 3.11 | 2 | 2.83 | 1 |
| 311.53 | 300.79 | 376.42 | 343.13 | 300.4 |
| 170.82 | 130.19 | 190.59 | 206.19 | 186.97 |
| 1.64 | 1.74 | 2 | 1.02 | 1 |
| 1.64 | 1.74 | 2 | 1.02 | 1 |
| 1.64 | 1.74 | 2 | 1.02 | 8.28 |
| 1998.47 | 2130.42 | 2074.87 | 1840.21 | 2016.01 |
| 308.26 | 292.09 | 282.51 | 97.87 | 127.74 |
| 1.64 | 6.59 | 2 | 1.02 | 12.3 |
| 1.64 | 1.74 | 2 | 1.02 | 1 |
| 1.64 | 1.74 | 2 | 1.02 | 1 |
| 103.73 | 98.86 | 108.67 | 92.76 | 63.49 |
| 390.07 | 410.47 | 446.36 | 598.6 | 532.3 |
| 143 | 154.56 | 162.62 | 178.6 | 149.82 |
| 543.87 | 523.62 | 642.18 | 721.23 | 686.89 |
| 2571.15 | 2948.61 | 2612.38 | 2242.83 | 2419.57 |
| 836.76 | 852.64 | 853.99 | 996.12 | 925.81 |
| 1.64 | 1.74 | 2 | 1.02 | 1 |
| 1.64 | 1.74 | 2 | 1.02 | 1 |
| 1.64 | 1.74 | 2 | 1.02 | 1 |
| 13.74 | 10.07 | 24.74 | 42.69 | 48.43 |
| 321.35 | 309.5 | 338.46 | 67.21 | 30.36 |
| 283.72 | 429.62 | 470.34 | 291.01 | 256.23 |
| 152.82 | 170.23 | 148.63 | 158.16 | 141.79 |
| 12.1 | 30.96 | 22.75 | 50.86 | 39.4 |
| 2.29 | 1.74 | 6.76 | 4.88 | 2.26 |
| 10.47 | 1.74 | 2 | 1.02 | 1 |
| 1.64 | 1.74 | 2 | 1.02 | 1 |
| 165.91 | 171.97 | 240.55 | 188.82 | 162.88 |
| 1555.06 | 2133.9 | 1988.95 | 1103.42 | 2944.59 |

| 147.91 | 170.23 | 194.59 | 100.94 | 79.55 |
| --- | --- | --- | --- | --- |
| 1.64 | 1.74 | 2 | 1.02 | 1 |
| 3266.54 | 3228.89 | 3345.7 | 2764 | 2597.25 |
| 3673.96 | 3460.42 | 3519.55 | 2495.24 | 2448.68 |
| 31.74 | 74.48 | 84.69 | 23.27 | 15.31 |
| 4261.37 | 4170.69 | 4264.86 | 3791.01 | 3580.03 |
| 3914.49 | 3998.34 | 4134.98 | 3512.03 | 3311 |
| 8.83 | 3.11 | 38.73 | 31.45 | 7.28 |
| 1.64 | 1.74 | 2 | 1.02 | 1 |
| 378.62 | 318.2 | 532.28 | 287.94 | 160.87 |
| 1975.57 | 2008.56 | 1815.11 | 2119.18 | 1900.57 |
| 499.7 | 572.36 | 574.24 | 632.32 | 511.22 |
| 147.91 | 231.16 | 198.59 | 275.68 | 261.25 |
| 82.46 | 67.52 | 120.66 | 80.5 | 67.51 |
| 87.37 | 93.63 | 98.68 | 51.89 | 36.39 |
| 1481.43 | 1407.97 | 1405.48 | 1067.65 | 1091.45 |
| 1.64 | 1.74 | 2 | 1.02 | 1 |
| 1.64 | 1.74 | 2 | 1.02 | 1 |
| 354.07 | 393.06 | 510.3 | 373.78 | 396.78 |
| 250.99 | 250.31 | 332.46 | 120.35 | 92.6 |
| 144.64 | 180.67 | 154.63 | 155.1 | 133.76 |
| 219.9 | 177.19 | 114.66 | 54.95 | 33.38 |
| 1.64 | 1.74 | 2 | 1.02 | 1 |
| 1631.96 | 1132.92 | 2580.41 | 73.35 | 260.25 |
| 1347.26 | 920.53 | 2216.74 | 43.71 | 144.81 |
| 1.64 | 1.74 | 12.76 | 1.02 | 1 |
| 12.1 | 11.81 | 22.75 | 1.02 | 1 |
| 1.64 | 1.74 | 2 | 1.02 | 1 |
| 53.01 | 135.41 | 190.59 | 67.21 | 33.38 |
| 1.64 | 1.74 | 2 | 1.02 | 1 |
| 1.64 | 8.33 | 2 | 43.71 | 18.32 |
| 1291.63 | 1374.89 | 1389.5 | 1281.23 | 1104.5 |
| 1.64 | 1.74 | 2 | 1.02 | 1 |
| 1.64 | 1.74 | 2 | 1.02 | 1 |
| 1555.06 | 1703.91 | 1433.46 | 1236.27 | 1041.26 |
| 993.83 | 1136.4 | 1025.83 | 1370.13 | 1367.51 |
| 35.01 | 30.96 | 88.69 | 32.47 | 21.33 |
| 190.45 | 253.79 | 412.39 | 29.4 | 11.29 |
| 1.64 | 1.74 | 2 | 1.02 | 1 |
| 1414.34 | 1470.64 | 1505.39 | 1134.08 | 1081.41 |
| 421.16 | 603.7 | 742.09 | 440.21 | 392.76 |
| 313.17 | 509.69 | 502.31 | 317.58 | 305.42 |
| 2119.55 | 2734.49 | 3917.18 | 1051.3 | 1036.24 |
| 1.64 | 1.74 | 2 | 1.02 | 1 |
| 72.64 | 102.34 | 90.68 | 35.54 | 45.42 |
| 682.95 | 741.23 | 668.16 | 765.17 | 749.13 |
| 242.81 | 246.83 | 212.57 | 226.63 | 207.05 |
| 46.46 | 50.11 | 76.7 | 41.67 | 51.45 |

| 48.1 | 55.33 | 50.72 | 22.25 | 8.28 |
| --- | --- | --- | --- | --- |
| 84.1 | 81.45 | 100.68 | 55.97 | 86.58 |
| 179 | 293.83 | 548.27 | 66.19 | 39.4 |
| 188.82 | 316.46 | 274.52 | 171.45 | 150.83 |
| 779.49 | 807.38 | 716.11 | 585.32 | 525.27 |
| 1.64 | 1.74 | 2 | 1.02 | 1 |
| 7363.64 | 7718.52 | 6065.22 | 6898.6 | 5988.31 |
| 30.1 | 41.41 | 28.74 | 66.19 | 51.45 |
| 3721.41 | 3368.16 | 3749.34 | 3499.77 | 3287.91 |
| 157.73 | 208.53 | 218.57 | 213.35 | 167.89 |
| 110.28 | 109.3 | 132.65 | 223.56 | 198.01 |
| 376.98 | 403.5 | 388.41 | 333.93 | 318.47 |
| 128.27 | 151.08 | 496.31 | 118.31 | 82.57 |
| 509.51 | 593.25 | 476.33 | 654.81 | 548.36 |
| 437.52 | 487.06 | 584.23 | 304.29 | 341.56 |
| 8419 | 8947.56 | 8271.2 | 7711.01 | 7579.44 |
| 1.64 | 1.74 | 2 | 1.02 | 1 |
| 57.92 | 79.71 | 100.68 | 22.25 | 30.36 |
| 21.92 | 22.26 | 6.76 | 1.02 | 1.25 |
| 12.1 | 81.45 | 132.65 | 1.81 | 1 |
| 134.82 | 177.19 | 188.59 | 125.46 | 132.76 |
| 69.37 | 44.89 | 50.72 | 35.54 | 39.4 |
| 1.64 | 1.74 | 2 | 1.02 | 4.26 |
| 12.1 | 1.74 | 60.71 | 1.02 | 1 |
| 15.38 | 1.74 | 26.74 | 15.1 | 8.28 |
| 12.1 | 15.29 | 34.74 | 1.02 | 1 |
| 1.64 | 1.74 | 2 | 1.02 | 1 |
| 51.37 | 60.56 | 136.64 | 72.32 | 58.47 |
| 622.41 | 708.15 | 698.13 | 711.01 | 837.47 |
| 157.73 | 116.26 | 102.67 | 188.82 | 192.99 |
| 2.29 | 1.74 | 2 | 12.03 | 1 |
| 1.64 | 1.74 | 2 | 1.02 | 1 |
| 10.47 | 32.7 | 42.73 | 14.08 | 14.3 |
| 1.64 | 1.74 | 2 | 1.02 | 1 |
| 54.65 | 51.85 | 96.68 | 35.54 | 19.32 |
| 28.47 | 60.56 | 26.74 | 21.23 | 15.31 |
| 17.01 | 1.74 | 18.75 | 1.81 | 13.3 |
| 1.64 | 1.74 | 2 | 1.02 | 1 |
| 64.46 | 203.31 | 86.69 | 50.86 | 38.4 |
| 13.74 | 20.52 | 34.74 | 1.02 | 1 |
| 103.73 | 140.64 | 114.66 | 183.71 | 166.89 |
| 21.92 | 10.07 | 14.75 | 2.83 | 4.26 |
| 1.64 | 1.74 | 2 | 1.02 | 1 |
| 1.64 | 1.74 | 2 | 1.02 | 1 |
| 1.64 | 1.74 | 2 | 1.02 | 1 |
| 1.64 | 1.74 | 2 | 1.02 | 1 |
| 3817.95 | 4087.13 | 4083.03 | 3502.83 | 3496.71 |
| 1.64 | 11.81 | 2.76 | 6.92 | 9.28 |

| 56.28 | 51.85 | 74.7 | 40.64 | 30.36 |
| --- | --- | --- | --- | --- |
| 393.34 | 457.47 | 382.42 | 624.15 | 583.49 |
| 64.46 | 55.33 | 26.74 | 43.71 | 44.42 |
| 30.1 | 36.18 | 42.73 | 73.35 | 65.5 |
| 419.52 | 466.17 | 464.34 | 452.47 | 475.08 |
| 12.1 | 4.85 | 48.72 | 1.02 | 1 |
| 113.55 | 114.52 | 110.67 | 167.36 | 154.84 |
| 126.64 | 97.11 | 138.64 | 167.36 | 150.83 |
| 1.64 | 1.74 | 2 | 1.02 | 1 |
| 36.65 | 46.63 | 58.71 | 57 | 62.49 |
| 1316.17 | 1477.6 | 1671.24 | 1118.75 | 993.07 |
| 2726.59 | 2791.94 | 2792.21 | 2508.53 | 2465.74 |
| 406.43 | 601.96 | 446.36 | 468.82 | 370.68 |
| 514.42 | 645.48 | 616.2 | 374.81 | 314.46 |
| 2173.55 | 2602.19 | 2786.22 | 2832.47 | 2678.56 |
| 1.64 | 1.74 | 2 | 1.02 | 1 |
| 1.64 | 1.74 | 2 | 1.02 | 1 |
| 26.83 | 27.48 | 20.75 | 9.99 | 20.33 |
| 195.36 | 192.86 | 224.56 | 125.46 | 136.77 |
| 1941.21 | 2015.52 | 1753.16 | 1641.96 | 1666.67 |
| 570.05 | 527.1 | 656.17 | 432.03 | 475.08 |
| 3606.88 | 3937.41 | 4132.98 | 2251.01 | 2198.72 |
| 846.58 | 936.2 | 861.98 | 800.94 | 961.95 |
| 79771.26 | 84312.12 | 87948.27 | 82850.14 | 88204.92 |
| 180.63 | 163.27 | 168.61 | 197 | 184.96 |
| 1496.15 | 1594.24 | 1533.36 | 1790.13 | 1959.79 |
| 11266.02 | 11107.94 | 11408.33 | 10276.99 | 10731.57 |
| 159.36 | 119.75 | 118.66 | 176.56 | 129.75 |
| 1803.76 | 1970.26 | 2114.83 | 1636.85 | 1534.16 |
| 18206.86 | 19434.39 | 18124.18 | 32046.48 | 31374.07 |
| 13720.35 | 14023.85 | 13965.98 | 10926.92 | 10574.97 |
| 4907.67 | 5126.41 | 5094.1 | 4312.18 | 4443.36 |
| 344.26 | 293.83 | 274.52 | 634.37 | 585.5 |
| 624.05 | 697.71 | 594.22 | 494.37 | 540.33 |

| 3Young Con 48h | 1Young Repop 48h | 2Young Repop 48h | 3Young Repop 48h | 1Aged Con 48h |
| --- | --- | --- | --- | --- |
| 335.26 | 347.78 | 343.88 | 387.12 | 299.7 |
| 821.46 | 1085.37 | 962 | 1064.43 | 1234.74 |
| 430.89 | 484.37 | 453.59 | 466.32 | 442.46 |
| 151.04 | 150.92 | 163.82 | 192.53 | 145.6 |
| 461.09 | 432.14 | 390.79 | 390.14 | 420.72 |
| 121.85 | 113.16 | 129.02 | 143.5 | 121.01 |
| 1.01 | 1 | 1 | 1 | 1 |
| 1.01 | 1 | 1 | 1 | 1 |
| 37827.18 | 35238.83 | 38130.27 | 39133.77 | 30530 |
| 262.78 | 363.85 | 349.18 | 348.66 | 529.44 |
| 1545.23 | 1420.42 | 1375.09 | 1354.05 | 1326.44 |
| 126.88 | 110.75 | 93.46 | 98.25 | 115.34 |
| 4624.51 | 8556.92 | 7571.44 | 8370.72 | 9682.2 |
| 1.01 | 1 | 1 | 1 | 4.73 |
| 15.15 | 20.76 | 7.21 | 18.3 | 9.45 |
| 414.78 | 393.58 | 415.76 | 370.53 | 381.01 |
| 1808.96 | 1842.25 | 1735.98 | 1888.81 | 1636.55 |
| 192.32 | 199.94 | 198.62 | 234.01 | 189.09 |
| 1.01 | 1 | 1 | 1 | 1 |
| 391.63 | 445 | 429.38 | 339.61 | 340.35 |
| 226.54 | 177.44 | 150.96 | 181.97 | 197.59 |
| 129.9 | 126.82 | 119.18 | 134.45 | 128.58 |
| 1.01 | 1 | 1 | 1 | 10.4 |
| 65.48 | 211.99 | 194.84 | 208.37 | 251.48 |
| 15.15 | 15.14 | 2.67 | 13.02 | 8.51 |
| 19.18 | 12.73 | 1.15 | 23.58 | 25.52 |
| 1.01 | 1 | 1 | 1 | 1 |
| 1.01 | 1 | 1 | 1 | 1 |
| 627.18 | 1306.33 | 1084.57 | 1276.37 | 2048.76 |
| 1125.46 | 1888.85 | 1665.62 | 1769.64 | 2939.36 |
| 1829.1 | 1839.03 | 1640.65 | 1765.11 | 1721.64 |
| 31.25 | 29.6 | 34.44 | 27.35 | 75.63 |
| 29.24 | 33.62 | 33.69 | 31.12 | 80.36 |
| 32.26 | 40.85 | 46.55 | 26.6 | 52.94 |
| 22.2 | 48.88 | 16.29 | 43.94 | 107.78 |
| 284.93 | 553.47 | 479.31 | 603.59 | 2100.76 |
| 1.01 | 1 | 1 | 1 | 1 |
| 1.06 | 1 | 1 | 1 | 1 |
| 12.13 | 2.28 | 1.15 | 4.72 | 10.4 |
| 1.01 | 1 | 1 | 1 | 1 |
| 1.01 | 1 | 1 | 1 | 1 |
| 5.08 | 1 | 2.67 | 6.23 | 9.45 |
| 1.01 | 1 | 1 | 1 | 1 |
| 1.01 | 1 | 1 | 1 | 1 |
| 32.26 | 30.4 | 23.85 | 41.68 | 42.54 |
| 40.31 | 73.79 | 49.58 | 52.24 | 138.98 |
| 191.31 | 211.19 | 206.94 | 209.12 | 194.76 |

| 350.36 | 330.9 | 293.95 | 283.79 | 255.27 |
| --- | --- | --- | --- | --- |
| 52.39 | 44.87 | 48.06 | 67.33 | 99.27 |
| 13.14 | 7.1 | 17.04 | 25.84 | 32.14 |
| 1.01 | 1 | 1 | 1 | 8.51 |
| 3.07 | 20.76 | 5.69 | 31.12 | 65.23 |
| 20.18 | 44.06 | 24.61 | 27.35 | 34.98 |
| 1.01 | 3.08 | 1 | 3.22 | 22.69 |
| 1.01 | 1 | 1 | 1 | 1 |
| 1.06 | 1 | 1 | 3.97 | 1 |
| 1.01 | 1 | 1 | 1 | 1 |
| 129.9 | 133.25 | 121.45 | 150.29 | 144.65 |
| 1.01 | 1 | 1 | 1 | 1 |
| 1.01 | 1 | 1 | 5.48 | 27.42 |
| 1.01 | 1 | 1 | 1 | 1 |
| 1.01 | 31.21 | 26.88 | 25.84 | 46.32 |
| 6.09 | 18.35 | 6.45 | 21.32 | 20.8 |
| 199.36 | 191.1 | 188.03 | 188 | 185.3 |
| 1.01 | 19.15 | 18.56 | 16.79 | 54.83 |
| 37.29 | 68.97 | 63.19 | 54.5 | 56.72 |
| 3.07 | 1 | 1 | 1 | 2.83 |
| 1.01 | 1 | 5.69 | 1 | 3.78 |
| 1.01 | 1 | 1 | 1 | 1 |
| 1.01 | 1 | 1 | 1 | 1 |
| 58.43 | 47.28 | 45.04 | 37.16 | 82.25 |
| 1.01 | 1 | 1 | 1 | 1 |
| 1.01 | 1 | 1 | 1 | 1 |
| 1.06 | 1 | 1 | 1 | 1 |
| 1.06 | 1 | 1 | 1 | 1 |
| 11.12 | 1.48 | 1 | 1 | 1 |
| 10.12 | 15.94 | 17.8 | 14.53 | 21.74 |
| 39.31 | 36.03 | 25.36 | 24.33 | 78.47 |
| 29.24 | 36.03 | 27.63 | 27.35 | 38.76 |
| 1.01 | 1 | 1 | 1 | 1 |
| 22.2 | 9.51 | 1 | 13.02 | 32.14 |
| 1759.64 | 1769.93 | 1658.81 | 1696.48 | 1928.69 |
| 1.01 | 1 | 1 | 1 | 1 |
| 53.4 | 36.83 | 16.29 | 74.87 | 41.6 |
| 1.01 | 1 | 1 | 1 | 1 |
| 1.06 | 1 | 1 | 1 | 1 |
| 1.06 | 1 | 1 | 1 | 18.91 |
| 1.01 | 1 | 1 | 1 | 1 |
| 1.01 | 1.48 | 1 | 1 | 29.31 |
| 1.01 | 1 | 1 | 1 | 1 |
| 233.59 | 186.28 | 180.46 | 220.44 | 271.34 |
| 1.01 | 1 | 1 | 1 | 1 |
| 43.33 | 60.94 | 33.69 | 50.73 | 75.63 |
| 6.09 | 1 | 1 | 1 | 1 |
| 1605.62 | 1993.3 | 1782.89 | 1785.48 | 2328.61 |

| 12.13 | 12.73 | 11.75 | 4.72 | 30.25 |
| --- | --- | --- | --- | --- |
| 10.12 | 3.89 | 1 | 1 | 1 |
| 1.01 | 1 | 1 | 1 | 1 |
| 94.67 | 114.77 | 110.1 | 120.12 | 91.71 |
| 92.66 | 79.42 | 108.59 | 211.39 | 89.81 |
| 8.1 | 2.28 | 1.15 | 5.48 | 2.83 |
| 1.01 | 1 | 1 | 1 | 1 |
| 134.94 | 161.37 | 129.02 | 123.14 | 161.67 |
| 1.01 | 1 | 1 | 1 | 1 |
| 14.14 | 42.46 | 30.66 | 28.11 | 84.14 |
| 1.01 | 1 | 1 | 1 | 3.78 |
| 4.08 | 3.89 | 5.69 | 6.99 | 5.67 |
| 105.75 | 89.06 | 87.4 | 86.94 | 86.98 |
| 9.11 | 22.37 | 1 | 16.04 | 17.96 |
| 21.19 | 7.1 | 10.23 | 5.48 | 22.69 |
| 1.01 | 1 | 1 | 1 | 1 |
| 11.12 | 13.53 | 1.91 | 11.51 | 5.67 |
| 202.38 | 355.81 | 252.34 | 211.39 | 382.9 |
| 1.01 | 1 | 1 | 1 | 1 |
| 1.01 | 1 | 1 | 1 | 2.83 |
| 6.09 | 1 | 6.45 | 12.27 | 10.4 |
| 15114.59 | 14471.32 | 13751.91 | 14652.75 | 14450.04 |
| 348.34 | 385.54 | 316.65 | 415.78 | 539.84 |
| 914.07 | 775.23 | 608.68 | 943.75 | 784.71 |
| 21.19 | 49.69 | 49.58 | 43.19 | 43.49 |
| 4.08 | 1 | 1 | 1 | 1 |
| 1.01 | 1 | 1 | 1 | 1 |
| 1362.02 | 1397.92 | 1308.52 | 1348.77 | 1849.27 |
| 1.01 | 1 | 1 | 1 | 1 |
| 139.97 | 194.31 | 157.01 | 188.76 | 198.54 |
| 157.08 | 220.83 | 194.08 | 222.7 | 145.6 |
| 123.86 | 171.01 | 137.34 | 140.49 | 192.87 |
| 467.13 | 441.78 | 405.17 | 437.66 | 381.95 |
| 19.18 | 11.12 | 5.69 | 15.28 | 30.25 |
| 170.17 | 156.55 | 156.25 | 165.38 | 136.14 |
| 1.01 | 1 | 1 | 1 | 10.4 |
| 1.01 | 1 | 1 | 1 | 1 |
| 360.42 | 444.19 | 419.54 | 362.23 | 863.18 |
| 1.01 | 1 | 1 | 1 | 1 |
| 203.39 | 228.06 | 206.19 | 169.15 | 183.41 |
| 894.94 | 915.03 | 836.41 | 929.42 | 925.58 |
| 1.01 | 1 | 1 | 1 | 1 |
| 29.24 | 29.6 | 36.71 | 28.11 | 24.58 |
| 1.01 | 1 | 1 | 1 | 1 |
| 1.01 | 1 | 1 | 1 | 1 |
| 18.17 | 37.63 | 36.71 | 44.7 | 53.89 |
| 23035.76 | 23395.56 | 22684.03 | 21784.06 | 25728.14 |
| 47.36 | 65.76 | 50.33 | 59.78 | 85.09 |

| 1.01 | 1 | 1 | 1 | 1 |
| --- | --- | --- | --- | --- |
| 189.3 | 178.24 | 179.71 | 184.23 | 173.96 |
| 561.75 | 598.46 | 554.97 | 619.43 | 771.47 |
| 414.78 | 488.39 | 427.86 | 451.99 | 706.24 |
| 859.71 | 1179.38 | 1104.24 | 1264.3 | 1243.25 |
| 1.01 | 1 | 1 | 1 | 1 |
| 1.01 | 4.69 | 1.91 | 1 | 9.45 |
| 18.17 | 70.58 | 49.58 | 45.45 | 68.07 |
| 1.01 | 1 | 1 | 1 | 1 |
| 7806.46 | 7137.17 | 7150.03 | 6809.45 | 7138.04 |
| 184.26 | 205.56 | 180.46 | 200.83 | 256.21 |
| 1.01 | 1 | 1 | 1 | 1 |
| 1791.85 | 3491.79 | 3206.76 | 3665.03 | 4633.58 |
| 385.59 | 382.33 | 406.68 | 369.02 | 360.21 |
| 2782.37 | 2677.06 | 2988.11 | 3156.68 | 2104.54 |
| 485.24 | 507.67 | 524.7 | 531.94 | 774.31 |
| 1.01 | 1 | 1 | 1 | 1 |
| 5.08 | 9.51 | 7.96 | 15.28 | 23.63 |
| 1.01 | 1 | 1 | 1 | 1 |
| 1216.06 | 1324 | 1123.16 | 1078.76 | 2423.15 |
| 1.01 | 4.69 | 1 | 1 | 47.27 |
| 1.01 | 1 | 1 | 1 | 1 |
| 1.01 | 1 | 1 | 1 | 1 |
| 1.01 | 4.69 | 12.5 | 10 | 6.62 |
| 1.01 | 1 | 1 | 1 | 1 |
| 1.01 | 1 | 1 | 1 | 1 |
| 1.01 | 1 | 1 | 1 | 1 |
| 50.38 | 69.77 | 47.31 | 37.91 | 80.36 |
| 1.01 | 1 | 1 | 1 | 1 |
| 1.01 | 1 | 1 | 1 | 1 |
| 22.2 | 40.85 | 33.69 | 37.91 | 114.4 |
| 116.82 | 174.23 | 148.69 | 199.32 | 320.5 |
| 14.14 | 11.12 | 12.5 | 17.55 | 30.25 |
| 1.01 | 1 | 1 | 1 | 1 |
| 17.16 | 21.56 | 10.99 | 21.32 | 19.85 |
| 45.35 | 46.47 | 39.74 | 37.91 | 52 |
| 131.92 | 203.15 | 206.94 | 193.28 | 325.23 |
| 144 | 208.78 | 169.87 | 209.88 | 277.01 |
| 87.63 | 82.63 | 55.63 | 3.97 | 91.71 |
| 334.25 | 366.26 | 325.73 | 328.29 | 393.3 |
| 27.23 | 31.21 | 33.69 | 26.6 | 34.98 |
| 24.21 | 17.55 | 14.77 | 21.32 | 23.63 |
| 6.09 | 1 | 1 | 3.22 | 1 |
| 1.01 | 1 | 1 | 1 | 1.89 |
| 1.01 | 1 | 1 | 1 | 1 |
| 63.47 | 105.93 | 91.19 | 98.25 | 157.89 |
| 67.49 | 76.2 | 101.02 | 118.61 | 186.25 |
| 146.01 | 248.15 | 236.45 | 210.63 | 792.27 |

| 88.63 | 143.69 | 157.01 | 154.06 | 250.54 |
| --- | --- | --- | --- | --- |
| 1.06 | 31.21 | 26.12 | 51.49 | 92.65 |
| 276.87 | 310.01 | 318.16 | 276.25 | 411.26 |
| 200.37 | 200.74 | 244.02 | 219.68 | 189.09 |
| 521.48 | 830.67 | 700.99 | 713.71 | 901 |
| 1.01 | 1 | 1 | 1 | 1 |
| 3.07 | 1 | 1 | 1 | 1 |
| 688.58 | 649.08 | 646.51 | 633 | 579.55 |
| 3751.76 | 3527.14 | 3403.47 | 3253.22 | 2968.67 |
| 88.63 | 75.4 | 29.9 | 27.35 | 41.6 |
| 1.01 | 1 | 1 | 1 | 1 |
| 17.16 | 15.94 | 7.21 | 6.23 | 21.74 |
| 110.78 | 102.72 | 98.75 | 80.15 | 63.34 |
| 207.41 | 168.6 | 163.06 | 185.74 | 183.41 |
| 3.07 | 1 | 1 | 1 | 1.89 |
| 1.01 | 1 | 1 | 1 | 1 |
| 1.01 | 4.69 | 1.91 | 5.48 | 1 |
| 2.06 | 1 | 1 | 1 | 1 |
| 109.77 | 153.34 | 90.43 | 79.39 | 159.78 |
| 46.35 | 70.58 | 54.11 | 34.14 | 67.12 |
| 238.62 | 285.11 | 204.67 | 267.95 | 231.63 |
| 1.01 | 1 | 3.42 | 7.74 | 12.29 |
| 1.01 | 1 | 1 | 1 | 1 |
| 33.27 | 44.06 | 34.44 | 38.66 | 38.76 |
| 235.6 | 687.65 | 557.24 | 706.92 | 652.35 |
| 2.06 | 3.08 | 1 | 2.46 | 1 |
| 1.01 | 1 | 1 | 1 | 1 |
| 1.01 | 1 | 1 | 1 | 1 |
| 1.01 | 1 | 1 | 1 | 1 |
| 1.01 | 1 | 1 | 1 | 1 |
| 1.01 | 1 | 1 | 1 | 1 |
| 185.27 | 207.17 | 194.84 | 180.46 | 324.28 |
| 987.55 | 1115.9 | 1132.23 | 1075.74 | 1104.27 |
| 4.08 | 6.3 | 2.67 | 10.76 | 14.18 |
| 25.21 | 40.04 | 20.07 | 31.12 | 63.34 |
| 122.86 | 130.84 | 112.37 | 142 | 280.79 |
| 16.16 | 8.71 | 5.69 | 10.76 | 10.4 |
| 667.44 | 662.74 | 727.47 | 674.49 | 553.08 |
| 1.01 | 1 | 1 | 1 | 1 |
| 128.9 | 134.05 | 132.04 | 115.6 | 328.06 |
| 1.01 | 7.91 | 4.94 | 10.76 | 29.31 |
| 54.41 | 89.86 | 83.62 | 99.76 | 147.49 |
| 126.88 | 187.88 | 165.33 | 194.04 | 265.67 |
| 256.74 | 315.64 | 267.47 | 270.97 | 378.17 |
| 123.86 | 189.49 | 157.77 | 146.52 | 242.98 |
| 1.01 | 1 | 1 | 1 | 1 |
| 1.01 | 1 | 1 | 1 | 1 |
| 1592.54 | 1651.02 | 1518.09 | 1485.29 | 1571.31 |

| 379.55 | 382.33 | 408.19 | 381.09 | 455.7 |
| --- | --- | --- | --- | --- |
| 1.01 | 1 | 1 | 1 | 1 |
| 1.01 | 1 | 1 | 1 | 1 |
| 446.99 | 473.12 | 436.19 | 448.22 | 603.19 |
| 913.06 | 904.59 | 918.12 | 958.83 | 784.71 |
| 313.11 | 358.22 | 318.92 | 325.27 | 307.26 |
| 660.4 | 618.55 | 607.93 | 669.96 | 512.42 |
| 377.54 | 338.14 | 356.74 | 320 | 347.92 |
| 25.21 | 25.58 | 6.45 | 16.04 | 25.52 |
| 329.22 | 321.26 | 331.78 | 343.38 | 351.7 |
| 18.17 | 8.71 | 14.02 | 11.51 | 5.67 |
| 129.9 | 130.84 | 142.63 | 105.04 | 135.2 |
| 1.01 | 1 | 1 | 1 | 1 |
| 272.85 | 224.04 | 244.77 | 210.63 | 212.72 |
| 1.01 | 1 | 1 | 1 | 1 |
| 107.76 | 107.54 | 116.15 | 135.21 | 104 |
| 275.87 | 299.57 | 267.47 | 290.58 | 464.21 |
| 21.19 | 19.96 | 10.23 | 14.53 | 12.29 |
| 27.23 | 20.76 | 4.18 | 29.61 | 25.52 |
| 1.01 | 1 | 1 | 1 | 1 |
| 1.01 | 1 | 1 | 1 | 1 |
| 1.01 | 1 | 1 | 1 | 1 |
| 1.01 | 1 | 1 | 1 | 1.89 |
| 158.09 | 208.78 | 200.89 | 196.3 | 245.81 |
| 1.01 | 2.28 | 1 | 3.97 | 1.89 |
| 22.2 | 17.55 | 24.61 | 28.11 | 26.47 |
| 202.38 | 306.8 | 302.27 | 133.7 | 232.58 |
| 1.01 | 1 | 1 | 1 | 1 |
| 1.01 | 1 | 1 | 1 | 1 |
| 1.06 | 3.89 | 1 | 1 | 1 |
| 234.59 | 257.79 | 222.83 | 242.31 | 225.96 |
| 34.27 | 20.76 | 23.1 | 26.6 | 29.31 |
| 3.07 | 2.28 | 1 | 1 | 7.56 |
| 1094.26 | 1032.34 | 1014.97 | 1052.36 | 994.6 |
| 1.01 | 1 | 1 | 1 | 1 |
| 1.01 | 1 | 1 | 1 | 1 |
| 1.01 | 1 | 1 | 1 | 1 |
| 1.06 | 1 | 1 | 2.46 | 17.02 |
| 4.08 | 1 | 1 | 1 | 7.56 |
| 120.84 | 125.21 | 129.77 | 87.69 | 150.32 |
| 1.01 | 5.5 | 1 | 5.48 | 10.4 |
| 816.43 | 785.67 | 753.95 | 814.02 | 690.17 |
| 2.06 | 1 | 1 | 1 | 1 |
| 1.01 | 1 | 1 | 1 | 2.83 |
| 1.01 | 1 | 1 | 1 | 1 |
| 1.01 | 1 | 1 | 1 | 1 |
| 1.01 | 1 | 1 | 1 | 1 |
| 1.01 | 1 | 1 | 1 | 1 |

| 10.12 | 3.89 | 1.91 | 1 | 5.67 |
| --- | --- | --- | --- | --- |
| 221.51 | 204.76 | 220.56 | 89.2 | 242.03 |
| 1.01 | 1 | 1 | 1 | 1 |
| 1.01 | 1 | 1 | 1 | 1 |
| 1.01 | 1 | 1 | 1 | 1 |
| 1.01 | 1 | 1 | 1 | 1 |
| 1.01 | 1 | 1 | 1 | 1 |
| 4.08 | 1 | 1 | 2.46 | 1 |
| 1.01 | 1 | 1 | 1 | 1 |
| 1.01 | 1 | 1 | 1 | 1 |
| 5.08 | 2.28 | 7.21 | 3.97 | 11.34 |
| 3.07 | 14.33 | 6.45 | 13.02 | 50.11 |
| 1.01 | 1 | 1 | 1 | 1 |
| 1132.51 | 1210.71 | 1077.76 | 1347.27 | 1247.03 |
| 1.01 | 1 | 1 | 1 | 1 |
| 134.94 | 154.14 | 128.26 | 125.4 | 228.79 |
| 1.01 | 1 | 1 | 1 | 1 |
| 1.01 | 1 | 1 | 1 | 1 |
| 107.76 | 120.39 | 91.19 | 108.05 | 107.78 |
| 799.31 | 787.28 | 704.77 | 778.57 | 917.07 |
| 1.01 | 1 | 1 | 1 | 1 |
| 3.07 | 1 | 7.21 | 13.78 | 4.73 |
| 1.01 | 1 | 1 | 1 | 1 |
| 544.64 | 600.87 | 606.41 | 590.01 | 615.48 |
| 520.48 | 649.08 | 623.82 | 573.42 | 586.17 |
| 468.13 | 486.78 | 477.8 | 491.96 | 390.46 |
| 31.25 | 38.44 | 26.88 | 23.58 | 66.18 |
| 91.65 | 89.86 | 62.44 | 100.51 | 142.76 |
| 100.71 | 111.55 | 101.78 | 114.09 | 173.01 |
| 23.2 | 16.74 | 4.94 | 7.74 | 9.45 |
| 7.1 | 1 | 1 | 1 | 4.73 |
| 50.38 | 56.11 | 72.27 | 59.78 | 71.85 |
| 19.18 | 85.04 | 80.59 | 99 | 81.31 |
| 35.28 | 57.72 | 35.2 | 57.52 | 85.09 |
| 253.72 | 391.16 | 352.96 | 394.66 | 693.95 |
| 1.01 | 1 | 1 | 1 | 1 |
| 180.24 | 191.9 | 154.74 | 194.79 | 184.36 |
| 23.2 | 14.33 | 10.23 | 18.3 | 19.85 |
| 605.03 | 762.37 | 660.89 | 761.22 | 693.95 |
| 1.01 | 9.51 | 4.94 | 1 | 7.56 |
| 185.27 | 282.7 | 251.58 | 267.2 | 346.97 |
| 3.07 | 9.51 | 7.96 | 16.79 | 16.07 |
| 1507.98 | 1537.73 | 1305.49 | 1419.67 | 1714.07 |
| 17.16 | 45.67 | 38.23 | 40.17 | 55.78 |
| 1.01 | 1 | 1 | 1 | 1 |
| 3679.28 | 3368.05 | 3244.59 | 3278.11 | 3370.48 |
| 1154.65 | 1031.54 | 1083.06 | 1113.45 | 1145.87 |
| 40.31 | 72.18 | 38.98 | 83.16 | 61.45 |

| 1.01 | 1 | 1 | 1 | 1 |
| --- | --- | --- | --- | --- |
| 2.06 | 1 | 1 | 1 | 1 |
| 368.48 | 325.28 | 306.81 | 221.94 | 333.74 |
| 1.01 | 1 | 1 | 1 | 1 |
| 1.01 | 1 | 1 | 1 | 1 |
| 1.01 | 1 | 1 | 1 | 1 |
| 1.01 | 1 | 1 | 1 | 1 |
| 1.01 | 1 | 1 | 1 | 1 |
| 1.01 | 1 | 1 | 1 | 1 |
| 1.01 | 1 | 1 | 1 | 1 |
| 1.01 | 1 | 1 | 1 | 1 |
| 1.01 | 1 | 1 | 1 | 1 |
| 1.01 | 1 | 1 | 1 | 1 |
| 1.01 | 1 | 1 | 1 | 1 |
| 1.01 | 1 | 1 | 1 | 1 |
| 1.01 | 1 | 1 | 1 | 1 |
| 101.72 | 107.54 | 98.75 | 108.81 | 141.81 |
| 14.14 | 15.94 | 19.31 | 15.28 | 12.29 |
| 21.19 | 22.37 | 34.44 | 21.32 | 29.31 |
| 160.1 | 226.45 | 203.16 | 253.62 | 182.47 |
| 5.08 | 3.08 | 1 | 1 | 1 |
| 1.01 | 1 | 1 | 3.22 | 13.23 |
| 1.01 | 1 | 1 | 1 | 1 |
| 1.01 | 1.48 | 1 | 1 | 12.29 |
| 12.13 | 11.12 | 9.48 | 1 | 103.05 |
| 262.78 | 249.75 | 210.73 | 281.53 | 290.25 |
| 1.01 | 1 | 1 | 1 | 1 |
| 11.12 | 7.91 | 3.42 | 2.46 | 6.62 |
| 1.01 | 1 | 1 | 1 | 1 |
| 1.01 | 1 | 1 | 1 | 1 |
| 69.51 | 101.91 | 83.62 | 69.59 | 92.65 |
| 1.01 | 1 | 1 | 1 | 1 |
| 300.02 | 516.51 | 457.37 | 498 | 741.22 |
| 59.44 | 64.15 | 51.09 | 71.85 | 61.45 |
| 196.34 | 201.54 | 189.54 | 167.64 | 183.41 |
| 1.01 | 1 | 1 | 1 | 1 |
| 510.41 | 548.65 | 481.58 | 622.44 | 525.66 |
| 1144.59 | 1085.37 | 1007.4 | 1078.76 | 919.91 |
| 8806.05 | 7752.63 | 8250.85 | 7562.93 | 7371.56 |
| 120.84 | 133.25 | 103.29 | 140.49 | 79.41 |
| 849.64 | 1002.61 | 962 | 898.49 | 864.13 |
| 422.83 | 408.84 | 396.84 | 442.94 | 385.74 |
| 1.01 | 1 | 1 | 1 | 1 |
| 81.59 | 81.83 | 82.11 | 92.97 | 61.45 |
| 1.01 | 1 | 1 | 1 | 1 |
| 1.01 | 1 | 1 | 1 | 1 |
| 8264.48 | 8827.69 | 7258.22 | 10543.67 | 7861.29 |
| 6248.2 | 6251.74 | 6280.73 | 6603.54 | 4955.97 |

| 226.54 | 202.35 | 178.19 | 295.11 | 183.41 |
| --- | --- | --- | --- | --- |
| 146.01 | 113.96 | 97.24 | 115.6 | 222.18 |
| 1.01 | 1 | 1 | 1 | 1 |
| 1.01 | 5.5 | 1 | 1 | 21.74 |
| 7.1 | 4.69 | 4.18 | 2.46 | 3.78 |
| 217.48 | 247.34 | 234.18 | 283.04 | 226.9 |
| 79.57 | 68.97 | 75.3 | 86.94 | 90.76 |
| 6556.23 | 6399.58 | 6213.39 | 6237.74 | 5532.69 |
| 12.13 | 33.62 | 6.45 | 18.3 | 40.65 |
| 23.2 | 32.01 | 23.85 | 20.56 | 35.92 |
| 256.74 | 193.51 | 199.38 | 241.55 | 281.74 |
| 225.53 | 258.59 | 250.82 | 233.26 | 289.3 |
| 395.65 | 325.28 | 318.92 | 275.5 | 282.68 |
| 141.98 | 123.61 | 147.17 | 143.5 | 139.92 |
| 40.31 | 72.99 | 58.65 | 72.61 | 89.81 |
| 368.48 | 461.07 | 427.86 | 510.06 | 232.58 |
| 116.82 | 122 | 149.44 | 136.72 | 135.2 |
| 1.01 | 2.28 | 1 | 6.23 | 5.67 |
| 9.11 | 7.91 | 1 | 4.72 | 1.89 |
| 365.46 | 380.72 | 323.46 | 351.67 | 297.81 |
| 225.53 | 222.43 | 213.75 | 206.11 | 252.43 |
| 1.01 | 1 | 1 | 1 | 1 |
| 1.01 | 1 | 1 | 1 | 1 |
| 22.2 | 1 | 1 | 11.51 | 11.34 |
| 2026.39 | 1864.74 | 1658.81 | 1885.79 | 1955.16 |
| 104.74 | 102.72 | 101.78 | 117.11 | 117.23 |
| 3.07 | 3.08 | 1 | 6.99 | 1 |
| 1.01 | 1 | 1 | 1 | 2.83 |
| 1.01 | 1 | 1 | 1 | 1 |
| 79.57 | 88.25 | 98.75 | 93.72 | 100.21 |
| 658.39 | 609.71 | 565.56 | 577.94 | 513.37 |
| 138.96 | 199.13 | 153.23 | 217.42 | 189.09 |
| 569.8 | 762.37 | 700.23 | 650.35 | 1239.46 |
| 2373.68 | 2380.58 | 2255.75 | 2375.29 | 2156.54 |
| 1012.72 | 991.36 | 987.73 | 970.9 | 831.04 |
| 1.01 | 1 | 1 | 1 | 1 |
| 1.01 | 1 | 1 | 1 | 1 |
| 1.01 | 1 | 1 | 1 | 1 |
| 16.16 | 36.83 | 30.66 | 43.19 | 53.89 |
| 39.31 | 66.56 | 42.77 | 53 | 57.67 |
| 272.85 | 259.39 | 297.73 | 240.8 | 269.45 |
| 132.92 | 187.08 | 132.8 | 148.03 | 139.92 |
| 49.37 | 58.52 | 37.47 | 58.28 | 38.76 |
| 4.08 | 1 | 1 | 1 | 46.32 |
| 4.08 | 1.48 | 1 | 3.97 | 9.45 |
| 1.01 | 1 | 1 | 1 | 1 |
| 169.16 | 134.86 | 125.99 | 114.09 | 177.74 |
| 773.14 | 1462.2 | 2001.54 | 2223.69 | 805.51 |

| 102.73 | 92.27 | 107.07 | 128.42 | 70.91 |
| --- | --- | --- | --- | --- |
| 1.01 | 1 | 1 | 1 | 1 |
| 2613.26 | 2507.53 | 2497.85 | 2525.38 | 2325.77 |
| 2431.06 | 2272.11 | 2110.49 | 2247.07 | 2217.04 |
| 12.13 | 51.29 | 48.82 | 44.7 | 88.87 |
| 3624.92 | 3433.94 | 3144.72 | 3445.55 | 3265.53 |
| 3335.01 | 3022.56 | 2904.89 | 2968.87 | 2940.3 |
| 28.23 | 24.78 | 22.34 | 18.3 | 48.22 |
| 1.01 | 1 | 1 | 1 | 1 |
| 263.79 | 236.09 | 251.58 | 209.88 | 241.08 |
| 1783.8 | 1880.81 | 1937.99 | 1654.24 | 1715.02 |
| 544.64 | 488.39 | 468.72 | 446.71 | 454.75 |
| 149.03 | 178.24 | 188.03 | 62.8 | 238.25 |
| 37.29 | 60.94 | 70.76 | 83.92 | 166.39 |
| 38.3 | 54.51 | 46.55 | 49.98 | 91.71 |
| 981.51 | 1095.01 | 1103.48 | 1061.41 | 1006.89 |
| 1.01 | 1 | 1 | 1 | 1 |
| 1.01 | 1 | 1 | 1 | 1 |
| 344.32 | 357.42 | 316.65 | 336.59 | 393.3 |
| 124.87 | 132.44 | 113.88 | 141.24 | 152.21 |
| 120.84 | 117.18 | 134.31 | 92.97 | 180.58 |
| 29.24 | 38.44 | 20.07 | 28.11 | 84.14 |
| 1.01 | 1 | 1 | 1 | 1 |
| 195.34 | 205.56 | 163.82 | 220.44 | 350.75 |
| 103.73 | 89.06 | 71.52 | 86.18 | 180.58 |
| 1.01 | 1 | 1 | 1 | 3.78 |
| 1.01 | 1 | 1 | 1 | 1 |
| 1.01 | 1 | 1 | 1 | 1 |
| 52.39 | 115.57 | 82.11 | 56.01 | 154.1 |
| 1.01 | 1 | 1 | 1 | 1 |
| 33.27 | 36.83 | 19.31 | 42.44 | 59.56 |
| 1052.98 | 1307.93 | 1251.02 | 1222.82 | 960.56 |
| 1.01 | 1 | 1 | 1 | 1 |
| 1.01 | 1 | 1 | 1 | 8.51 |
| 1042.92 | 1076.53 | 1145.1 | 1050.1 | 1077.79 |
| 1330.81 | 1193.04 | 1149.64 | 1124.77 | 1202.59 |
| 42.33 | 27.99 | 20.83 | 13.78 | 14.18 |
| 7.1 | 19.15 | 4.18 | 8.5 | 28.36 |
| 1.01 | 1 | 1 | 1 | 1.89 |
| 1162.71 | 1242.85 | 1288.09 | 1195.66 | 989.87 |
| 375.52 | 525.35 | 526.22 | 602.83 | 654.24 |
| 260.77 | 316.44 | 331.78 | 261.16 | 367.77 |
| 1123.45 | 1081.35 | 981.68 | 1096.1 | 1272.55 |
| 1.01 | 1 | 1 | 1 | 1 |
| 27.23 | 37.63 | 33.69 | 33.39 | 34.98 |
| 762.07 | 702.91 | 704.01 | 697.11 | 795.11 |
| 166.14 | 216.81 | 211.48 | 211.39 | 277.01 |
| 64.47 | 45.67 | 23.1 | 34.89 | 78.47 |

| 10.12 | 13.53 | 12.5 | 23.58 | 16.07 |
| --- | --- | --- | --- | --- |
| 119.84 | 116.38 | 77.57 | 69.59 | 106.83 |
| 36.29 | 81.02 | 91.19 | 79.39 | 107.78 |
| 157.08 | 217.61 | 190.3 | 204.6 | 276.07 |
| 508.4 | 484.37 | 516.38 | 508.55 | 507.7 |
| 4.08 | 1 | 1 | 1 | 4.73 |
| 6165.66 | 6460.64 | 6006.85 | 4925.36 | 6150.06 |
| 66.49 | 82.63 | 70 | 102.78 | 79.41 |
| 3266.56 | 3391.35 | 3099.33 | 3203.44 | 2883.58 |
| 150.04 | 184.67 | 195.59 | 204.6 | 287.41 |
| 186.28 | 208.78 | 205.43 | 166.13 | 168.29 |
| 334.25 | 319.66 | 287.14 | 320 | 419.77 |
| 83.6 | 132.44 | 91.94 | 120.88 | 252.43 |
| 602.01 | 567.13 | 530.76 | 577.94 | 584.28 |
| 311.1 | 403.22 | 354.47 | 373.55 | 545.51 |
| 7704.79 | 7405.53 | 7341.45 | 8023.77 | 5715.16 |
| 1.01 | 1 | 1 | 1 | 1 |
| 19.18 | 39.24 | 20.07 | 33.39 | 52.94 |
| 1.01 | 11.12 | 6.45 | 6.99 | 32.14 |
| 1.01 | 15.14 | 10.23 | 22.83 | 31.2 |
| 156.08 | 157.35 | 147.17 | 153.31 | 144.65 |
| 47.36 | 46.47 | 36.71 | 45.45 | 76.58 |
| 2.06 | 1 | 1 | 1.71 | 1 |
| 1.01 | 1 | 1 | 1 | 9.45 |
| 14.14 | 22.37 | 24.61 | 16.79 | 33.09 |
| 1.01 | 1 | 1 | 1 | 1 |
| 1.01 | 1 | 1 | 1 | 1 |
| 75.55 | 58.52 | 57.9 | 50.73 | 72.8 |
| 920.11 | 720.59 | 658.62 | 910.56 | 676.93 |
| 205.4 | 199.94 | 196.35 | 221.94 | 190.98 |
| 8.1 | 7.91 | 1 | 3.22 | 11.34 |
| 1.01 | 1 | 1 | 1 | 1 |
| 10.12 | 19.96 | 17.8 | 19.81 | 43.49 |
| 2.06 | 1 | 1 | 1 | 1 |
| 14.14 | 30.4 | 20.83 | 48.47 | 63.34 |
| 25.21 | 34.42 | 17.8 | 28.86 | 12.29 |
| 25.21 | 11.92 | 9.48 | 12.27 | 7.56 |
| 1.01 | 1 | 1 | 1 | 1 |
| 57.43 | 72.18 | 61.68 | 71.85 | 99.27 |
| 1.01 | 1 | 1 | 1 | 1 |
| 186.28 | 212.79 | 186.52 | 182.72 | 233.52 |
| 6.09 | 13.53 | 10.99 | 23.58 | 22.69 |
| 1.01 | 1 | 1 | 1 | 1 |
| 1.01 | 1 | 1 | 1 | 1 |
| 1.01 | 1 | 1 | 1 | 1 |
| 1.01 | 1 | 1 | 1 | 1 |
| 3348.1 | 3356.8 | 3377.75 | 3054.1 | 2976.23 |
| 9.11 | 5.5 | 7.21 | 3.22 | 21.74 |

| 40.31 | 29.6 | 28.39 | 35.65 | 43.49 |
| --- | --- | --- | --- | --- |
| 631.21 | 653.9 | 539.84 | 586.24 | 519.99 |
| 42.33 | 49.69 | 42.01 | 31.88 | 61.45 |
| 63.47 | 90.66 | 63.95 | 65.82 | 68.07 |
| 485.24 | 414.47 | 381.71 | 431.62 | 434.9 |
| 1.01 | 1 | 1 | 1 | 1 |
| 150.04 | 201.54 | 233.42 | 226.47 | 275.12 |
| 173.19 | 191.9 | 180.46 | 197.05 | 156.94 |
| 5.08 | 1 | 1 | 1 | 1 |
| 68.5 | 78.61 | 88.16 | 74.11 | 59.56 |
| 801.33 | 1185 | 1130.72 | 1232.62 | 1696.11 |
| 2523.67 | 2438.43 | 2337.46 | 2364.73 | 2315.37 |
| 418.81 | 461.87 | 418.78 | 435.39 | 506.75 |
| 373.51 | 441.78 | 360.53 | 341.87 | 488.79 |
| 2745.13 | 2420.75 | 2310.98 | 2365.49 | 2641.54 |
| 1.01 | 1 | 1 | 1 | 1 |
| 1.01 | 1 | 1 | 1 | 1 |
| 8.1 | 19.15 | 5.69 | 14.53 | 17.96 |
| 139.97 | 146.1 | 141.88 | 158.59 | 126.69 |
| 1624.75 | 1648.61 | 1506.74 | 1693.46 | 1496.62 |
| 505.38 | 565.52 | 580.69 | 539.48 | 465.15 |
| 2204.57 | 1998.12 | 1900.16 | 2000.43 | 2200.03 |
| 941.25 | 877.27 | 880.29 | 984.48 | 1007.83 |
| 89126.03 | 87554.92 | 88154.25 | 89488.88 | 78798.27 |
| 173.19 | 173.42 | 178.19 | 181.22 | 275.12 |
| 1863.32 | 2034.28 | 1995.49 | 2076.61 | 1706.51 |
| 10348.2 | 10379.21 | 10191.46 | 10016.46 | 9069.56 |
| 155.07 | 158.16 | 147.93 | 142 | 163.56 |
| 1428.46 | 1493.54 | 1508.25 | 1334.44 | 1642.22 |
| 31157.25 | 28132.06 | 27668.35 | 28505.82 | 28017.98 |
| 10682.4 | 11000.29 | 11095.57 | 11513.62 | 11184.5 |
| 4409.09 | 4417.4 | 4080.61 | 4231.47 | 4092.79 |
| 587.92 | 478.74 | 495.95 | 482.16 | 488.79 |
| 536.58 | 651.49 | 744.11 | 703.9 | 549.3 |

| 2Aged Con 48h | 3Aged Con 48h | 1Aged Repop 48h | 2Aged Repop 48h | 3Aged Repop 48h |
| --- | --- | --- | --- | --- |
| 359.47 | 337.43 | 333.57 | 351.61 | 318.66 |
| 1175.09 | 1066.56 | 1088.72 | 968.53 | 533.4 |
| 471.26 | 437.67 | 487.78 | 482.99 | 484.1 |
| 162.4 | 135.89 | 158.61 | 154.94 | 174.11 |
| 443.93 | 393.36 | 393.04 | 413.08 | 374.64 |
| 111.89 | 130.62 | 133.02 | 126.51 | 120.63 |
| 1 | 1.06 | 1 | 1 | 1 |
| 1 | 1.06 | 3.7 | 1 | 54.63 |
| 35323.26 | 37425.93 | 34716.64 | 35584.07 | 36377.91 |
| 376.86 | 426.07 | 368.14 | 400.78 | 408.9 |
| 1403.63 | 1375.73 | 1356.35 | 1366.49 | 1617.12 |
| 137.56 | 117.95 | 110.2 | 112.69 | 127.32 |
| 7778.67 | 7128.57 | 8222.59 | 6791.18 | 7445.95 |
| 1 | 1.06 | 3.7 | 1 | 12.01 |
| 15.84 | 17.71 | 23.07 | 25.87 | 22.87 |
| 419.92 | 390.19 | 357.08 | 378.5 | 400.55 |
| 1856.56 | 1615.25 | 1753.98 | 1656.89 | 1328.01 |
| 198.84 | 239.3 | 195.95 | 224.85 | 168.26 |
| 1 | 1.06 | 1.63 | 2.06 | 9.51 |
| 387.63 | 303.67 | 390.96 | 400.01 | 571.84 |
| 198.01 | 237.19 | 191.8 | 211.02 | 257.67 |
| 141.7 | 145.39 | 139.25 | 151.1 | 220.9 |
| 2.59 | 1.06 | 4.4 | 7.43 | 14.52 |
| 192.21 | 227.69 | 218.08 | 157.24 | 123.14 |
| 29.09 | 20.88 | 33.44 | 30.48 | 23.71 |
| 24.12 | 10.33 | 8.55 | 19.73 | 12.01 |
| 1 | 1.06 | 1 | 1 | 1 |
| 1 | 1.06 | 1 | 1 | 1 |
| 1345.66 | 1345.13 | 1785.1 | 1294.27 | 1592.88 |
| 2213.44 | 2102.75 | 2428.22 | 1871.23 | 2192.81 |
| 1882.23 | 1794.63 | 1693.12 | 1809.77 | 2083.36 |
| 63.04 | 47.26 | 61.79 | 58.91 | 117.29 |
| 64.69 | 63.08 | 37.59 | 36.63 | 67.99 |
| 39.02 | 38.82 | 59.72 | 74.27 | 47.94 |
| 71.32 | 49.37 | 65.94 | 46.61 | 53.79 |
| 1390.38 | 1432.71 | 1581.09 | 1419.5 | 2049.93 |
| 1 | 1.06 | 1 | 1 | 1 |
| 1 | 1.06 | 1 | 1 | 1 |
| 3.42 | 1.88 | 9.93 | 4.36 | 7.83 |
| 1 | 1.06 | 1 | 1 | 1 |
| 1 | 1.06 | 1 | 1 | 7.83 |
| 6.73 | 9.27 | 14.08 | 3.59 | 18.7 |
| 1 | 1.06 | 1 | 1 | 1 |
| 1 | 1.06 | 1 | 35.86 | 1 |
| 45.65 | 38.82 | 45.89 | 40.47 | 50.45 |
| 108.58 | 107.4 | 90.84 | 105.77 | 90.55 |
| 222.02 | 154.89 | 200.1 | 227.16 | 272.71 |

| 273.36 | 297.33 | 270.64 | 299.37 | 301.95 |
| --- | --- | --- | --- | --- |
| 84.57 | 62.03 | 65.25 | 70.43 | 90.55 |
| 9.22 | 15.6 | 23.07 | 18.96 | 32.07 |
| 8.39 | 2.94 | 14.08 | 6.67 | 1 |
| 12.53 | 29.32 | 35.52 | 9.74 | 27.89 |
| 49.79 | 16.66 | 38.28 | 37.4 | 107.27 |
| 25.78 | 25.1 | 14.08 | 21.26 | 26.22 |
| 1 | 1.06 | 1 | 1 | 1 |
| 5.08 | 1.06 | 2.32 | 5.13 | 2.82 |
| 1 | 1.06 | 1 | 1 | 1 |
| 153.29 | 161.22 | 148.24 | 131.89 | 224.24 |
| 1 | 1.06 | 1 | 1 | 1 |
| 36.54 | 18.77 | 15.46 | 23.57 | 4.49 |
| 1 | 1.06 | 3.01 | 7.43 | 1 |
| 18.32 | 13.49 | 12 | 20.49 | 23.71 |
| 37.37 | 3.99 | 28.6 | 17.42 | 44.6 |
| 195.52 | 146.44 | 173.82 | 182.6 | 192.49 |
| 19.15 | 5.05 | 14.08 | 12.81 | 12.85 |
| 64.69 | 54.64 | 59.03 | 41.24 | 78.86 |
| 1 | 2.94 | 4.4 | 1 | 1.15 |
| 1.76 | 2.94 | 3.01 | 7.43 | 1 |
| 1 | 1.06 | 1 | 1 | 1 |
| 1 | 1.06 | 1 | 1 | 1 |
| 40.68 | 46.2 | 61.1 | 38.93 | 23.71 |
| 1 | 1.06 | 1 | 1 | 1 |
| 1 | 1.06 | 1 | 1 | 1 |
| 1 | 1.06 | 1 | 1 | 1 |
| 1 | 1.06 | 1 | 4.36 | 1 |
| 2.59 | 9.27 | 1 | 5.13 | 2.82 |
| 19.15 | 27.21 | 25.14 | 22.03 | 21.2 |
| 69.66 | 52.53 | 60.41 | 62.75 | 101.42 |
| 62.21 | 58.86 | 74.24 | 45.85 | 79.69 |
| 1 | 1.06 | 1 | 1 | 1 |
| 33.23 | 39.87 | 29.98 | 29.71 | 66.32 |
| 1920.32 | 1814.68 | 1682.06 | 1778.28 | 1729.08 |
| 1 | 1.06 | 1 | 1 | 1 |
| 53.93 | 57.81 | 43.12 | 52.76 | 52.95 |
| 1 | 1.06 | 1.63 | 2.06 | 1 |
| 1 | 1.06 | 1 | 1 | 1 |
| 7.56 | 14.55 | 3.7 | 8.97 | 6.16 |
| 1 | 1.88 | 1 | 5.9 | 1 |
| 1 | 1.06 | 8.55 | 1 | 43.76 |
| 1 | 1.88 | 1 | 3.59 | 1 |
| 185.59 | 183.38 | 237.44 | 217.94 | 153.22 |
| 1 | 1.06 | 1 | 1 | 1 |
| 58.9 | 79.97 | 75.62 | 61.98 | 65.49 |
| 1 | 1.06 | 1 | 1.29 | 1 |
| 1996.5 | 1899.1 | 1957.98 | 1803.63 | 2044.08 |

| 20.81 | 32.48 | 20.99 | 15.88 | 12.01 |
| --- | --- | --- | --- | --- |
| 4.25 | 5.05 | 4.4 | 7.43 | 3.66 |
| 1 | 1.06 | 1 | 1 | 1 |
| 80.43 | 114.79 | 101.21 | 91.94 | 98.91 |
| 117.69 | 100.02 | 106.74 | 49.69 | 111.44 |
| 1 | 5.05 | 1.63 | 7.43 | 1 |
| 1 | 1.06 | 1 | 1 | 1 |
| 212.91 | 166.49 | 174.51 | 187.21 | 150.71 |
| 1 | 1.06 | 1 | 1 | 1 |
| 60.55 | 46.2 | 62.49 | 58.91 | 38.75 |
| 5.08 | 3.99 | 4.4 | 5.13 | 11.18 |
| 7.56 | 1.06 | 16.84 | 13.58 | 14.52 |
| 97.82 | 102.13 | 63.87 | 95.78 | 81.36 |
| 15.01 | 9.27 | 17.54 | 15.88 | 8.67 |
| 15.01 | 12.44 | 12 | 21.26 | 6.16 |
| 1 | 1.06 | 1 | 1 | 1 |
| 4.25 | 1.06 | 14.77 | 12.04 | 37.08 |
| 392.6 | 423.96 | 212.55 | 302.45 | 189.15 |
| 1 | 1.06 | 1 | 1 | 1 |
| 1.76 | 1.06 | 1 | 4.36 | 1 |
| 13.36 | 7.16 | 12.69 | 8.2 | 1 |
| 14446.83 | 14163.45 | 14153.18 | 13900.67 | 13704.27 |
| 441.45 | 419.74 | 424.85 | 370.82 | 281.9 |
| 959.8 | 757.39 | 828.7 | 826.4 | 739.78 |
| 43.99 | 46.2 | 62.49 | 48.92 | 43.76 |
| 1 | 1.06 | 1 | 1 | 1 |
| 1 | 1.06 | 1 | 1 | 1 |
| 1620.57 | 1554.05 | 1390.23 | 1397.99 | 1363.94 |
| 1 | 1.06 | 1 | 1 | 1 |
| 141.7 | 159.11 | 144.78 | 162.62 | 108.1 |
| 188.9 | 160.16 | 192.49 | 219.47 | 177.45 |
| 139.22 | 134.84 | 146.85 | 118.06 | 223.41 |
| 279.98 | 305.78 | 382.67 | 298.6 | 2603.07 |
| 15.01 | 13.49 | 14.08 | 13.58 | 34.57 |
| 135.08 | 135.89 | 152.38 | 168.77 | 265.19 |
| 1 | 1.06 | 1 | 1 | 1 |
| 1 | 1.06 | 1 | 1 | 1 |
| 592.15 | 595.95 | 609.49 | 536 | 765.68 |
| 1 | 1.06 | 1 | 1 | 1 |
| 182.27 | 174.93 | 162.07 | 189.51 | 141.52 |
| 881.14 | 825.98 | 859.82 | 874.03 | 772.37 |
| 1 | 1.06 | 1 | 1 | 1 |
| 19.15 | 17.71 | 24.45 | 27.41 | 37.91 |
| 1 | 1.06 | 1 | 1 | 1 |
| 1 | 1.06 | 1 | 1 | 1 |
| 53.93 | 51.48 | 48.65 | 49.69 | 34.57 |
| 24654.85 | 25314.58 | 25405.82 | 24513.43 | 29518 |
| 66.35 | 60.97 | 92.22 | 50.46 | 63.82 |

| 1 | 1.06 | 1 | 1 | 1 |
| --- | --- | --- | --- | --- |
| 187.24 | 181.26 | 211.86 | 176.45 | 207.53 |
| 794.19 | 760.56 | 776.84 | 734.98 | 833.36 |
| 666.68 | 604.39 | 636.46 | 544.45 | 636.17 |
| 1056.68 | 1079.22 | 1250.54 | 1067.63 | 878.48 |
| 1 | 1.06 | 1 | 1 | 1 |
| 3.42 | 3.99 | 12 | 12.81 | 40.42 |
| 55.59 | 47.26 | 80.46 | 66.59 | 36.24 |
| 1 | 1.06 | 1 | 1 | 1 |
| 7468.98 | 7759.56 | 6914.2 | 6831.9 | 6863.57 |
| 230.3 | 193.93 | 187.65 | 194.89 | 188.31 |
| 1 | 1.06 | 1 | 1 | 1 |
| 3592.12 | 3651.75 | 4151.53 | 3423.89 | 4503.96 |
| 419.92 | 485.16 | 395.8 | 394.64 | 449.01 |
| 2259.81 | 2421.41 | 2459.34 | 2285.33 | 1820.99 |
| 766.04 | 783.77 | 684.87 | 602.84 | 364.62 |
| 1 | 1.06 | 1 | 1 | 5.33 |
| 15.01 | 22.99 | 19.61 | 10.51 | 6.16 |
| 1 | 1.06 | 1 | 1 | 1 |
| 1656.18 | 1441.15 | 1245.7 | 1384.93 | 820 |
| 33.23 | 25.1 | 29.98 | 3.59 | 54.63 |
| 1 | 1.06 | 1 | 1 | 1 |
| 1 | 1.06 | 1 | 1 | 1 |
| 2.59 | 11.38 | 7.85 | 5.9 | 7 |
| 1 | 1.06 | 1 | 1 | 1 |
| 1 | 1.06 | 1 | 1.29 | 1.15 |
| 1 | 1.06 | 1 | 1 | 1 |
| 60.55 | 44.09 | 50.04 | 49.69 | 39.59 |
| 1 | 1.06 | 1 | 1 | 1 |
| 1 | 1.06 | 1 | 1 | 1 |
| 79.6 | 93.68 | 47.27 | 82.72 | 50.45 |
| 202.15 | 198.15 | 226.38 | 170.3 | 222.57 |
| 32.4 | 34.59 | 74.24 | 42.77 | 45.43 |
| 1 | 1.06 | 1 | 1 | 1 |
| 34.88 | 20.88 | 24.45 | 28.18 | 32.07 |
| 46.48 | 34.59 | 38.97 | 42.01 | 30.39 |
| 273.36 | 273.07 | 223.61 | 210.25 | 92.23 |
| 216.22 | 242.47 | 206.32 | 202.57 | 375.48 |
| 48.96 | 34.59 | 38.97 | 24.34 | 23.71 |
| 395.91 | 402.85 | 342.56 | 374.66 | 329.52 |
| 50.62 | 57.81 | 45.89 | 42.01 | 37.91 |
| 25.78 | 6.11 | 16.15 | 21.26 | 39.59 |
| 2.59 | 1.06 | 1 | 1 | 1 |
| 1 | 1.06 | 1 | 5.13 | 1 |
| 1 | 1.06 | 1 | 1 | 1 |
| 125.97 | 103.18 | 135.79 | 123.44 | 123.14 |
| 110.24 | 84.19 | 126.11 | 108.84 | 129.83 |
| 304 | 334.27 | 420.01 | 292.46 | 771.53 |

| 188.9 | 167.55 | 169.67 | 164.93 | 437.31 |
| --- | --- | --- | --- | --- |
| 29.09 | 18.77 | 56.95 | 34.32 | 113.95 |
| 388.46 | 357.48 | 295.53 | 305.52 | 220.07 |
| 224.5 | 215.03 | 290.69 | 254.05 | 957.86 |
| 749.48 | 693.03 | 708.38 | 720.38 | 352.92 |
| 1 | 1.06 | 1 | 1 | 1 |
| 1 | 1.06 | 1 | 1 | 1 |
| 636.04 | 640.27 | 553.47 | 581.32 | 705.52 |
| 3306.45 | 3155.82 | 3089.33 | 3174.21 | 3262.33 |
| 43.99 | 30.37 | 57.64 | 59.68 | 20.37 |
| 1 | 1.06 | 1 | 1 | 1 |
| 21.64 | 13.49 | 20.3 | 10.51 | 22.04 |
| 90.36 | 75.75 | 74.24 | 77.35 | 78.86 |
| 189.73 | 199.2 | 211.16 | 189.51 | 204.19 |
| 8.39 | 10.33 | 10.62 | 12.81 | 1 |
| 1 | 1.06 | 1 | 1 | 1 |
| 1 | 1.06 | 1 | 1 | 1 |
| 1 | 1.06 | 1 | 1 | 1 |
| 189.73 | 199.2 | 101.9 | 152.63 | 50.45 |
| 89.53 | 109.51 | 38.28 | 71.2 | 18.7 |
| 246.86 | 239.3 | 253.35 | 238.68 | 205.03 |
| 11.7 | 20.88 | 14.08 | 14.35 | 11.18 |
| 1 | 1.06 | 1 | 1 | 1 |
| 55.59 | 63.08 | 23.07 | 35.09 | 12.85 |
| 560.69 | 524.2 | 592.89 | 392.33 | 516.69 |
| 7.56 | 6.11 | 15.46 | 6.67 | 1 |
| 1 | 1.06 | 1 | 1 | 1 |
| 1 | 1.06 | 1 | 1 | 1 |
| 1 | 1.06 | 1 | 1 | 1 |
| 1 | 1.06 | 1 | 1 | 1 |
| 1 | 1.06 | 1 | 1 | 1.15 |
| 231.96 | 235.08 | 251.97 | 237.14 | 290.25 |
| 1106.36 | 977.93 | 1103.94 | 1082.23 | 1185.13 |
| 11.7 | 8.22 | 16.15 | 7.43 | 7.83 |
| 46.48 | 35.65 | 55.57 | 48.92 | 113.95 |
| 133.42 | 93.68 | 128.87 | 101.93 | 81.36 |
| 16.67 | 17.71 | 21.68 | 22.8 | 32.07 |
| 525.91 | 734.18 | 654.44 | 627.42 | 932.8 |
| 1 | 1.06 | 1 | 1 | 1 |
| 203.8 | 170.71 | 188.34 | 181.06 | 470.73 |
| 5.9 | 15.6 | 9.93 | 8.97 | 8.67 |
| 118.52 | 106.35 | 91.53 | 67.36 | 126.48 |
| 193.04 | 189.71 | 204.25 | 197.19 | 227.59 |
| 330.49 | 296.28 | 305.91 | 293.23 | 332.87 |
| 182.27 | 166.49 | 192.49 | 198.73 | 287.75 |
| 1 | 1.06 | 1 | 1 | 1 |
| 1 | 1.06 | 1 | 1 | 1 |
| 1574.2 | 1471.75 | 1362.57 | 1473.28 | 1457.52 |

| 462.98 | 409.18 | 406.18 | 366.98 | 403.89 |
| --- | --- | --- | --- | --- |
| 1 | 1.06 | 1 | 1 | 1 |
| 1 | 1.06 | 1 | 1 | 1 |
| 535.85 | 498.87 | 528.58 | 510.64 | 619.46 |
| 851.33 | 828.09 | 819.02 | 913.98 | 751.48 |
| 348.71 | 346.93 | 313.51 | 327.8 | 345.4 |
| 604.57 | 623.39 | 606.72 | 615.13 | 596.07 |
| 337.12 | 346.93 | 310.05 | 347 | 313.65 |
| 20.81 | 13.49 | 23.76 | 15.12 | 40.42 |
| 364.44 | 365.92 | 327.34 | 361.6 | 404.72 |
| 12.53 | 18.77 | 10.62 | 19.73 | 1.15 |
| 153.29 | 110.57 | 155.84 | 128.82 | 144.87 |
| 1 | 1.06 | 1 | 1 | 1 |
| 271.7 | 211.87 | 249.2 | 254.81 | 210.04 |
| 1 | 1.06 | 1 | 1 | 1 |
| 128.45 | 107.4 | 133.71 | 93.48 | 77.19 |
| 349.54 | 314.22 | 335.64 | 291.69 | 203.35 |
| 22.46 | 10.33 | 20.99 | 16.65 | 15.35 |
| 26.6 | 32.48 | 28.6 | 30.48 | 32.07 |
| 1 | 1.06 | 1 | 1 | 1 |
| 1 | 1.06 | 1 | 1 | 12.01 |
| 1 | 1.06 | 1 | 1 | 1 |
| 1 | 1.88 | 3.01 | 1 | 1 |
| 201.32 | 203.42 | 204.94 | 180.29 | 200.01 |
| 1.76 | 1.06 | 5.78 | 9.74 | 1 |
| 16.67 | 26.15 | 29.98 | 21.26 | 15.35 |
| 361.13 | 221.36 | 179.35 | 262.5 | 408.9 |
| 1 | 1.06 | 1 | 1 | 1 |
| 1 | 1.06 | 1 | 6.67 | 1 |
| 1.76 | 1.06 | 5.09 | 1 | 1 |
| 260.94 | 276.23 | 233.99 | 267.87 | 324.51 |
| 20.81 | 27.21 | 36.21 | 39.7 | 17.03 |
| 4.25 | 6.11 | 3.01 | 2.82 | 1 |
| 1127.06 | 1063.39 | 1139.2 | 1132.94 | 1241.95 |
| 1 | 1.06 | 1 | 1 | 1 |
| 1 | 1.06 | 1 | 1 | 1 |
| 1 | 1.06 | 1 | 1 | 1 |
| 16.67 | 20.88 | 14.08 | 13.58 | 8.67 |
| 8.39 | 2.94 | 3.01 | 13.58 | 1 |
| 163.23 | 120.06 | 166.91 | 124.98 | 177.45 |
| 10.04 | 3.99 | 5.78 | 8.2 | 100.58 |
| 769.35 | 761.61 | 767.85 | 762.63 | 774.88 |
| 5.08 | 1.06 | 1 | 1 | 1.15 |
| 3.42 | 2.94 | 4.4 | 6.67 | 3.66 |
| 1 | 1.06 | 1 | 1 | 1 |
| 1 | 1.06 | 1 | 1 | 1 |
| 1 | 1.06 | 1 | 1 | 1 |
| 5.08 | 1.06 | 1 | 1 | 1 |

| 1 | 10.33 | 9.24 | 12.04 | 2.82 |
| --- | --- | --- | --- | --- |
| 300.68 | 168.6 | 117.81 | 140.34 | 274.38 |
| 1 | 1.06 | 2.32 | 1 | 1 |
| 5.08 | 1.06 | 3.01 | 3.59 | 1 |
| 1 | 1.06 | 1 | 1 | 1 |
| 1 | 1.06 | 2.32 | 1 | 1 |
| 1 | 1.06 | 1 | 1 | 1 |
| 1 | 1.06 | 1 | 4.36 | 1 |
| 1 | 1.06 | 1 | 1 | 1 |
| 1 | 1.06 | 1 | 1 | 1 |
| 7.56 | 1.06 | 5.78 | 11.27 | 4.49 |
| 12.53 | 13.49 | 22.38 | 18.19 | 2.82 |
| 1 | 1.06 | 1 | 1 | 1 |
| 1451.65 | 1469.64 | 1277.51 | 1421.8 | 557.63 |
| 1 | 1.06 | 1 | 1 | 1 |
| 183.1 | 145.39 | 190.42 | 187.21 | 462.38 |
| 1 | 1.06 | 1 | 1 | 1 |
| 1 | 1.06 | 1 | 1 | 1 |
| 130.94 | 132.73 | 146.16 | 95.78 | 165.75 |
| 907.63 | 813.32 | 724.97 | 745.73 | 704.69 |
| 1 | 1.88 | 1.63 | 1 | 1 |
| 8.39 | 3.99 | 18.92 | 8.2 | 1.15 |
| 1 | 1.06 | 1 | 1 | 1 |
| 605.4 | 532.64 | 658.59 | 594.38 | 640.35 |
| 594.64 | 579.07 | 665.5 | 597.46 | 616.12 |
| 465.46 | 417.63 | 454.58 | 451.49 | 484.1 |
| 58.9 | 59.92 | 50.04 | 59.68 | 54.63 |
| 104.44 | 84.19 | 104.67 | 87.33 | 93.06 |
| 138.39 | 119.01 | 110.89 | 146.49 | 139.02 |
| 16.67 | 8.22 | 30.67 | 18.96 | 1.15 |
| 1 | 1.88 | 3.01 | 1 | 2.82 |
| 68.01 | 88.41 | 77.7 | 58.14 | 70.5 |
| 43.99 | 22.99 | 70.78 | 41.24 | 42.09 |
| 53.1 | 53.59 | 65.94 | 60.44 | 61.31 |
| 482.85 | 442.95 | 423.47 | 380.81 | 469.06 |
| 1 | 1.06 | 1 | 4.36 | 1 |
| 158.26 | 194.98 | 217.39 | 221.78 | 167.43 |
| 25.78 | 14.55 | 24.45 | 14.35 | 26.22 |
| 679.1 | 589.62 | 720.13 | 648.16 | 616.96 |
| 15.84 | 8.22 | 11.31 | 12.81 | 14.52 |
| 226.16 | 259.35 | 290 | 230.23 | 204.19 |
| 19.98 | 21.93 | 15.46 | 13.58 | 5.33 |
| 1501.33 | 1534 | 1452.47 | 1342.67 | 1643.02 |
| 69.66 | 66.25 | 81.16 | 48.15 | 73.01 |
| 1 | 1.06 | 1 | 1 | 1 |
| 3563.97 | 3476.59 | 3188.22 | 3304.04 | 3496.28 |
| 1045.92 | 1056.01 | 1127.45 | 1063.79 | 1080.69 |
| 66.35 | 54.64 | 92.91 | 71.97 | 68.83 |

| 1 | 1.06 | 1 | 1 | 1 |
| --- | --- | --- | --- | --- |
| 1 | 1.06 | 3.01 | 1 | 1 |
| 389.28 | 310 | 254.73 | 309.36 | 311.14 |
| 1 | 1.06 | 1 | 1 | 1 |
| 1 | 1.06 | 1 | 1 | 1 |
| 1 | 1.06 | 1 | 1 | 1 |
| 1 | 1.06 | 1 | 1 | 1 |
| 1 | 1.06 | 1 | 1 | 1 |
| 1 | 1.06 | 1 | 1 | 1 |
| 1 | 1.06 | 1 | 1 | 1 |
| 1 | 1.06 | 1 | 1 | 1 |
| 1 | 1.06 | 1 | 1 | 1 |
| 1 | 1.06 | 1 | 1 | 1 |
| 1 | 1.06 | 1 | 1 | 1 |
| 1 | 1.06 | 1 | 1 | 1 |
| 1 | 1.06 | 1 | 1 | 1 |
| 130.11 | 121.12 | 115.04 | 141.88 | 108.94 |
| 19.15 | 20.88 | 36.9 | 17.42 | 14.52 |
| 22.46 | 30.37 | 41.05 | 37.4 | 32.9 |
| 145.01 | 171.77 | 191.11 | 171.07 | 116.46 |
| 1 | 2.94 | 9.93 | 15.88 | 1.15 |
| 14.18 | 19.82 | 7.16 | 1 | 9.51 |
| 1 | 1.06 | 1 | 1 | 1 |
| 4.25 | 7.16 | 2.32 | 1 | 1 |
| 58.9 | 53.59 | 53.5 | 34.32 | 90.55 |
| 348.71 | 322.66 | 310.75 | 303.21 | 344.56 |
| 1 | 1.06 | 1 | 1 | 6.16 |
| 12.53 | 12.44 | 11.31 | 15.12 | 8.67 |
| 1.76 | 1.06 | 1 | 1 | 1 |
| 1 | 1.06 | 1 | 1 | 1 |
| 114.38 | 91.57 | 85.31 | 101.16 | 122.31 |
| 1 | 1.06 | 1 | 3.59 | 1 |
| 528.39 | 535.81 | 657.9 | 435.35 | 381.33 |
| 60.55 | 71.53 | 56.26 | 72.74 | 91.39 |
| 212.08 | 163.33 | 186.96 | 218.71 | 158.23 |
| 1 | 2.94 | 3.7 | 1 | 4.49 |
| 584.7 | 587.51 | 541.72 | 544.45 | 632.83 |
| 1044.26 | 1013.8 | 995.36 | 1073.01 | 1123.3 |
| 7849.88 | 8162.64 | 7311.15 | 7684.67 | 8899.82 |
| 102.78 | 141.17 | 105.36 | 99.62 | 88.88 |
| 925.02 | 886.12 | 908.23 | 951.63 | 932.8 |
| 395.08 | 400.74 | 393.73 | 405.39 | 438.98 |
| 1 | 1.06 | 1 | 1 | 1 |
| 112.72 | 88.41 | 99.83 | 102.7 | 87.21 |
| 1 | 1.06 | 3.01 | 1 | 1 |
| 1 | 1.06 | 1 | 1 | 1 |
| 9707.99 | 8924.48 | 9494.32 | 9576.9 | 8365.9 |
| 5703.61 | 5964.7 | 5677.74 | 5835.46 | 5453.99 |

| 248.52 | 218.2 | 260.26 | 194.12 | 289.42 |
| --- | --- | --- | --- | --- |
| 173.99 | 182.32 | 217.39 | 170.3 | 189.99 |
| 1.76 | 1.06 | 1 | 1 | 1 |
| 8.39 | 3.99 | 4.4 | 1 | 24.55 |
| 3.42 | 14.55 | 2.32 | 9.74 | 34.57 |
| 209.6 | 206.59 | 255.42 | 244.83 | 228.42 |
| 77.94 | 105.29 | 80.46 | 81.95 | 108.94 |
| 6096.1 | 5775.83 | 6240.65 | 6117.41 | 5631.96 |
| 28.26 | 29.32 | 27.91 | 29.71 | 23.71 |
| 21.64 | 31.43 | 29.29 | 33.55 | 30.39 |
| 258.45 | 293.11 | 240.21 | 214.1 | 208.37 |
| 279.15 | 296.28 | 267.18 | 290.92 | 252.65 |
| 290.75 | 262.51 | 257.5 | 270.95 | 246.8 |
| 140.87 | 140.11 | 139.25 | 137.27 | 144.87 |
| 68.01 | 55.7 | 55.57 | 94.25 | 82.2 |
| 270.05 | 319.49 | 370.91 | 300.91 | 829.19 |
| 143.36 | 112.68 | 151 | 137.27 | 144.87 |
| 1.76 | 8.22 | 5.78 | 1 | 4.49 |
| 14.18 | 1.88 | 12 | 12.81 | 3.66 |
| 292.4 | 333.21 | 360.54 | 330.87 | 332.87 |
| 203.8 | 235.08 | 231.91 | 226.39 | 173.27 |
| 1 | 1.06 | 1 | 1 | 1 |
| 1 | 1.06 | 1 | 1 | 1 |
| 15.01 | 1.06 | 3.7 | 16.65 | 10.34 |
| 2059.43 | 1979.29 | 1908.88 | 2024.89 | 1803.44 |
| 121 | 120.06 | 115.04 | 97.32 | 259.34 |
| 4.25 | 1.06 | 8.55 | 10.51 | 1 |
| 5.08 | 1.06 | 3.01 | 1 | 1 |
| 1 | 1.06 | 1 | 1 | 1 |
| 106.92 | 84.19 | 109.51 | 104.23 | 93.9 |
| 515.14 | 551.63 | 482.25 | 537.53 | 375.48 |
| 172.34 | 157 | 186.27 | 199.5 | 216.72 |
| 809.93 | 682.48 | 823.17 | 659.69 | 1034.73 |
| 2463.51 | 2419.3 | 2334.87 | 2505.82 | 1942.98 |
| 900.18 | 922 | 920.68 | 939.33 | 842.56 |
| 1 | 1.06 | 1 | 1 | 1 |
| 1 | 1.06 | 1 | 1 | 1 |
| 1 | 1.06 | 1 | 1 | 1 |
| 55.59 | 57.81 | 36.9 | 59.68 | 21.2 |
| 24.95 | 20.88 | 41.74 | 32.02 | 218.39 |
| 304 | 310 | 274.79 | 292.46 | 321.17 |
| 165.71 | 161.22 | 157.92 | 174.15 | 148.21 |
| 55.59 | 44.09 | 48.65 | 69.66 | 51.28 |
| 50.62 | 28.26 | 29.98 | 99.62 | 1.15 |
| 7.56 | 1.06 | 3.7 | 5.13 | 1 |
| 1 | 1.06 | 1 | 1 | 1 |
| 204.63 | 187.6 | 175.21 | 187.21 | 147.37 |
| 1287.7 | 1349.35 | 1175.85 | 1016.16 | 1496.8 |

| 97.82 | 78.91 | 110.89 | 97.32 | 107.27 |
| --- | --- | --- | --- | --- |
| 1 | 1.06 | 1 | 1 | 1 |
| 2437.84 | 2295.84 | 2312.74 | 2430.53 | 2555.45 |
| 2289.62 | 2195.6 | 2159.22 | 2160.87 | 2545.42 |
| 56.41 | 39.87 | 73.55 | 50.46 | 27.05 |
| 3448.05 | 3300.38 | 3125.98 | 3362.43 | 3514.66 |
| 3246.83 | 3109.39 | 2935.81 | 3041.3 | 3197.99 |
| 46.48 | 38.82 | 32.06 | 36.63 | 13.68 |
| 1 | 1.06 | 1 | 2.06 | 1 |
| 277.5 | 269.9 | 256.81 | 211.79 | 232.6 |
| 1855.73 | 1756.65 | 1821.75 | 1859.71 | 1977.24 |
| 567.31 | 490.43 | 433.15 | 443.04 | 406.39 |
| 266.73 | 165.44 | 142.01 | 199.5 | 256.83 |
| 130.94 | 112.68 | 117.12 | 102.7 | 112.28 |
| 77.94 | 63.08 | 76.32 | 79.65 | 67.16 |
| 1066.62 | 1092.94 | 1076.97 | 1139.85 | 1312.97 |
| 1 | 1.06 | 1 | 3.59 | 1 |
| 1 | 1.06 | 1 | 1 | 1 |
| 373.55 | 341.65 | 333.57 | 357.76 | 441.49 |
| 123.48 | 117.95 | 146.16 | 151.87 | 130.66 |
| 220.36 | 133.78 | 146.85 | 200.27 | 273.54 |
| 55.59 | 66.25 | 55.57 | 57.37 | 69.67 |
| 1 | 1.06 | 1 | 1 | 1 |
| 299.86 | 174.93 | 325.96 | 1247.41 | 708.87 |
| 187.24 | 100.02 | 157.23 | 569.8 | 383.83 |
| 1 | 1.06 | 1 | 1 | 15.35 |
| 1 | 1.06 | 3.7 | 5.9 | 1 |
| 1 | 1.06 | 1 | 1 | 1 |
| 122.66 | 95.8 | 117.81 | 132.66 | 200.01 |
| 1 | 1.06 | 1 | 1 | 1 |
| 37.37 | 35.65 | 43.12 | 39.7 | 38.75 |
| 1123.75 | 1128.82 | 1117.77 | 1103.74 | 1250.31 |
| 1 | 1.06 | 1 | 1 | 1 |
| 1 | 1.06 | 1 | 1 | 1 |
| 1128.72 | 1116.15 | 1115.69 | 1099.13 | 1212.71 |
| 1310.89 | 1238.55 | 1120.53 | 1205.15 | 1131.66 |
| 40.68 | 29.32 | 25.14 | 32.02 | 17.86 |
| 20.81 | 22.99 | 31.37 | 21.26 | 63.82 |
| 1 | 1.06 | 1 | 1 | 1 |
| 994.58 | 1275.49 | 1201.44 | 1150.61 | 1202.68 |
| 597.95 | 572.74 | 579.06 | 457.63 | 420.6 |
| 372.72 | 282.56 | 285.16 | 277.86 | 332.87 |
| 1159.36 | 1213.23 | 1211.81 | 1106.81 | 1683.12 |
| 1 | 1.06 | 1 | 1 | 1 |
| 50.62 | 48.31 | 56.26 | 42.01 | 59.64 |
| 870.37 | 756.34 | 733.27 | 718.84 | 733.93 |
| 207.12 | 242.47 | 254.04 | 264.03 | 286.07 |
| 40.68 | 41.98 | 36.9 | 55.83 | 52.95 |

| 12.53 | 19.82 | 34.82 | 15.88 | 44.6 |
| --- | --- | --- | --- | --- |
| 122.66 | 103.18 | 146.85 | 117.29 | 102.25 |
| 101.96 | 105.29 | 86.69 | 85.03 | 108.1 |
| 210.43 | 171.77 | 217.39 | 191.82 | 224.24 |
| 469.6 | 485.16 | 490.54 | 502.19 | 554.29 |
| 1 | 1.06 | 1 | 1 | 1 |
| 6057.18 | 6241.16 | 5603.75 | 6420.11 | 7188.6 |
| 83.74 | 78.91 | 81.85 | 65.05 | 52.95 |
| 3229.44 | 3228.62 | 3002.89 | 3180.35 | 5005.29 |
| 236.93 | 162.27 | 200.1 | 218.71 | 204.19 |
| 200.49 | 232.97 | 225 | 214.86 | 210.87 |
| 423.23 | 382.8 | 347.4 | 378.5 | 428.12 |
| 162.4 | 155.94 | 170.36 | 127.28 | 204.19 |
| 629.41 | 626.55 | 598.42 | 565.96 | 503.32 |
| 462.98 | 471.44 | 478.79 | 458.4 | 395.53 |
| 6714.64 | 6722.32 | 6603.71 | 7155.34 | 6600.37 |
| 1 | 1.06 | 1 | 1 | 1 |
| 58.9 | 46.2 | 50.04 | 44.31 | 45.43 |
| 31.57 | 7.16 | 18.92 | 14.35 | 20.37 |
| 34.06 | 36.71 | 31.37 | 31.25 | 18.7 |
| 177.31 | 155.94 | 148.93 | 151.1 | 148.21 |
| 46.48 | 47.26 | 59.72 | 47.38 | 76.35 |
| 1.76 | 1.88 | 5.78 | 1 | 1 |
| 5.08 | 11.38 | 5.09 | 2.82 | 1 |
| 24.95 | 29.32 | 38.28 | 29.71 | 11.18 |
| 1.76 | 8.22 | 7.16 | 1.29 | 9.51 |
| 1 | 1.06 | 1 | 1 | 1 |
| 66.35 | 54.64 | 66.63 | 65.05 | 83.87 |
| 785.91 | 752.12 | 881.95 | 737.28 | 577.68 |
| 203.8 | 194.98 | 224.3 | 233.3 | 247.64 |
| 15.01 | 5.05 | 11.31 | 2.82 | 4.49 |
| 1 | 1.06 | 1 | 1 | 1 |
| 37.37 | 18.77 | 31.37 | 26.64 | 16.19 |
| 7.56 | 13.49 | 1 | 1 | 1 |
| 31.57 | 28.26 | 38.28 | 34.32 | 37.91 |
| 28.26 | 19.82 | 15.46 | 25.1 | 51.28 |
| 8.39 | 18.77 | 25.14 | 18.96 | 5.33 |
| 1 | 1.06 | 1 | 1 | 1 |
| 101.13 | 64.14 | 60.41 | 53.53 | 17.86 |
| 1 | 1.06 | 1 | 1 | 1 |
| 213.74 | 232.97 | 231.22 | 186.44 | 170.77 |
| 19.98 | 11.38 | 26.53 | 21.26 | 24.55 |
| 1 | 1.06 | 1 | 1 | 1 |
| 1 | 1.06 | 1 | 1 | 1 |
| 1 | 1.06 | 1 | 1 | 1 |
| 1 | 1.06 | 1 | 1 | 1 |
| 3189.7 | 2868.81 | 2965.55 | 3153.46 | 4236.58 |
| 5.9 | 9.27 | 1 | 8.2 | 1 |

| 53.1 | 27.21 | 49.35 | 33.55 | 24.55 |
| --- | --- | --- | --- | --- |
| 549.09 | 601.23 | 507.14 | 559.04 | 447.34 |
| 58.9 | 60.97 | 41.74 | 53.53 | 36.24 |
| 78.77 | 68.36 | 72.17 | 68.13 | 42.93 |
| 407.5 | 441.89 | 402.72 | 448.42 | 550.11 |
| 1 | 1.06 | 1 | 1 | 1 |
| 257.63 | 254.07 | 272.02 | 247.13 | 123.98 |
| 192.21 | 141.17 | 175.21 | 185.67 | 166.59 |
| 1 | 2.94 | 1 | 2.82 | 1 |
| 71.32 | 73.64 | 85.31 | 81.95 | 68.83 |
| 1518.72 | 1541.39 | 1555.51 | 1334.99 | 1186.8 |
| 2654.79 | 2455.18 | 2327.26 | 2402.87 | 2492.78 |
| 432.34 | 416.57 | 444.9 | 413.08 | 304.46 |
| 480.37 | 415.51 | 411.02 | 425.37 | 301.11 |
| 2456.89 | 2330.67 | 2329.33 | 2506.59 | 2139.34 |
| 1 | 1.06 | 1 | 1 | 1 |
| 1 | 1.06 | 1 | 1 | 1 |
| 10.87 | 26.15 | 13.39 | 24.34 | 22.04 |
| 131.76 | 104.24 | 159.3 | 136.5 | 195 |
| 1757.2 | 1658.52 | 1566.57 | 1543.19 | 1352.24 |
| 489.48 | 526.31 | 491.93 | 534.46 | 647.04 |
| 2317.78 | 2266.3 | 2024.37 | 1984.94 | 1574.5 |
| 999.55 | 937.83 | 975.31 | 957 | 1086.54 |
| 83853.57 | 83372.67 | 83061.79 | 84790.42 | 90074.91 |
| 199.66 | 209.75 | 238.83 | 224.08 | 226.75 |
| 1846.63 | 1802.02 | 1900.58 | 1865.09 | 1809.29 |
| 10131.12 | 10264.56 | 9692.1 | 9623.76 | 9147.98 |
| 145.01 | 184.43 | 144.09 | 143.42 | 118.13 |
| 1699.24 | 1507.63 | 1621.2 | 1637.68 | 2341.54 |
| 29132.04 | 28175.17 | 27174.08 | 28280.21 | 30745.43 |
| 11080.87 | 10757.33 | 11702.39 | 11484.49 | 12658.99 |
| 4469.84 | 4026.34 | 4132.16 | 4139.91 | 4077.83 |
| 492.79 | 467.22 | 536.88 | 480.68 | 413.91 |
| 514.32 | 557.96 | 547.25 | 608.98 | 470.73 |
