# Supplementary material for: Replacement of microglia in the aged brain reverses cognitive, synaptic, and neuronal deficits in mice
Source: Aging Cell. 2018 Oct 2;17(6):e12832. doi: 10.1111/acel.12832 (PMC6260908; doi:10.1111/acel.12832)
Supplement: Supplementary file 4 [file ACEL-17-e12832-s004.docx]

**Replacement of Microglia in the Aged Brain Reverses Age-Induced Cognitive, Synaptic, and Neuronal Deficits in Mice**

**Running Title:** Microglial repopulation rescues aging deficits

Monica R. P. Elmore^1,2,τ^, Lindsay A. Hohsfield^1,2, τ^, Enikö A. Kramár^1^, Lilach Soreq^3,4^, Rafael J. Lee^1,2^, Stephanie T. Pham^1,2^, Allison R. Najafi^1,2^, Elizabeth E. Spangenberg^1,2^, Marcelo A. Wood^1^, Brian L. West^5^, and Kim N. Green^1,2^*

^1^Department of Neurobiology and Behavior, University of California, Irvine, Irvine, CA 92697, USA.

^2^Institute for Memory Impairments and Neurological Disorders (UCI MIND), Irvine, CA 92697, USA.

^3^University College London, London WC1N 3BG, England.

^4^The Francis Crick Institute, 1 Midland Road, London NW1 1AT, England.

^5^ Plexxikon Inc., Berkeley, CA 94710, USA.

^τ^ These authors contributed equally to this study.

*Correspondence to: Kim N. Green, Ph.D.

3208 Biological Sciences III

University of California, Irvine (UCI)

Irvine, CA 92697, USA

Email: kngreen@uci.edu

Keywords: microglia, colony-stimulating factor 1 receptor, plx5622, repopulation, long-term potentiation, aging

**Experimental Procedures:**

**Compounds**

CSF1R inhibitor (PLX5622) were provided by Plexxikon Inc. (Berkeley, CA, USA) and formulated in standard chow by Research Diets Inc. (New Brunswick, NJ, USA) at 1200 ppm chow.

**Experimental Design**

All rodent experiments were performed in accordance with animal protocols approved by the Institutional Animal Care and Use Committee at the University of California, Irvine (UCI). Animals were provided with housing allowing open access to food and water under 12 h/12 h light-dark cycles.

*Mice:* Male C57BL/6 young (~3 months) mice were obtained from The Jackson Laboratory and aged (22 months) mice were obtained from the National Institutes on Aging aged rodent colony. Of note, both aged and young mice derive from the same C57BL/6 strain.

*Treatments:* To eliminate microglia, PLX5622 was administered ad libitum for 7 days (Supplemental Figure 1) or 14 days (all other figures). Animal weights were monitored over the course of the study to ensure adequate animal welfare. To stimulate microglial repopulation, PLX5622 was withdrawn at the indicated time point(s) for each assay, resulting in microglial repopulation ranging from 3 to 28 days. For LTP analyses, recordings began after 28 days repopulation, thus mice were sacrificed 4 to 8 weeks after inhibitor removal. Experimental groups include: young control (denoted as Con in figures), young microglial-eliminated (Elim), young microglial-repopulated (Repop), aged control (Aged Con), aged microglial-eliminated (Aged Elim), and aged microglial-repopulated (Aged Repop).

*LPS:* Lipopolysaccharide (LPS; *Escherichia coli* 0111:B4; Cat. #L4130, Sigma-Aldrich, St. Louis, MO, USA) was dissolved in phosphate-buffered saline (PBS) and administered intraperitoneally at a dose of 0.33 mg/kg body weight 6 or 48 hours prior to sacrifice. Appropriate PBS controls were utilized.

*BrdU labeling:* Bromodeoxyuridine (BrdU; Cat. #000103, Thermo Fisher Scientific, Waltham, MA, USA) injections were administered intraperitoneally to all mice at a dose of 1 ml/100 g body weight (per manufacturer’s instructions) twice daily for 5 consecutive days immediately prior to sacrifice.

Mice were sacrificed via carbon dioxide inhalation and perfused transcardially with 1X PBS. Brains were extracted and dissected down the midline, with one half flash-frozen for subsequent RNA and protein analyses, and the other half drop-fixed in 4% paraformaldehyde. Fixed brains were cryopreserved in 30% sucrose, frozen, and sectioned at 40 μm on a Leica SM2000 R sliding microtome for subsequent immunohistochemical analyses.

**Immunohistochemistry and Microscopy**

Fluorescent immunolabeling followed a standard indirect technique (i.e. primary antibody followed by fluorescent secondary antibody), as previously described ([M. R. P. Elmore et al., 2014](#_ENREF_4)). Primary antibodies include: IBA1 (1:1000; Cat. #019-19741, Wako, Richmond, VA, USA), IB4 (1:200; Cat. #I21414, Thermo Fisher Scientific, Waltham, MA, USA), P2RY12 (1:200, Cat. #HPA014518, Sigma-Aldrich, St. Louis, MO, USA), TMEM119 (1:200, Cat. #ab209064, Abcam), CD68 (1:1000; Cat. #MCA1957GA, Bio-Rad, Hercules, CA, USA), BrdU (1:500; Cat. #ab6326, Abcam), NeuN (1:1000; Cat. #MAB377, EMD Millipore, Billerica, MA, CA), and DCX (1:500; Cat. #sc-8066, Santa Cruz Biotechnology, Dallas, TX, USA). High resolution fluorescent images were obtained using a Leica TCS SPE-II Confocal microscope. To quantify BrdU^+^ cells, counting was performed in the subgranular zone (SGZ) and granule cell layer (GCL) in serial brain sections (every 6^th^, 40 μm thickness) throughout the hippocampus by a blinded observer using a Zeiss Axioskop upright light microscope and corrected for total hippocampal volume (calculated using StereoInvestigator software (Microbrightfield) and the Cavalieri estimator function). Microglial morphology was determined using the filaments module in Bitplane Imaris 7.5, as described previously ([M. R. Elmore, Lee, West, & Green, 2015](#_ENREF_3)). Cell quantities were determined using the spots module in Imaris. Percent coverage measurements were determined in Image J (NIH).

**PK Analysis**

PLX5622 concentration in plasma and cerebellum were analyzed for pharmacokinetic (PK) data by Integrated Analytical Solutions, Inc. (Berkeley, CA, USA).

**Behavioral and Cognitive Tests**

Mouse behavior and cognition was evaluated using the elevated plus maze, the open field arena, accelerating rotarod, and the Morris water maze, in the order listed, and as previously described ([M. R. Elmore et al., 2015](#_ENREF_3)).

**RNA Extraction and Nanostring Analyses**

RNA was extracted and purified from frozen half brains using an RNA Plus Universal Mini Kit (Cat. #73404, Qiagen, Hilden, Germany). For nCounter© analysis, total RNA was diluted to 20 ng/μl and probed using an nCounter© Mouse Immunology panel (Nanostring Technologies, Seattle, WA, USA) profiling 561 immunology-related mouse genes. Counts for target genes were normalized to the best fitting house-keeping genes as determined by nSolver software. Multivariate linear regression analysis was performed using Nanostring nSolver software to determine differentially expressed genes between groups (adj. p <0.05, Benjamini-Yekutieli FDR). Normalized raw expression values are displayed as a heatmap with hierarchical clustering utilizing Morpheus (Broad Institute).

**RNA-Sequencing Library Preparation and Data Analysis**

Gene level expression values from the following groups of control and microglia repopulated mice were analyzed: Con, Repop, Aged, Aged Elim, and Aged Repop. Each group included three mice from which whole transcriptome RNA sequencing libraries were produced. Overall, 23,881 genes were detected as expressed. Casava (version 1.8.2) was used for base calling and ELAND v2 was used for genome alignment to the UCSC mouse genome (version MM10). To determine the association between the population genes (N=23,881) and the various experimental comparisons, we first threshold the data (RPKM > 10, N= 6145) and within this subset we measure the fit of a Generalized Linear regression model (GLM) to the RPKM (Reads Per Kilobase of transcript per Million mapped reads) measurements as a function of both Age (young & aged) and experimental condition (control, repopulated & eliminated) as well as the interaction between the two factors for each gene independently. Following this step, we corrected for multiple comparison across all the coefficient terms using false discovery rate (FDR, with q=0.05). Lastly, we threshold the adjusted p (Alpha=0.05 🡪 N=689, Alpha=0.025 🡪 N=75). All the statistical and classification analyses were performed using target scripts programmed in Matlab (version 2016a). Data were transformed into Z-values, hierarchical clustering performed (one minus pearsons correlations), and heatmaps generated using Morpheus (Broad Institute - <https://software.broadinstitute.org/morpheus/>). Pathway analysis was performed using Ingenuity Pathway Analyses (IPA) software (Qiagen). The RNA sequencing expression dataset is under the Gene Expression Omnibus (GEO) ([Edgar, Domrachev, & Lash, 2002](#_ENREF_2)), accession number GSE94042. <https://www.ncbi.nlm.nih.gov/geo/query/acc.cgi?token=afctauaurxybdyr&acc=GSE94042>

**Dendritic Spine and Sholl Analyses**

Brains were processed and spine data gathered as previously described ([Spangenberg et al., 2016](#_ENREF_8)). In short, five non-primary apical dendrites in the CA1 of each mouse (n=3-5 mice/group) were traced, spine densities quantified, and spine type characterized (mushroom, thin, or stubby ([Peters & Kaiserman-Abramof, 1970](#_ENREF_6))). Traced neurons were analyzed via Sholl analysis using Neurolucida software.

**Long-Term Potentiation**

Hippocampal slices (n=8-9 slices/group) were obtained from Con, Repop, Aged Con, and Aged Repop mice starting 28 days after the CSF1R inhibitor was removed. Hippocampal slices were prepared as previously described ([Barrett et al., 2011; Lopez et al., 2016](#_ENREF_1)) and the electrophysiologist was blinded to treatment. Baseline levels of synaptic transmission were assessed via 1) input/output curves (amplitude of the presynaptic fiber volley to the field excitatory postsynaptic potentials (fEPSPs) slope across a range of stimulating currents) and 2) paired-pulse facilitation ((PPF) of the initial slope of the synaptic response (40, 100, and 200 ms inter-pulse intervals)). fEPSPs were recorded from CA1b stratum radiatum using a single glass pipette (2–3 MΩ). Bipolar stainless-steel stimulation electrodes (25 μm in diameter, FHC) were positioned at two sites (CA1a and CA1c) in the apical Schaffer collateral–commissural projections to provide activation of separate converging pathways of CA1b pyramidal cells. Pulses were administered in an alternating fashion to the two electrodes at 0.03 Hz using a current that elicited a 50% maximal response. After establishing a stable baseline, LTP was induced by delivering five theta bursts (each burst containing four pulses at 100 Hz and bursts were separated by 200 ms). Data were collected and digitized by NAC 2.0 Neurodata Acquisition System (Theta Burst Corp., Irvine, CA, USA).

**Statistical Analysis**

Data were checked for adherence to statistical assumptions (e.g., homogeneity of variance and normality of residuals, etc. ([Grafen & Hails, 2002](#_ENREF_5))) by plotting the residuals within Statistical Analysis Systems (SAS) software (Institute Inc., Cary, NC, USA), and data were transformed as necessary to meet these assumptions. Statistical analysis was performed using a one-way (e.g., treatment) or two-way analysis of variance (ANOVA) (e.g., Age x Diet) with a Tukey post hoc test. For animal behavior acquisition trials (i.e., Rotarod and Morris water maze acquisition trials) a three-way (e.g., Age x Diet x Time) repeated measures ANOVA was performed. A three-way (e.g., Age x Diet x Distance) ANOVA was performed for Sholl analysis. To evaluate biologically relevant interactions between groups for behavioral/cognitive endpoints, post hoc planned pairwise comparisons were employed ([Ruxton & Beauchamp, 2008](#_ENREF_7)). Results from post hoc comparisons are reflected in figures. See “RNA-sequencing library preparation and data analysis” section above for specific methods used to analyze the RNA-Seq data. See “RNA Extraction and NanoString Analyses” for methods used to analyze Nanostring data. The fEPSP slope for LTP was analyzed using a general linear model in SAS. For clarity to the reader, the data are presented as raw means ± standard error of the mean (SEM), regardless of statistical transformation. For analyses, statistical significance was accepted at *p<0.05, **p<0.01, and ***p<0.001 and statistical trends at ^#^p<0.10.

**Supplemental Figure Legends:**

**Supplemental Figure 1: Microglial Elimination and Replacement with PLX5622. A-H)** C57BL/6 mice (~2 mo, n=3 mice/group) were fed control chow or PLX5622 (1200 ppm in chow) for 7 days to eliminate microglia. In three groups, the inhibitor was withdrawn, and the brains investigated 3, 7, and 21 days later. **A)** IBA1^+^ staining (green) in half-brain sections are shown, where each white dot represents a microglial cell. **B)** Representative cortex images with IBA1^+^ (green) and IB4^+^ (red) staining. **C)** Quantification of IBA1^+^ cells revealed that 7 d of PLX5622 eliminated ~85% of microglia and drug withdrawal stimulated rapid repopulation with IBA1^+^ cells. **D)** Repopulating cells had larger cell bodies compared to control microglia, which normalized to control levels over the course of repopulation. **E)** Repopulating microglia were also reactive for IB4, which again normalized to controls by 21 d repopulation. Data presented as means ± SEM. Statistical significance indicated as *p<0.05 and statistical trends as ^#^p<0.10.

**Supplemental Figure 2: Mouse body weights.** Mouse body weights shown for Con, Repop, Aged Con, and Aged Repop groups from the start to the cessation of CSF1R inhibitor treatment (n=9-10 mice/group). Data presented as raw means ± SEM and statistical significance indicated as *p<0.05, **p<0.01, and ***p<0.001.

**References:**

Barrett, R. M., Malvaez, M., Kramar, E., Matheos, D. P., Arrizon, A., Cabrera, S. M., . . . Wood, M. A. (2011). Hippocampal focal knockout of CBP affects specific histone modifications, long-term potentiation, and long-term memory. *Neuropsychopharmacology, 36*(8), 1545-1556. doi:10.1038/npp.2011.61

Edgar, R., Domrachev, M., & Lash, A. E. (2002). Gene Expression Omnibus: NCBI gene expression and hybridization array data repository. *Nucleic Acids Res, 30*(1), 207-210.

Elmore, M. R., Lee, R. J., West, B. L., & Green, K. N. (2015). Characterizing newly repopulated microglia in the adult mouse: impacts on animal behavior, cell morphology, and neuroinflammation. *PLoS One, 10*(4), e0122912. doi:10.1371/journal.pone.0122912

Elmore, M. R. P., Najafi, A. R., Koike, M. A., Dagher, N. N., Spangenberg, E. E., Rice, R. A., . . . Green, K. N. (2014). Colony-stimulating factor 1 receptor signaling is necessary for microglia viability, unmasking a cell that rapidly repopulates the microglia-depleted adult brain. *Neuron, 82*, 380-397. doi:DOI: 10.1016/j.neuron.2014.02.040

Grafen, A., & Hails, R. (2002). Modern Statistics for the Life Sciences.

Peters, A., & Kaiserman-Abramof, I. R. (1970). The small pyramidal neuron of the rat cerebral cortex. The perikaryon, dendrites and spines. *Am J Anat, 127*(4), 321-355. doi:10.1002/aja.1001270402

Ruxton, G. D., & Beauchamp, G. (2008). Time for some a priori thinking about post hoc testing. *Behavioral Ecology, 19*(3), 690-693. doi:10.1093/beheco/arn020

Spangenberg, E. E., Lee, R. J., Najafi, A. R., Rice, R. A., Elmore, M. R., Blurton-Jones, M., . . . Green, K. N. (2016). Eliminating microglia in Alzheimer's mice prevents neuronal loss without modulating amyloid-beta pathology. *Brain, 139*(Pt 4), 1265-1281. doi:10.1093/brain/aww016
